
JOURNAL INFORMATION
==============================
NLM Title Abbreviation: Innov Surg Sci
Journal ID: Innov Surg Sci
NLM Journal ID: 101708165
Journal ID: iss

Innovative Surgical Sciences

EISSN: 2364-7485
Publisher: De Gruyter

ARTICLE INFORMATION
==============================
PMCID: PMC11917179
PMID: 40110102
DOI: 10.1515/iss-2025-2003
Article ID: iss-2025-2003
Article version: 1
Subjects: Article

Abstracts DGAV

Electronic publication date: 2025 Mar 19
Publication date: 2025 Mar
Volume: 10
Issue: Suppl1
First page: 113
Last page: 208
Copyright: © 2025 by Walter de Gruyter Berlin/Boston
Copyright year: 2025
Copyright holder: De Gruyter
License: This work is licensed under the Creative Commons Attribution 4.0 International License.
License URL: https://creativecommons.org/licenses/by/4.0/

Page count: 96

==============================

### Robotic bariatric surgery can be established safely within a 12 month period

ID: 726

**Kategorie: DGAV - Bariatrische Chirurgie: Innovationen in der medikamentösen und chirurgischen Behandlung**

Jan Hoppe[1], Jens Höppner[1], Firas Makdesi[1], Miljana Vladimirov[1], Michael Leitz[1]

[1]*Universtitätsklinikum OWL, Campus Lippe, Universitätsklinik für Allgemein- und Viszeralchirurgie, Detmold, Deutschland*

**Background:**
Robotic surgery is increasingly being used for various indications in visceral surgery. The utilization of robotic assistance systems is also increasing significantly in metabolic surgery. In our tertiary care center with an established robotic program in surgical oncology and a competence center for bariatric surgery, we commenced a program with robot-assisted bariatric surgery in January 2021.

**Materials and methods:**
We performed a retrospective analysis of robotic procedures in metabolic surgery in our centre beginning in 2021 until 2023. Analyzed parameters were: ratio of laparoscopic vs robotic procedures, operative time, length of hospital stay, complication rates and type of procedure.

**Results:**
Of a total of 111 procedures, 27 were performed robotically in 2021. This ratio was kept similar in the following years (40 of 123 in 2022, 28 of 139 of 2023). Initially, there was no selection of patients. Length of stay and, in particular, complication rates did not show any significant difference. Duration of operation (including docking times) in average was 20 minutes longer in comparison between robotic and laparoscopic procedures. This OR time difference remained, however, we performed a higher percentage of complex procedures with the robot in the later years. In the subgroup of more complex bariatric procedure (roux-en-y gastric bypass) we could show a reduction in OR time in the following years from 123 min to 105 min in average while laparoscopic times kept stable around 92 to 99 minutes.

**Conclusion:**
In the appropriate setting and with existing expertise, robotic bariatric surgery can be safely established within 12 months and the learning curve can largely be overcome after around 30-50 cases. OR time reduction can be seen in the subgroup of more complex cases over a period of three years, indicating a potentially longer learning curve in these procedures. OR times in robotic bariatric surgery are slightly longer than in the laparoscopic setting.

Open Access. © 2025 The Author(s), published by De Gruyter. CC BY This work is licensed under the Creative Commons Attribution 4.0 International License.

**Preliminary Results of 15 patients with Toupet-Sleeve-Gastrectomy**
ID: 727

**Kategorie: DGAV - Bariatrische Chirurgie: Innovationen in der medikamentösen und chirurgischen Behandlung**

Martin Hoffmann[1], Eva Bungert[1], Thomas Haist[1], Petra Wirsing[1]

[1]*Asklepios Paulinen Klinik, Adipositaszentrum Wiesbaden, Wiesbaden, Deutschland*

**Background:**
GERD is a known complication of laparoscopic sleeve gastrectomy. Hiatoplasty alone is not sufficient to treat pre-existing or de-novo reflux. Sleeve gastrectomy with fundoplication is a logical development of bariatric surgery. Nissen-fundoplication is associated with higher incidence of dysphagia. The current submission shows our preliminary results in the treatment and follow-up of our first 15 patients with laparoscopic Toupet-Sleeve Gastrectomy (TSG).

**Materials and methods:**
Fifteen patients (9 female, 6 male) were included in the analysis. Patients had preoperative workup with GERD-Q and SF-36 questionnaires, esophago-gastroscopy, pH-metry and manometry. Indication for TSG were known GERD, presence of hiatal hernia > 3 cm and patients´ choice. The patients had follow-up with questionnaires and esophago-gastroscopy. Median follow-up was 1,2 years.

**Results:**
All patients had significant weight loss. We observed significant improvement of GERD symptoms (epigastric pain/burning, heartburn, regurgitation, ENT-symptoms). Additionally quality of life was also significantly improved. No patient presented signs of dysphagia or delayed gastric emptying.

**Conclusion:**
Our results show no differences in total weight loss compared to control subjects with “classic” sleeve gastrectomy. The remission of GERD symptoms is statistically significant compared to the preoperative status. As both operative procedures (Toupet-fundoplication and Sleeve-gastrectomy) have their own learning curve and associated complications, surgeons that completed their learning curves should do the operation. Nevertheless, the technique offers promising results and rational addition to the therapeutic options of bariatric surgery.

**Surgical therapy of anastomotic ulcers after gastric bypass procedures.The use of the robotic technology in our own patient population and review of the literature.**
ID: 938

**Kategorie: DGAV - Bariatrische Chirurgie: Innovationen in der medikamentösen und chirurgischen Behandlung**

Miljana Vladimirov[1 2], Ayman Georgios[2], Firas Makdesi[2], Imane Zahidi[2], Jan Hoppe[2], Jens Höppner[2]

[1]*Universität Bielefeld-Campus Lippe, Allgemein- und Viszeralchirurgie, Detmold, Deutschland*,
[2]*Universität Bielefeld-Campus Lippe, Detmold*

**Background:**
Roux-en-Y gastric bypass (RYGB) is one of the most common operations in metabolic and bariatric surgery (MBS), while one anastomosis gastric bypass (OAGB) is less frequently carried out. Both are primarily performed laparoscopically. Robotic surgery has been successfully introduced into MBS and RYGB has been found to be an ideal procedure for the robotic technology. An anastomotic ulcer (Marginal ulcer, MU) is a potential complication following RYGB or OAGB, which typically occurs at or near the gastrojejunal (GJ) anastomosis 6–12 months after the surgery. Its incidence rate is reported between 1 and 16%. Rarely, MU may present with a complication from the ulceration, such as bleeding, perforation, or strictures which require acute treatment.

**Materials and methods:**
The surgical therapy of anastomotic ulcers after gastric bypass procedures will be presented based on our own patient population and a literature review.

**Results:**
Between the 1st of April 2024 and the 30st of September 2024 a total of 82 patients underwent MBS procedures, primary (n = 61) and revisional (n = 21). In 8 cases revision surgery was performed due to anastomotic ulcers. The operations were preferably carried out robot-assisted and required gastrojejunostomies were performed in a hand-sewn technique with the robotic platform. Anatomic pathological findings and surgical procedures, which were performed in the patients with MU are presented in the table. There was no anastomotic leak. Postoperative bleeding occurred in one patient. There was no mortality.

**Conclusion:**
Patients with MU may experience acute or chronic abdominal pain. Following diagnosis by endoscopy, management of non-complicated MU typically involves modification of risk factors and medical therapy focused on proton pump inhibitors. Revisional surgery should be considered in patients with ulcers refractory to medical treatment. Rarely, anastomotic ulcer may present with a complication from the ulceration, such as bleeding, perforation, or strictures. In case of complicated ulcers, surgical intervention is often required for the repair of the perforation or resection of the stricture. Potential treatment options include resection of the gastrojejunostomy (GJ), including the ulcer with a reduction of the size of the gastric pouch and redo of the anastomosis. It is being discussed that non-absorbable suture material, used in linear or circular staplers for the creation of the GJ, increases the risk of anastomotic ulcers. Using absorbable instead of non-absorbable sutures resulted in a significant decrease in the incidence of MU. Due to its technical advantages, the robot-assisted approach is particularly advantageous in revision MBS procedures. A totally robotic approach in revisional surgery can be performed safely, and a robotic, hand-sewn GJ with absorbable suture material might reduce the risk of an anastomotic ulcer.

| | n |
|---|---|
| Patients | 8 |
| Uncomplicated ulcer | 1 |
| Complicated ulcer | |
| Penetration/Perforation | 6 |
| Bleeding | 1 |
| | |
| Type of previous bariatric surgeries | |
| RYGB | 1 |
| OAGB | 3 |
| Multiple bariatric surgeries | 4 |
| | |
| Revisional procedures | |
| Conversion OAGB to RYGB | 3 |
| Resection of GJ, gastric pouch downsizing, Redo GJ | 4 |
| Ulcer oversewing | 1 |
| Additional resection of gastric remnant | 5 |

**Table**

**Transduodenal resection of a large papillary tumor by means of intraoperative cholangioscopy.**
ID: 170

**Kategorie: DGAV - Chirurgische Endoskopie und Sonografie: Weiterbildung und Stellenwert der KI**

Mohamed El-Mahrouk[1], Azab Mohamed El-Shabrawi[1], Cord Langner[2], Hans Michael Hau[1], Robert Sucher[1]

[1]*Medizinische Universität Graz, Allgemein-, Viszeral- und Transplantationschirurgie, Graz, Österreich*,
[2]*Medizinische Universität Graz, Diagnostik- & Forschungsinstitut für Pathologie, Graz, Österreich*

**Background:**

Papillary adenomas are very rare benign tumors of the gastrointestinal tract. If manageable, purely endoscopic resection is favored. As an alternative, surgical resection via ampullectomy or pancreaticoduodenectomy can be performed. Often, the depth of infiltration cannot be assessed with sufficient precision, leading to pancreaticoduodenectomy for safety reasons.

**Materials and methods:**

We present the case of a 77-year-old patient in whom a transduodenal papillary resection of a large papillary adenoma was performed, after two unsuccessful endoscopic attempts. Intraoperative cholangioscopy was employed for surgical guidance, and to assess the depth of infiltration macroscopically.

**Results:**

Intraoperatively, an approximately 3 cm large papillary adenoma was identified. The depth of infiltration into the Vater's papilla was evaluated through intraoperative cholangioscopy. Due to the shallow depth of invasion, a papillary resection was performed, allowing for complete tumor removal. The postoperative course was uneventful, and the patient was discharged to home care on the 14th postoperative day. Subsequent healing progressed without complications, and the patient has been symptom-free since.

**Conclusion:**
Intraoperative cholangiography can be a valuable tool for assessing the depth of infiltration of large papillary adenomas, thereby enabling surgeons to make more informed decisions about the extent of resection.

**Endoscopic ultrasonography(EUS)-guided therapy of cholangiolithiasis (CAL) in surgically altered anatomy of the upper GI tract - a unicenter case study describing real-world data**
ID: 589

**Kategorie: DGAV - Chirurgische Endoskopie und Sonografie: Weiterbildung und Stellenwert der KI**

Frank Füldner[1], Frank Meyer[2], Uwe Will[1]

[1]*Dept. of Gastroenterology, Hepatology, Diabetology and General Internal Medicine, Municipal Hospital ("SRH Wald-Klinikum Gera"), Gera, Deutschland*, [2]*Dept. of General, Abdominal, Vascular and Transplant Surgery, Otto-von-Guericke University with University Hospital, Magdeburg, Deutschland*

**Background:**
For the treatment of cholangiolithiasis, ERCP can be considered the gold standard. In cases of surgically altered anatomy of the upper GI tract with or without a biliodigestive anastomosis, conventional ERCP using a duodenoscope can often be frustrating and unsuccessful. Therapeutic alternatives are ERCP using balloon-enteroscope, PTCD or even surgical intervention. Due to long-term experiences in EUS-guided techniques for drainage of the biliary system, the novel and favorable option of an endoscopic ultrasonography (EUS)-guided antegrade or even retrograde gall stone extraction via a transhepatic route.

**Materials and methods:**
All consecutive patients with cholangiolithiasis and surgically altered anatomy of the upper GI tract with and without previous attempts of an ERCP were enrolled in this unicenter case study over a defined period of time and were characterized with regard to the technical and clinical success of this approach.

**Results:**
From 2004 to 03/2020 overall 449 patients underwent EUS-guided cholangiodrainage in the reporting center. Among them there were 37 patients with cholangiolithiasis. In 8 of these 37 patients, gall stone extraction was achieved using EUS-ERCP rendezvous technique who were not included in this study since there was no surgically altered anatomy of the upper GI tract. In 13 of the remaining 29 subjects (45 %), there was a failure of previous attempts to reach the papilla of Vater od biliodigestive anastomosis using balloon-enteroscopy-guided ERCP. EUS-guided access to the biliary system was achieved in all 29 patients. Stone extraction was performed in 26 individuals (90 %) by means of antegrade push-technique after balloon dilatation of the papilla of Vater and biliodigestive anastomosis, respectively, before. In 11/29 cases (42 %), double pigtail prostheses were subsequently placed to track papilla of Vater / biliodigestive anastomosis (“ring drainage“), which were removed with gastroscopy three months later after previous ultrasound- and lab parameter-based follow-up control. In two patients (7 %), gall stones were extracted via a retrograde route using a transhepatic access site; in one patient (3 %), stone were removed by means of a combined antegrade and retrograde technique. In two subjects (7 %), cholangioscopy with electrohydraulic lithotripsy was used. Technical as well as clinical success rate was 100 % (29 of 29 patients). Re-interventions became necessary in 6/29 cases (21 %), complications occurred in 6 individuals (21 %).

**Conclusion:**
EUS-guided stone extraction in antegrade or retrograde technique for patients with surgically altered anatomy of the upper GI tract can be considered a favorable and safe but challenging approach of interventional endoscopy/EUS. It can provide a high technical and clinical success and low complication rates. In addition, it has the potential to substitute the time-consuming balloon-enteroscopy-guided ERCP as well as, in particular, PTCD and, thus, secundary and tertiary therapeutic alternatives.

### Vacuum Stent Therapy for Anastomotic Leakage Following Upper Gastrointestinal Surgery - A Retrospective Single-Center Analysis

ID: 735

**Kategorie: DGAV - Chirurgische Endoskopie und Sonografie: Weiterbildung und Stellenwert der KI**

Kai Jansen[1], Johanna Miller[1], Dominik Rieschl[1], Peter Wilhelm[2], Andreas Kirschniak[1], Jens Rolinger[1]

[1]*Klinik für Allgemein- und Viszeralchirurgie, Kliniken Maria Hilf GmbH, Mönchengladbach, Deutschland*, [2]*Bauch- und Enddarmzentrum, Sindelfingen, Deutschland*

**Background:**
Anastomotic leakage following upper gastrointestinal surgery represents a serious complication associated with increased morbidity, mortality, and prolonged hospital stays. Various endoluminal treatment options, including endoscopic vacuum therapy (EVT), open-pore film drainage (OFD), Over-the-Scope-Clips (OTSC) and self-expanding metal stent (SEMS) have been established over the past two decades. More recently, vacuum stent therapy has emerged as a promising minimally invasive method to effectively manage anastomotic leakage. However, the literature on this topic remains limited. This retrospective single-center analysis evaluates the clinical outcomes of patients treated with vacuum stent therapy for anastomotic leakage following upper gastrointestinal surgery.

**Materials and methods:**
This retrospective case series includes eleven cases of anastomotic leakage following upper gastrointestinal procedures treated with a vacuum stent between 2022 and 2024 at a single center institution. The analysis focused on short-term outcomes, including defect closure rates, number of therapy cycles, treatment duration and complications.

**Results:**
All 11 patients experienced anastomotic leakage following oncologic esophageal resection, with all (11/11) having previously undergone EVT or OFD before vacuum stent placement. The rate of successful primary defect closure under vacuum stent therapy was 54.5 % (6/11 patients), with mean treatment duration of 11.4 days (range 3 – 22 days). A mean of 1.7 therapy cycles (1 – 3 cycles) were required per patient. All persistent tissue defects were successfully healed secondarily through the use of alternative therapeutic measures, such as EVT, OFD, OTSC, and SEMS. In 100 % (11/11 patients), at least non-solid food intake was possible during treatment. The 90-day postoperative mortality rate was 9.1% (1/11 patients). Complications occurred in three patients, including one case of local bleeding and two cases of stent dislocation, all requiring re-endoscopy.

**Conclusion:**
Vacuum stent therapy is safe and effective method for managing anastomotic leakage following upper gastrointestinal surgery. Successful outcomes depend on careful consideration of defect characteristics (craniocaudal extent and localization), appropriate feeding protocols, and timely adjustments to treatment strategies. Further research is needed to determine the most suitable procedure for specific situations.

### Functional magnetic resonance imaging (fMRI) for the evaluation of postoperative liver remnant volume in ALPPS procedure - Case Report

ID: 167

**Kategorie: DGAV - Leber-/Galle-/Pankreaschirurgie: Technische Innovationen und KI**

Mohamed El-Mahrouk[1], Emina Talakic[2], Andri Lederer[1], Hans Michael Hau[1], Robert Sucher[1]

[1]*Medizinische Universität Graz, Allgemein-, Viszeral- und Transplantationschirurgie, Graz, Österreich*,
[2]*Medizinische Universität Graz, Radiologie, Graz, Österreich*

**Background:**

Portal venous embolization (PVE) and in-situ split liver resection (ALPPS) are suitable procedures for augmenting the postoperative residual liver volume (FLR). The aim is to increase the FLR to over 25 percent of the total liver volume. Various techniques such as CT/MRI volumetry in combination with functional LIMAX measurements or ICG clearance can be used to evaluate the FLR. Functional MRI with the contrast agent Primovist is a new radiological method for evaluating liver function in ALPPS procedures.

**Materials and methods:**

We present the case of a patient with a progressively enlarging liver tumor, measuring 10 x 8.7 cm, located in segments IV and V. An ALPPS procedure was performed due to an insufficient future liver remnant (FLR) in segments I, II, and III.

**Results:**

A 73-year-old female patient was admitted in January 2024 for inpatient care due to a progressively enlarging, suspicious liver tumor. A preoperative biopsy with histological evaluation was not performed. Due to a small left liver lobe, an ALPPS procedure was performed. During the first stage, intraoperative ligation of the posterolateral sector of the right portal vein stromal area was performed, along with extra-anatomical parenchymal dissection between segments III and IVb, and between segments VIII and IVa. On the 7th postoperative day, functional MRI with Primovist revealed significant hypertrophy of the left liver lobe (FLR > 25%). The second stage of the procedure was completed on the 9th postoperative day without complications. Hyperspectral imaging, as part of “image and function guided surgery,” was utilized. Postoperative intensive care was required for one day after each surgical stage. The operative times were 180 minutes for ALPPS Step 1 and 270 minutes for ALPPS Step 2. The patient was discharged on the 9th postoperative day following the completion of surgery, in good general condition and without complications. Histopathological analysis revealed a moderately differentiated hepatocellular carcinoma (HCC), exhibiting both trabecular and pseudoglandular patterns, which was resected with clear margins (R0).

**Conclusion:**

The ALPPS procedure provides an effective treatment option for achieving postoperative FLR increase, even in patients with liver cirrhosis. Functional magnetic resonance imaging can be utilized to assess liver function before proceeding with the second stage of ALPPS.

Abstracts – DCK 2025 – 142nd Congress of the German Society of Surgery – Munich, March 26–28 • DOI 10.1515/iss-2025-2003
Innov Surg Sci 2025; 10 (Special Suppl 1): s113–s208

**Assessing the usefulness of QR-code accessed surgical training videos on expert tutored open and robotic pancreatico- and hepatojejunostomy training**
ID: 188

**Kategorie: DGAV - Leber-/Galle-/Pankreaschirurgie: Technische Innovationen und KI**

Anas Amin Preukschas[1], Amila Cizmic[1], Harry van Goor[2], Stefan Bouwense[3], Maxime Dewulf[3], Anna Nießen[1], Thilo Hackert[1], Felix Nickel[1]

[1]*Klinik für Allgemein-, Viszeral- und Thoraxchirurgie - Universitätsklinikum Hamburg-Eppendorf - UKE, Hamburg, Deutschland*, [2]*Radboudumc, Nijmegen, Niederlande*, [3]*Akademisches Krankenhaus Maastricht, Maastricht, Niederlande*

**Background:**
Complex anastomoses like the pancreatico- and hepaticojejunostomy require repetitive training and time to master. Expert tutored surgical training courses to teach these anastomoses are limited in time and are costly. This study aims to evaluate the usefulness of easily accessible surgical training videos in open and robotic hepatopancreaticobiliary surgery for these anastomoses in the context of expert tutored surgical training courses.

**Materials and methods:**
Trainees and tutors at the Davos Course for HPB-Surgery (April 6th – April 9th 2024) and at the Hands-On-Course of the North-German Surgical Association (June 5th, 2024) were presented with QR-codes giving access to surgical training videos of open and robotic pancreatico- and hepatojejunostomy with 3D biotissue organs and additionally real intraoperative videos for the robotic anastomoses. After watching the videos and performing open or robotic anastomoses participants were asked to anonymously fill out online forms evaluating the usefulness of the videos. The data was collected using Google Form™ (Google LLC, USA) and descriptive statistics were generated using Microsoft Excel™ (Microsoft Corporation, USA) for the open and robotic surgical training group. The answers “Agree” and “Totally agree” was noted as a positive response on the Likert-scale.

**Results:**
A total of 35 participants were included (17 in the robotic training group, and 18 in the open training group). In the robotic group 100% of the participants found the QR-codes access to the videos easy to use. 100% found the training videos with intraoperative anastomoses and 89% the training videos with 3D biotissue organs useful. 100% could easily comprehend the steps of the operation with the intraoperative videos and 88% with the 3D biotissue organs videos. 100% found that the videos provided an added benefit to the expert tutoring. Only 19% found that the training videos could replace expert tutoring and 6% preferred only expert tutoring, without the training videos. 100% preferred both expert tutoring and training videos combined. Specifically for surgical training on the 3D biotissue organ models 71% preferred the videos with the 3D biotissue organs and 69% the videos with the intraoperative anastomoses. For the preparation to perform the procedure on real patients 47% preferred the videos with the 3D biotissue organs and 75% the videos from the real operation. 41% thought that they can efficiently train on their own and 65% thought that they can improve their surgical skill using the 3D biotissue organs and the videos without expert tutoring. 94% however found that expert tutoring is more effective when additionally using the training videos. 25% wanted more detailed training videos and 6% preferred surgical books to the videos. 59% found that training with the 3D biotissue organs and the training videos is more useful than procedural training on the robotic simulator. 100% would prefer access to training videos before expert tutoring and 94% preferred access to the videos and the 3D biotissue organs before expert tutoring.
In the open surgery group 94% of the participants found the QR-codes access to the videos easy and found the learning videos with the 3D biotissue organs useful and could easily comprehend the operation steps portrayed in the video. 100% found that the videos provided an added benefit to the expert tutoring. Only 44% found that the training videos could replace expert tutoring and 25% preferred only expert tutoring, without the training videos.94% preferred both expert tutoring and training videos combined. 40% thought that they can efficiently train on their own and 53% thought that they can improve their surgical skill using the 3D biotissue organ models and the videos without expert tutoring. 78% found that expert tutoring is more effective when additionally using the training videos. 40% wanted more detailed training videos and 13% preferred surgical books to the videos. 94% would prefer access to training videos before expert tutoring and 88% preferred access to the videos and the 3D biotissue organs before expert tutoring.

**Conclusion:**
Most participants preferred both the training videos and expert tutoring in combination for learning complex anastomoses. They preferred previous access to the videos before participating in the course. Training videos and 3D biotissue organs were seen as a very useful and necessary addition but were not considered sufficient for self-directed training without tutors at all. A follow-up randomized study comparing video and expert-tutoring vs. expert-tutoring alone will be conducted.

### Comparative Evaluation of Endoscopic Ultrasonography and Cross-Sectional Imaging in Preoperative Diagnosis of Intraductal Papillary Mucinous Neoplasms

ID: 334

**Kategorie: DGAV - Leber-/Galle-/Pankreaschirurgie: Technische Innovationen und KI**

Yannick Meyer[1], Ughur Aghamaliyev[1], Athanasios Zamparas[1], Simon Sirtl[2], Gwendolin Seidel[1], Georg Beyer[2], Gizem Abaci[3], Philipp Kazmierczak[3], Jens Werner[1], Bernhard W. Renz[1]

[1]*Klinikum der Universität München, Klinik für Allgemein-, Viszeral- und Transplantationschirurgie, München, Deutschland*, [2]*Klinikum der Universität München, Medizinische Klinik und Poliklinik 2, München, Deutschland*, [3]*Klinikum der Universität München, Klinik und Poliklinik für Radiologie, München, Deutschland*

### Background:

Intraductal papillary mucinous neoplasms (IPMNs) are precancerous lesions that carry a risk of progressing to malignancy. The probability of high-grade dysplasia and invasive cancer (IC) is approximately 31% for branch duct IPMNs and 62% for main duct IPMNs. Two of the four high-risk indicators for high-grade dysplasia or invasive carcinoma in IPMN include a pancreatic main duct dilated to more than 10 mm and a solid component exceeding 5 mm, both of which can be evaluated using CT or MRI/MRCP. Additionally, cyst size, which is an important worrisome feature, is routinely assessed through these diagnostic modalities. The latest Kyoto guidelines on IPMN management also recommend incorporating endoscopic ultrasonography (EUS) into the diagnostic process. Consequently, our study aimed to assess the sensitivity and specificity of EUS in the context of preoperative diagnostics.

### Materials and methods:

Consecutive patients who underwent pancreaticoduodenectomy (PD), distal pancreatectomy (DP), or enucleation for IPMN at our hospital between January 2014 and December 2023 were retrospectively identified. Patients who underwent total pancreatectomy were excluded from the study, as the indication for surgery in these cases was the presence of multiple cysts. Clinical data, including patient demographics, preoperative imaging (CT, MRI/MRCP, and EUS), tumor markers, and histopathology, were collected. Patients were subsequently stratified into two groups: those with low-grade dysplasia and those with high-grade dysplasia or invasive carcinoma. The McNemar test was used to evaluate differences in measurements between the two diagnostic modalities.

### Results:

A total of 176 patients underwent pancreatic resection because of IPMN at our institution between January 2014 and December 2023. Of these, 100 patients with available preoperative EUS were selected for further investigation. The comparison of patients with and without EUS revealed no significant difference. The median age of the patients was 72 (range 33–85) years, and there were 40 men (40%) and 60 women (60%). Malignant IPMN was seen in 28 of 62 patients (45,2%) with MD-IPMN and 17 of 38 patients (44,7%) with BD-IPMN. The Pearson correlation analysis showed a slightly better strong positive correlation between the pathological cyst size and CT/MRI than with EUS (CT/MRI: $r=0{,}593$, $p<0{,}001$; EUS: $r=0{,}564$, $p<0{,}001$). The McNemar test for MD-IPMN indicated no significant differences between EUS and CT/MRI in assessing cyst size ($p = 0{,}727$), solid components larger than 5 mm ($p = 0{,}063$), or MPD dilation > 10 mm ($p = 0.687$). Similarly, for BD-IPMN showed no statistically significant differences in cyst size, presence of a solid component greater than 5 mm, or main pancreatic duct (MPD) dilation > 10 mm between EUS and CT/MRI measurements ($p = 1{,}0$ for all comparisons).

### Conclusion:

There was no statistically significant difference in the data collection of the Kyoto criteria cyst size, pancreatic main duct dilated to more than 10 mm and a solid component exceeding 5 mm. This suggests that EUS and cross-sectional imaging modalities are largely consistent in assessing the Kyoto criteria.

### Comparison Of Cinematic Rendering And Computer Tomography For Preoperative Evaluation Of Vascular Invasion In Pancreatic Neoplasia

ID: 395

**Kategorie: DGAV - Leber-/Galle-/Pankreaschirurgie: Technische Innovationen und KI**

Justus Baecker[1], Vivien Wessel[2], Stephan Kersting[1]

[1]*Universitätsmedizin Greifswald, Klinik und Poliklinik für Allgemeine Chirurgie, Viszeral-, Thorax- und Gefäßchirurgie, Greifswald, Deutschland*, [2]*Universitätsmedizin Greifswald, Greifswald, Deutschland*

**Background:**

Surgeons rely on three-dimensional vision during procedures. Therefore, using 3D tools for preoperative planning may enhance intuitive decision-making. Cinematic Anatomy (CA), a novel 3D visualization technology, addresses this by using cinematic rendering (CR) to process DICOM CT/MRI data, creating photorealistic images. These images can be customized and refined using various tools.

In pancreatic surgery, vascular invasion significantly affects patient outcomes, including perioperative mortality and the one-year survival rate. CR has shown potential in improving pancreatic tumor visualization (Chu, 2018) needed for preoperative planning.

This study examines whether CR is equivalent or superior to conventional CT imaging in preoperative assessment of pancreatic neoplasms.

**Materials and methods:**

We conducted a monocentric, prospective, randomized, controlled cross-over study with retrospective cases. We chose a sample size of 12 patients with resectable pancreatic neoplasms in the head or body of the pancreas for initial evaluation. Three groups of four physicians - experienced pancreatic surgeons, general surgery residents, and radiologists evaluate each case twice: once using CT and once using CR.

The primary endpoint is the assessment of tumor resectability (yes/no). Secondary endpoints include accuracy in identifying vascular invasion, time to diagnosis, the impact of physician experience, and the ease of use of CA. Data is collected via standardized paper-based questionnaires, which assess resectability, vascular infiltration, time to diagnosis, and ease of use of CA software using a Likert scale. A two-week washout period between assessments ensures unbiased evaluations. Post-operative pathology and surgical reports are used as baseline validation between CA and standard CT.

**Results:**

Building on prior proof-of-concept research (Elshafei et al., 2019), which demonstrated that CR outperformed CT in terms of time and accuracy for preoperative planning, this study will compare the accuracy of vascular invasion assessment, diagnosis time, and user experience across varying expertise levels using both CR and CT. We hypothesize that CR will demonstrate superiority, particularly by reducing diagnosis time and being less dependent on physician experience. Additionally, we expect a stronger effect of CA usage among the surgical groups compared to the radiologist group.

**Conclusion:**

This study will demonstrate CR's potential in improving preoperative planning and surgical decision-making, particularly in cases of vascular invasion during pancreatic surgery. The use of CR could lead to optimized surgical strategies tailored to individual anatomical conditions, enhancing overall surgical outcomes in visceral surgery.

**References:**

1. Chu et al. (17. March 2018), Cinematic rendering of pancreatic neoplasms: preliminary observation and opportunites. Springer Nature, S. Abdom Radiol (2018);43:3009–3015.

2. Elshafai et al. (29. May 2019), Comparison of Cinematic Rendering and Computer Tomography for Speed and Comprehension of Surgical Anatomy, JAMA Surgery (2019);154(8):738-744

**Analysis of histopathological markers in pancreatic ductal adenocarcinoma using AI-driven tools and a 3D-in-vivo tumor model**
ID: 415

**Kategorie: DGAV - Leber-/Galle-/Pankreaschirurgie: Technische Innovationen und KI**

Laura Lemberger[1 2], Nandar Aye[2], Benedikt Wagner[3], Francisco Cruz Cordero[4], Thiha Aung[1 2], Silke Härteis[1], Christina Hackl[3]

[1]*Universität Regensburg , Lehrstuhl für molekulare und zelluläre Anatomie , Regensburg , Deutschland*, [2]*Technische Hochschule Deggendorf , Deggendorf , Deutschland*, [3]*Universitätsklinikum Regensburg , Klinik und Poliklinik für Chirurgie , Regensburg, Deutschland*, [4]*Pathologie Deggendorf , Deggendorf , Deutschland*

**Background:**
Pancreatic ductal adenocarcinoma (PDAC) is the most common malignancy of the exocrine pancreas and a leading cause of cancer-related deaths globally. The disease is characterized by aggressive growth and high resistance to therapy, which contributes to its low 5-year survival rate of 2-9%. The tumor stroma, which can account for up to 90% of the tumor volume, promote invasion, metastasis and therapeutic resistance. Key histopathological markers, such as Ki-67 and p53, also allow a valuable insight into tumor aggressiveness and prognosis. High Ki-67 expression, a marker of cellular proliferation, is associated with poor differentiation, lymph node metastasis and lower overall survival. Additionally, mutations in the TP53 gene, which encodes the tumor suppressor protein p53, are common in PDAC. These mutations allow the tumor to evade programmed cell death and to proliferate uncontrollably. The immune microenvironment also plays a significant role in PDAC prognosis. The immune cell score (IC) evaluates the immune cell infiltration in the tumor. The IC score has emerged as a potential prognostic biomarker. Higher IC levels correlate with better survival rates.
Understanding these histopathological and immunological factors is essential for improving treatment strategies and patient outcomes in PDAC. To advance research and therapy, innovative models like the chorioallantoic membrane (CAM) model offer a 3D-in-vivo system for testing tumor behavior and drug efficacy. The CAM model provides an ethical and cost-effective alternative to traditional animal studies. The CAM is an extraembryonic, highly vascularzied membrane, which enables the cultivation of primary tumor material and subsequent treatment with chemotherapy. Furthermore, digital pathology plays a key role in enabling in-depth analysis of histopathological features. To optimize the evaluation of histopathological parameters, an AI-supported approach using QuPath was here implented. QuPath is an open-source software for the analysis of high-resolution digital 2D-scans of tissue samples.

**Materials and methods:**
The MTT assay was used to assess the effect of chemotherapy combinations on MIA PaCa-2 cell viability. Cells were treated with varying concentrations of FOLFIRI, FOLFOX and FOLFIRINOX. Cell viability was measured at 594nm after 24h, 48h, 72h, 96h and 120h after treatment.
A CAM assay was employed to cultivate primary PDAC patient tissue on the CAM and to inject chemotherapy. The tumor tissue was exoculated after 5 days for histological staining (H&E, Ki-67, p53). Histologically stained tumor tissues were analyzed using QuPath before and after growth on the CAM. The region of interest (ROI) was annotated and StarDist script was applied for precise nucleus segmentation. Key parameters like „PixelSize" were adjusted for optimal cell detection to generate IC, Ki-67 and p53 scores. The tumor stroma was analyzed using a trained PixelClassifier, separating stroma from healthy connective tissue. These analyses helped to evaluate chemotherapy effects by comparing immune response (IC score), cell proliferation (Ki-67 score), mutation rate (p53 score) and tumor stroma area (TSR score) across untreated and treated groups.

**Results:**
The IC score was significantly higher in chemotherapy-treated PDAC tissues compared to untreated or control tissues (treated with 5% glucose). Similar correlations were observed for Ki-67 and p53, where treated tumor tissues exhibited lower Ki-67 and p53 scores compared to untreated tissues. TSR scores were the highest in untreated tumor tissues. The results were consistent for all patients included in this study.

**Conclusion:**
This study could show for the first time the changes in composition of tumor stroma in individual, patient-derived tumor samples grown and treated on the CAM. The correlation analyses conducted in this study provide valuable insight into the relationship between various histopathological scores and treatment modalities. The results collectively demonstrate that the chemotherapies examined have a positive effect on the IC, Ki-67, p53 and TSR score in PDAC tissues. Treatment with tested chemotherapy regimens (FOLFIRI, FOLFOX, FOLFIRINOX) proved to be effective in development of high immune response and lowering tumor aggressiveness by reducing IC, Ki-67, p53 and TSR scores for all patients included in this study. Taken together, the AI-based analysis allows for more accurate stratification of patients based on their tumor`s biological profile, enabling treatment strategies that are specifically designed to address individual tumor behaviors and responses. In addition, this study proved successful in combining a 3D-in-vivo tumor model and AI analysis of important prognostic factors in PDAC. Looking ahead, developments like molecular tumor boards, combined with individualized therapy approaches, could further advance precise tumor treatments. This study`s use of the modern AI analysis tool QuPath highlights the potential of these technologies to optimize clinical assessments of key factors in PDAC, pointing the way toward more precise therapeutic strategies in the future.

### A multi-stage artificial intelligence (AI) model for the prediction of the pre-therapeutic lymph node status in pancreatic ductal adenocarcinoma.

ID: 489

**Kategorie: DGAV - Leber-/Galle-/Pankreaschirurgie: Technische Innovationen und KI**

Alexander Semaan[1], David Duenas Gaviria[2], Matthäus Felsenstein[3], Lena-Christin Conradi[4], Hyun Ko[5], Uli Fehrenbach[6], Dou MA[3], Philipp Leyendecker[1], Lara Braun[1], Lucrezia De Santis[1], Daniel Weissinger[1], Philipp Lingohr[1], Steffen Manekeller[1], Hanno Matthaei[1], Jörg Kalff[1], Patrick Kupczyk[7], Shadi Albarqouni[2]

[1]*University Hospital of Bonn, Clinic and Polyclinic for General, Visceral, Thoracic and Vascular Surgery, Bonn, Deutschland*, [2]*University Hospital of Bonn, Computational Medical Imaging Research, Department of Diagnostic and Interventional Radiology, Bonn, Deutschland*, [3]*Charité – Universitätsmedizin Berlin, Department of Surgery, Berlin, Deutschland*, [4]*University Hospital of Göttingen, Department of Surgery, Göttingen, Deutschland*, [5]*University of Melbourne Peter MacCallum Cancer Centre, Department of Radiology, Melbourne, Australia*, [6]*Charité – Universitätsmedizin Berlin, Department of Radiology, Berlin, Deutschland*, [7]*University Hospital of Bonn, Department of Radiology, Bonn, Deutschland*

**Background:**
Curative treatment for pancreatic ductal adenocarcinoma (PDAC) relies on surgical resection. The current standard for assessing surgical resectability is based on the International Association of Pancreatology (IAP) consensus ABC criteria, which takes into account anatomical, biological and conditional factors. The IAP guideline states that PT-CT or biopsy are viable options for determining lymph node status prior to resection. This is due to the fact that evaluation of standard contrast-enhanced CT scans has shown insufficient sensitivity for assessing nodal status. Therefore, we sought to investigate the potential improvement of preoperative lymph node assessment in pancreatic cancer (PDAC) using an AI model developed by our research group.

**Materials and methods:**
In a multicenter cohort of almost 1000 treatment-naive PDAC patients from Bonn, Berlin, Göttingen and Melbourne (Australia), the preoperative N-status of resected PDACs was predicted using a multimodal AI model. The model is based integrates both, radiological data from preoperative contrast-enhanced CT scans and clinical parameters including CA19-9 status and tumor diameter among others.

**Results:**
We demonstrate that the AI model described here significantly improves the identification of patients with positive nodal status compared to previously published or clinically used prediction models for preoperative nodal status in pancreatic cancer. This is achieved by a large multi-center patient cohort.

**Conclusion:**
The preoperative identification of node-positive patients with pancreatic ductal adenocarcinoma can be significantly improved using our multimodal AI-based prediction model, which provides additional biological information that can be applied to existing multimodal treatment concepts.

### Preoperative prediction of postoperative pancreatic fistula after pancreaticoduodenectomy using radiomics and machine learning based on computed tomographic diagnostics

ID: 737

**Kategorie: DGAV - Leber-/Galle-/Pankreaschirurgie: Technische Innovationen und KI**

Johannes Kaiser[1], Matthias Benndorf[1], Esther Anna Biesel[1], Claudia Neubauer[1], Stefan Fichtner-Feigl[1], Fabian Bamberg[1], Uwe A. Wittel[1], Jakob Neubauer[1]

[1]*Universitätsklinikum Freiburg, Freiburg, Deutschland*

**Background:**
Postoperative pancreatic fistula is one of the major complications after pancreatic head resection and can be life-threatening for patients. This study employed machine learning and radiomics to determine whether postoperative pancreatic fistulas (POPF) and perioperative drain amylase dynamics can be predicted prior to pancreaticoduodenectomy by evaluating the radiologic appearance of the pancreatic tissue.

**Materials and methods:**
In this retrospective trial 68 patients were included. For POPF prediction model (PPM) Radiomic features of the pancreas were extracted from the arterial phase of computed tomography (CT) at a 1 mm slice thickness for each patient. The radiomic features with highest correlation with POPF for our models, controlling for autocorrelation and applying Bonferroni correction for P-values were selected. For amylase prediction model (APM), radiomic features were correlated with postoperative maximum drain amylase levels at a cut-off of 1000 U/l. ROC analysis was performed for evaluation of the resulting prediction models.

**Results:**
POPF prediction model showed an area under the curve (AUC) of 0.897 (confidence interval (CI) = 82.3-97.1%) in the cohort. The AUC of PPM was higher than that for the Roberts score, but the difference was not statistically significant. An attempt to predict postoperative amylase dynamics in the drainage fluid achieved an AUC of 0.936 (CI = 88%-99.1%).

**Conclusion:**
Preoperative prediction of POPF and drain amylase dynamics using radiomics and machine learning showed promising results. Both models offer new approaches to the clinical management of POPF.

**Outcome analysis and deep learning adjusted risk stratification for patients with early and late onset primary liver carcinomas. A StuDoQ data analysis**
ID: 923

**Kategorie: DGAV - Leber-/Galle-/Pankreaschirurgie: Technische Innovationen und KI**

Nikolaus Börner[1], Dionysios Koliogiannis[1], Christina Frost[1], Jens Werner[1], Markus Guba[1]

[1]*Department of General, Visceral, and Transplant Surgery, , LMU, Munich, Deutschland*

**Background:**
Liver resection is a common treatment option for patients with primary liver carcinomas, both early and late onset. However, the outcome of this procedure can vary depending on several factors, including the presence of risk factors, perioperative mortality and morbidity, local lymphadenectomy, histological findings, and the extent of resection. This study aims to analyze these factors and their impact on the outcome of liver resection especially with the focus on late vs early onset carcinomas. Furthermore, a Deep learning adjusted risk stratification will be optimize preoperative planning.

**Materials and methods:**
Retrospective datanalysis from the StuDoQ-Database. 1511 patients with primary liver malignancy were analyzed. The cohort was divided into young and old patients using different age cutoffs of 50, 55, 60, and 65 years. Missing Data was imputed using a Deep learning driven imputation algorithm. The comparison focused on preoperative, perioperative and postoperative parameters, including preoperative lab values, ECOG and comorbidities and perioperative characteristics such as surgery technique, time and conversion rate. Postoperative variables included ECOG and Clavien classification, hospitalization time, complications and revisions. Statistical differences between young and old patients were analysed using Chi-squared or Fisher's exact test for discrete variables. Continuous parameters were analysed using t-Test, Welch's t-Test or Mann-Whitney-U test, depending on pre-tests of normality and the central limit theorem. Risk Stratification will be done using a Deep Learning driven algorithm.

**Results:**
Depending on the age cutoff, the prevalence of young patients was 20.8% (314 for cutoff c = 50 y), 31.2% (471 for c = 55 y), 44.6% (674 for c = 60 y), and 57.9% (875 for c = 65 y). With slight dependencies on age cutoff, the following preoperative parameters showed small, but statistically significant differences between young vs. old patients. The Results showed cutoff-independent significant differences for: CEA, CRP, y-GT, albumin and creatinine. Further BMI, p = 0.0001 for c = 55 y), alcohol consume (3.2% vs. 8.1%, p=0.0003 for c = 55 y), Child-Pugh score (2.6% vs. 9.4% having a score > 0, overall distribution difference p = 0.001 for c = 55 y). The postoperative parameters significant differences included the severity of 30-day surgical site infections (3.3% vs. 6.0% with Clavien II and III, p = 0.0008 for c = 55 y) and the postop-ECOG distribution, but only for age cutoff of 50 years (p = 0.018). There has been statistical indications, however no significant statistical difference for post-operative pulmonary embolism, - pneumonia, - stroke, - renal failure and operative conversion rate (Deep Learning based risk stratification currently performed).

**Conclusion:**
There is a significant difference between young and old patients with primary liver malignancies in their surgical outcome. By the use of the deep learning risk stratification, we will be able to further minimize operative risk and provide individualized surgical planning.

**Effect of Braun jejunojejunostomy on delayed gastric emptying in patients undergoing classic pancreaticoduodenectomy– a single center analysis**
ID: 953

**Kategorie: DGAV - Leber-/Galle-/Pankreaschirurgie: Technische Innovationen und KI**

Benjamin Müssle[1,2], Kira Steinkraus[2], Lena Weine[2], Marko Kornmann[2], Emrullah Birgin[2], Nuh Rahbari[2]

[1]*Ulm, Klinik für Allgemein- und Viszeralchirurgie, Ulm, Deutschland*, [2]*Klinik für Allgemein- und Viszeralchirurgie, Klinik für Allgemein- und Viszeralchirurgie, Ulm, Deutschland*

**Background:**
Classic pancreaticoduodenectomy (cPD) is the standard procedure for benign, malign and borderline tumours of the pancreatic head. Delayed gastric emptying (DGE) is a major complication after cPD, that impairs patients recovery and quality of life. Although DGE is not life threathening it affects morbidity and prolongs the length of hospital stay. Aim of the study was to investigate the impact of Braun-jejunojejunostomy on DGE.

**Materials and methods:**
Patients who underwent cPD between March 2021 and October 2023 were retrospectively analysed. The development of DGE was analysed for a 90-day period following surgery. Clinical outcomes related to DGE, including incidence rates, severity and length of hospital stay were evaluated using regression models.

**Results:**
The study investigated 89 patients undergoing cPD, 51 in the Braun group and 38 in the controll group. Overall, 19 (21%) patients developed DGE. Incidence of DGE was significantly lower in the Braun group compared to the contoll group 9.8% vs. 36.8%, respectively (p=0.0021). Except DGE we found no significant difference between the groups in the subtypes of complications. Furthermore, there was no significant difference in overall morbidity and mortality.

**Conclusion:**
Braun jejunojejunostomy helps to reduce the incidence and the severity of DGE in patients undergoing cPD. While the related mortality of DGE after cPD is low, it is associated with severe sequelae.

-

### Competency, Proficiency, and Mastery: Learning Curves of Robotic Distal Pancreatectomy in 16 International Expert Centers

ID: 206

**Kategorie: DGAV - Leber-/Galle-/Pankreaschirurgie: Wann minimalinvasiv/robotisch, wann offen?**

Philip Müller[1 1], Christoph Kümmerli[1], Adrian Billeter[1], Felix Nickel[2], Cristiano Guidetti[3], Christoph Tschuor[4], Jan Philipp Jonas[5], Olivier Saint Marc[6], Quintus Molenaar[7], Ulrich Friedrich Wellner[8], Tobias Keck[8], Beat Möckli[9], Christian Toso[9], Fabrizio Di Benedetto[3], Pier Giulianotti[10], Didier Roulin[11], John Martinie[11], Harish Lavu[12], Charles Yeo[12], David Kooby[13], Jin He[14], Ugo Boggi[15], Thilo Hackert[2], Beat Müller[1], Pierre Clavien[5]

[1]*Department of Surgery, Clarunis University Digestive Health Care Center,, University Hospital Basel, Basel, Schweiz*, [2]*Department of General, Visceral and Thoracic Surgery, University Medical Center Hamburg-Eppendorf, Hamburg, Deutschland*, [3]*Hepato-pancreato-biliary Surgery and Liver Transplantation Unit, University of Modena and Reggio Emilia, Modena, Italien*, [4]*Department of Surgery and Transplantation, Rigshospitalet, Copenhagen University Hospital, Copenhagen, Denmark*, [5]*Department of Surgery and Transplantation, University Hospital Zurich,, Zurich, Schweiz*, [6]*Service de Chirurgie Digestive, Endocrinienne et Thoracique, Centre Hospitalier Régional,, Orleans, Frankreich*, [7]*Department of Hepato-Pancreato-Biliary Surgery, Regional Academic Cancer Center Utrecht, St. Antonius Hospital Nieuwegein , University Medical Center Utrecht, Utrecht, Niederlande*, [8]*Department of Surgery, , University Medical Center Schleswig-Holstein, Campus Luebeck, , Luebeck, Deutschland*, [9]*Department of Surgery, University Hospitals of Geneva, Geneva, Schweiz*, [10]*Division of General, Minimally Invasive, and Robotic Surgery, Department of Surgery, , University of Illinois at Chicago, Chicago, Vereinigte Staaten*, [11]*Division of HPB Surgery, Department of Surgery, Carolinas Medical Center, Charlotte, Vereinigte Staaten*, [12]*Department of Surgery, Thomas Jefferson University Hospital, Philadelphia, Vereinigte Staaten*, [13]*Department of Surgery, Winship Cancer Institute, , Emory University School of Medicine, , Atlanta, Vereinigte Staaten*, [14]*Department of Surgery, Johns Hopkins University School of Medicine, Baltimore, Vereinigte Staaten*, [15]*Division of General and Transplant Surgery, University of Pisa , Pisa, Italien*

**Background:**
Robotic distal pancreatectomy (RDP) is an emerging minimally-invasive approach, however only limited and mostly single center data is available on its safe implementation including the learning curve. The aim of this study was to evaluate the different phases of the learning of curve of RDP in international expert centers.

**Materials and methods:**
Consecutive patients undergoing elective RDP from 16 expert centers and 3 continents were included to assess the learning curve. Based on the first 100 RDPs of each center, three cutoffs were used to define the learning curve: operative time for *competency*, major complications (Clavien-Dindo grade ≥III) for *proficiency*, and textbook outcome for *mastery*. Clinical outcomes before and after the cutoffs were compared.

**Results:**
From 2’403 RDP, the learning curve analysis was conducted on 1’109 RDPs. Competency, proficiency and mastery were reached after 46, 63 and 73 RDP procedures. After competency operative time decreased from 245 to 235 minutes (p=0.002). Reaching proficiency was reflected by a reduction of major complications from 18% to 10% (p=0.012) and mastery was associated with an increasing rate of patients reaching textbook outcome(63% to 71%; p=0.028). The POPF rate remained stable along the learning curve between 18.5% and 21.5%. Previous laparoscopic experience accelerated the learning process by reducing the operative time and an earlier decrease in major complications.

**Conclusion:**
*Competency*, *proficiency*, and *mastery* learning curves for RDP were reached after 46, 63, and 73 procedures in international expert centers. The findings highlight that the learning curves for intraoperative parameters are completed earlier, however extensive experience is needed to master RDP.

**Mesopancreatic excision during pancreatoduodenectomy**
ID: 303

**Kategorie: DGAV - Leber-/Galle-/Pankreaschirurgie: Wann minimalinvasiv/robotisch, wann offen?**

Sami-Alexander Safi[1], Lena Haeberle[2], Irene Esposito[2], Wolfram Trudo Knoefel[1]

[1]*Universitätsklinikum Düsseldorf, Abteilung für Allgemein-, Viszeral- und Kinderchirurgie, Düsseldorf, Deutschland*, [2]*Universitätsklinikum Düsseldorf, Institut für Pathologe, Düsseldorf, Deutschland*

**Background:**
Following implementation of the circumferential resection margin (CRM) into standard histopathological evaluation, tumor extensions were frequently underestimated at the dorsal and medial resection margins. One reason may be a negligence in mesopancreatic excision (MPE), synergistically to mesorectal or mesocolic excision to secure fascial integrity at these margins. We have previously studied the mesopancreas in both upfront and neoadjuvantly treated pancreatic cancer (PDAC) patients and revealed infiltration rates up to 80%. Mesopancreatic infiltration remained evident in localized PDAC patients, questioning current resectability criteria to predict true tumor extensions. The aim of this video abstract is to provide intraoperative evidence for a peripancreatic compartment anatomy during oncological pancreatoduodenectomy and to intraoperatively preserve this integrity before reaching a point of no return.

**Materials and methods:**
The intraoperative pathway during pancreatoduodenectomy with mesopancreatic excision was recorded in a patient with a primarily resectable pancreatic head cancer. The postoperative resectate will be presented including a standardized pathological inking and axial slicing technique according to the LEEP protocol. The resection margins are microscopically assessed, and their fascial integrity are microscopically visualized to provide evidence, both on a macroscopic and microscopic level, for a peripancreatic compartment anatomy.

**Results:**
Starting with the mobilization of the right colonic flexure and the sub-omental access to the greater sac, the told fascia is utilized as the first landmark and its pancreatic integrity is preserved until its craniomedial extensions towards the ventral aspect of the pancreatic head. The dissection is carried out until the right border of the superior mesenteric vein (SMV) and continued in depth towards the right border of the superior mesenteric artery (SMA). After a complete visualization of the ventral peripancreatic region is provided, dissections are continued with the Kocher maneuver. The Gerota fascia is used as an anatomic landmark with sharp preparations onto the inferior caval vein and the abdominal aorta. The caudal border is the root of the inferior mesenteric artery (IMA) and the dissection are continued cranially until the left renal vein is exposed and visualized. From this point the right diaphragmatic crus is visualized, to dissect through the root of the mesopancreas, to gain access to the aortic origin of the SMA. The Pancreatic head with the duodenum are retracted towards the left and dissections around the SMA are continued until its junction into the mesopancreas. Dissections are continued from the superior border of the pancreatic head. The lesser sac is opened and after dissections of the hepatoduodenal ligament are finalized, the common hepatic artery and the splenic artery are sharply followed until their origin of the celiac trunk (CT) are visualized. From there, dissections are continued to visualize the CT until the aortic origin of the left gastric artery and are finalized until the former diaphragmatic dissections from the posterior access are met.

**Conclusion:**
Utilizing the fascial system during early preparations in pancreatoduodenectomy allows to centrally visualize the, peripancreatic located, vascular system (SMA, CHA, CT, VMS and PV). Since the root of the SMA, CHA and CT are first verified for tumor infiltration, this achieves clarity for tumor extensions before reaching a surgical point of no return.

### Validation of the “Tampa Difficulty Score” for robotic hepatectomy in a german hepatobiliary center

ID: 468

### Kategorie: DGAV - Leber-/Galle-/Pankreaschirurgie: Wann minimalinvasiv/robotisch, wann offen?

Andreas Andreou[1], Haluk Morgül[1], Andreas Pascher[1], Shadi Katou[1], Benjamin Strücker[1]

[1]*Universitätsklinikum Münster, Klinik für Allgemein-, Viszeral- und. Transplantationschirurgie, Münster, Deutschland*

**Background:**
The Tampa Difficulty Score (TDS) has been recently introduced as the first difficulty scoring system (DSS) for robotic hepatectomy and was significantly associated with increased postoperative mortality and length of hospital stay. We aimed to validate the TDS in our patient cohort undergoing robotic liver resection.

**Materials and methods:**
Clinicopathological data of patients who underwent robotic hepatectomy for benign or malignant diseases between 2020 and 2024 in a major hepatobiliary center in Germany were assessed. Liver resections were classified according to the TDS considering factors such as location and size of the tumor, extent of resection, the need for portal lymphadenectomy, the need for biliary resection, the tumor entity, and the need for neoadjuvant chemotherapy.

**Results:**
During the study period, 102 patients underwent robotic hepatectomy. 17 patients was in the less demanding group (group 1), 81 patients were in the intermediate group (group 2), and 4 patients were in the more demanding group (group 3), respectively. Less demanding procedures were significantly associated with shorter duration of surgery (208 vs. 278 vs. 401 min, $p < 0.001$), lower complication rates (12% vs. 49% vs. 50% $p = 0.017$), and shorter length of hospital stay (5 vs. 6 vs. 10 days, $p = 0.015$) compared to intermediate and more demanding procedures. Major complications rate increased correspondingly to the increased difficulty grade; however the differences did not reach statistical significance (6% vs. 17% vs 25%, $p = 0.437$). There was only one postoperative mortality due to liver failure in the intermediate group and it was not associated with the TDS.

**Conclusion:**
Higher TDS is associated with longer duration of surgery, higher postoperative morbidity, and prolonged length of hospital stay and may be used as a helpful tool for individualized treatment planning, patient counseling, and robotic surgical education.

**Effective management of bilioenteric anastomosis leakage after major liver resection**
ID: 866

**Kategorie: DGAV - Leber-/Galle-/Pankreaschirurgie: Wann minimalinvasiv/robotisch, wann offen?**

Sepehr Abbasi Dezfouli[1], Elmira Heidenreich[1], Mohammadamin Shahrbaf[1], Elias Khajeh[1], De-Hua Chang[2], Markus Mieth[1], Arianeb Mehrabi[1]

[1]*Department of General, Visceral and Transplantation Surgery, Heidelberg University Hospital, Heidelberg, Germany, Heidelberg*, [2]*Department of Diagnostic and Interventional Radiology, Heidelberg University Hospital, Heidelberg, Germany, Heidelberg*

**Background:**

Post-hepatectomy bile leakage (PHBL) is a challenging issue that can lead to morbidities and mortality after liver resection. This leakage can occur either from a bilioenteric anastomosis (BEA) or from the transection surface of the liver, but most studies have not distinguished between the two. The aim of this study was to investigate the incidence, risk factors, and effective management of BEA leakage after major liver resection.

**Materials and methods:**

This retrospective study analyzed data from a hepatectomy database maintained prospectively between 2008 and 2023. All patients who underwent major liver resection with concurrent BEA were included. Bile leakage from BEA was diagnosed during percutaneous transhepatic cholangiography and drainage (PTCD), or during reoperation. BEA leakage in the first 7 postoperative days were treated with reoperation and after that with PTCD. Perioperative data and data on the postoperative management of patients with bile leakage were collected and analyzed.

**Results:**

Out of 2936 patients who underwent major liver resection, 229 also underwent BEA. Leakage from the BEA was identified in 44 (19.2%) of these patients. Patients with BEA leakage had a higher rate of post-hepatectomy hemorrhage ($P = 0.005$), major complications ($P = 0.001$), BEA stenosis ($P = 0.006$), and mortality ($P = 0.043$). Intensive care unit and hospital stay were also longer in patients with BEA leakage ($P = 0.001$ and $P < 0.001$, respectively). The success rate of BEA leakage management was 70% for reoperation and 58.3% for PTCD.

**Conclusion:**

BEA leakage after major liver resection was a severe complication and was associated with higher morbidity and mortality rates. Treatment was more successful with reoperation than with PTCD, although PTCD was a good additional therapy option after reoperation.

### Robotic total pancreatectomy with type II portal vein resection

ID: 178

**Kategorie: DGAV - Minimalinvasive und Robotergestützte Chirurgie: Onkologische Chirurgie in MIC und Robotik**

Valerie Isabel Nottberg[1], Jan Bardenhagen[1], Pasquale Scognamiglio[1], Faik G. Uzunoglu[1], Thilo Hackert[1], Felix Nickel[1]

[1]*Universitätsklinikum Hamburg-Eppendorf, Klinik für Allgemein-, Viszeral- und Thoraxchirurgie, Hamburg, Deutschland*

**Background:**
Pancreatic ductal adenocarcinoma (PDAC) often requires complex surgical strategies due to its aggressive nature and anatomical challenges.[1,2] Robotic-assisted surgery offers a promising alternative to traditional open procedures, potentially enhancing precision and reducing recovery times.[3,4] This case illustrates a robotic total pancreatectomy with type II portal vein resection in a patient with pancreatic body adenocarcinoma extending beyond the border of the gastroduodenal artery (GDA) and involvement of the portal vein (PV).

**Materials and methods:**
A 72-year-old male was planned for distal pancreatectomy (DP) based on upfront resectable PDAC on preoperative imaging, staged preoperatively as cT3, cN0, cM0, with a CA 19-9 level of 610.9 kU/l. Due to the tumor's extension to the gastroduodenal artery and involvement of the PV, a total pancreatectomy with portal vein resection was performed with type II reconstruction using a bovine pericardial patch. Postoperative management included a 24-hour stay in the Post Anesthesia Care Unit (PACU) followed by transfer to a standard care unit. The anticoagulation protocol started with therapeutic Heparin, transitioning to Eliquis to minimize thrombotic risks.

**Results:**
The operation was completed successfully with the robotic system, without intraoperative complications. Pathological analysis post-surgery indicated a stage UICC III cancer (G2, pT3, pN2 (4/30), Pn1 L1 V1 R1), confirming more extensive disease than initially staged. The patient showed an uneventful recovery except for a delayed gastric emptying resolving under conservative treatment and was discharged on postoperative day 14. Postoperative staging justified the recommendation for adjuvant chemotherapy with mFOLFIRINOX.

**Conclusion:**
This case highlights the feasibility of robotic-assisted pancreatectomy for managing even more advanced pancreatic cancer, with effective perioperative outcomes and adherence to oncological principles. This surgical approach, facilitated by robotic technology, underscores a significant advance in the surgical management of pancreatic adenocarcinoma, providing a foundation for improved patient outcomes in complex cases.

[1] McGuigan A, Kelly P, Turkington RC, Jones C, Coleman HG, McCain RS. Pancreatic cancer: A review of clinical diagnosis, epidemiology, treatment and outcomes. World J Gastroenterol. 2018 Nov 21;24(43):4846-4861. doi: 10.3748/wjg.v24.i43.4846. PMID: 30487695; PMCID: PMC6250924.
[2] Isaji S, Mizuno S, Windsor JA, Bassi C, Fernández-Del Castillo C, Hackert T, Hayasaki A, Katz MHG, Kim SW, Kishiwada M, Kitagawa H, Michalski CW, Wolfgang CL. International consensus on definition and criteria of borderline resectable pancreatic ductal adenocarcinoma 2017. Pancreatology. 2018 Jan;18(1):2-11. doi: 10.1016/j.pan.2017.11.011. Epub 2017 Nov 22. PMID: 29191513.
[3] Luo YC, Yang TY, Li W, Yu QJ, Xia X, Lin ZY, Chen RD, Cheng L. Perioperative and oncologic outcomes of robot-assisted versus open surgery for pancreatic ductal adenocarcinoma: a systematic review and meta-analysis. J Robot Surg. 2024 Jul 22;18(1):288. doi: 10.1007/s11701-024-02046-x. PMID: 39039276.
[4] Hackert T, Klaiber U, Pausch T, Mihaljevic AL, Büchler MW. Fifty Years of Surgery for Pancreatic Cancer. Pancreas. 2020 Sep;49(8):1005-1013. doi: 10.1097/MPA.0000000000001634. PMID: 32833940.

### For safe robotic-assisted thoracoscopic esophagectomy
ID: 362

**Kategorie: DGAV - Minimalinvasive und Robotergestützte Chirurgie: Onkologische Chirurgie in MIC und Robotik**

Hiroshi Okamoto[1], Takashi Kamei[1]

[1]*Tohoku University, Sendai, Japan*

**Background:**
Robotic-assisted minimally invasive esophagectomy (RAMIE), which has become explosively widespread in Japan since the start of insurance coverage in 2018, offers the advantages of immersion in a three-dimensional magnified view of DaVinci® system and excellent operability features such as anti-shake, joint function, and non-reliance on eye–hand coordination. Nevertheless, the technology has the following disadvantages:

1-a. Interference between the robotic arms

1-b. Interference between the robotic arm and the patient

1-c. Interference between the robotic arm and the assistant

2. Organ damage caused by excessive stress on organs due to the lack of tactile sensation or organ damage caused by robotic arms outside or beyond the field of view

3. Thermal damage caused by operational errors in the energy devices

4. Prolonged operative time in some surgical fields due to camera angle limitations

The surgeon must fully recognize the disadvantages listed above.

**Materials and methods:**
This manuscript presents the safety precautions and pitfalls experienced in approximately 180 cases to date, using thoracic procedures in esophageal cancer surgery as examples for facilities and young surgeons considering the introduction of RAMIE.

**Results:**
1-a. In the thoracic region, port spacing tends to become narrow depending on body shape. Particular attention should be paid to interference between the robotic arms when dissecting the left upper longitudinal contour, where the arms are situated close to each other. This is sometimes addressed by changing the positions of the camera and arms.

1-b. In the lower mediastinum, the fourth arm may sometimes interfere with the patient’s right shoulder. To avoid such interference, we are careful about the right shoulder of the patient and protected with Ortho-Glass® for plaster casts when holding semiprone position.

1-c. In our department, assistant help prepare the surgical field and use staplers. However, great care must be taken to preclude the assistant’s arms and devices from interfering with robotic arms. We have an experience almost damaging the azygos arch once when the robotic arm pushed against the assistant’s arm.

2. We experienced events associated with intraoperative procedures, including tracheobronchial injury due to excessive dissection, vertebral injury outside the visual field, postoperative cardiac tamponade due to excessive compression of the pericardium surface, and unintended compression of the right subclavian artery and the right recurrent laryngeal nerve.

3. We experienced thermal damage to the recurrent laryngeal nerve due to an operational error with a double bipolar device and thermal damage to the trachea with a monopolar device.

4. Visualizing the left esophagus is difficult and time-consuming. It is advised that such difficult cases should be avoided at the time of introduction.

**Conclusion:**
As RAMIE becomes more widespread, surgeons and assistants will need to communicate with each other constantly to ensure a safe operation and avoid possible complications.

### Experiences in Robotic (non-)Malignant Colorectal Surgery: Comprehensive Insights from a two-center Analysis Using the Senhance Robotic System

ID: 779

**Kategorie: DGAV - Minimalinvasive und Robotergestützte Chirurgie: Onkologische Chirurgie in MIC und Robotik**

Vivianda Menke[1], Narimantas Samalavicius[2], Vaida Nausediene[2], Rita Karpiciute[2], Frank Willeke[3], Olaf Hansen[1]

[1]*Evangelisches Krankenhaus Wesel, Department of Surgery, Wesel, Deutschland*, [2]*Klaipėda University Hospital, Klaipėda, Lithuania*, [3]*Marien Kliniken Siegen, Siegen, Deutschland*

**Background:**
Robotic-assisted surgery has revolutionized minimally invasive approaches, particularly in colorectal surgery. While single-center studies on colorectal surgeries exist in present literature, including experiences with Senhance® Robotic Systems, comprehensive multi-center studies are yet lacking. This study, conducted through the multi-center TransEnterix European Patient Registry ("TRUST"), aims to assess the safety and feasibility in this context.

**Materials and methods:**
The present study explored procedural times, complications, robotic malfunction and limitations, adverse events and postoperative pain management outcomes for colorectal procedures, including (oncological) sigmoid resection/ left hemicolectomy, right hemicolectomy and oncological rectal surgery. Adult patients were included from two European study centers (Klaipeda University Hospital, Lithuania and Evangelisches Hospital, Wesel, Germany). Patients were screened for eligibility for laparoscopic surgery. Robotic-assisted colorectal surgeries were divided into sigmoid resection, right hemicolectomy and rectal surgery. All procedures were entirely performed with the Senhance® Robotic System. Various papers on procedural details, including our own, are published. In general, three robotic arms were utilized alongside five trocar placements, with a preference for the combination of 12mm and 5mm trocars. The surgical teams conducting surgeries varied across locations. In Wesel, three cockpit surgeons and fourteen assistant surgeons formed 34 distinct constellations, mostly one cockpit and two bedside surgeons. In Klaipeda, the surgical teams comprised three surgeons and seven assistants, leading to three unique surgical constellations. After completing the procedure, “docking time”, “console time”, and “duration of surgery” (in minutes) were documented in a TRUST database. "Docking time" is the period between initiating and concluding the robot's docking to the trocars, while “console time” represents the time the cockpit surgeon operates through the robotic system. At the same time, "duration of surgery" refers to the time from the initial skin incision to the completion of suturing. Perioperative complications and adverse events (AE) were documented in detail when they occurred. Intraoperative complications included AEs involving visceral or vascular injury, cardiopulmonary events, major blood loss (> 500 mL), or the need for blood transfusion. Postoperative complications included AEs that are related to the procedure, such as infections (e.g. wound infection), insufficient anastomosis, sepsis, ileus, bowel obstruction, and venous thromboembolic events and occurred within 30 days postoperative. The recorded data on AEs and complications followed the Clavien Dindo Classifications (Dindo et al. 2004). Severe AEs were identified when readmission, repeated surgery, or death occurred. Additionally, conversions to standard laparoscopy or open surgery were protocolled in the database. Finally, blood loss during the procedure was recorded by estimation and patient medication usage (according to the World Health Organisation) as well as pain scores (on a 0-10 visual pain scale) were collected at first postoperative day and discharge. Each patient provided written informed consent for the robotic procedure. Demographic data such as age, sex, BMI, relevant diseases and previous abdominal surgery were gathered before the procedure. Study Initiation started in October 2017, and the database registry ended in November 2023. We used “statistical software SAS® 9.4 (TS1M6) for Microsoft Windows”. Continuous data were expressed using either the median with interquartile ranges or mean ± standard deviation. Categorical variables were presented in terms of frequencies and percentages.

**Results:**
Data from 355 colorectal surgeries were recorded at Wesel (192 cases) and Klaipeda (163 cases). Sigmoid resection were in 169 cases (47.6%) reported, rectal surgery in 123 patients (34.7%), and right hemicolectomy in 63 cases. The median duration of surgery was 147.2 minutes, and the docking time was reported with a median of 3.4 minutes. The overall pain scores indicated a minimal pain level of 3.1 (±1.7) one day postoperative. By discharge, the pain score had decreased to an average of 1.2 (±1.1).

In 2.9% of cases (10 surgeries) conversion to either an open or laparoscopic approach was performed. The reasons for conversion were limitations due to difficult anatomy, e.g., multiple adhesions, unexpected localization of cancer, T4 tumors requiring multi-visceral resections, or intraoperative bleeding. In few cases limited motion (18.9%, 67 cases) and collision (11.5%, 41 cases) occurred. All adverse events (6.8%, 24 cases) were effectively managed, with none related to the robotic system.

**Conclusion:**
The study provides comprehensive insights into the outcomes and challenges of robotic-assisted colorectal surgeries with the Senhance® Robotic System across multiple centers. The detailed breakdown of our findings enhances our understanding of the nuances and variations in outcomes across different aspects of colorectal surgery. In conclusion, safety and feasibility are demonstrated with the Senhance® Robotic System, and its application across various surgeries in colorectal surgery is approved. This study contributes valuable insights for future research and clinical practice.

**Thoraco-laparoscopic jejunal reconstruction after esophagectomy – a case report of a "modified double tract" without removal of the gastric conduit**
ID: 951

**Kategorie: DGAV - Minimalinvasive und Robotergestützte Chirurgie: Onkologische Chirurgie in MIC und Robotik**

Martin Jazra[1], Nurettin Albayrak[1]

[1]*St. Anna Hospital Herne, General and Abdominal Surgery, Herne, Deutschland*

**Background:**
Severe dysfunction or stenosis of the gastric conduit following esophagectomy is a rare, but very challenging situation. An additional second reconstruction using the jejunum without removal of the conduit can hardly be found in literature.

**Materials and methods:**
We report the case of a 63 year old male patient who underwent totally minimally invasive (combined laparoscopic-thoracoscopic) Ivor-Lewis esophagectomy due to carcinoma of the esophagogastric junction.

Because of a dysfunctional gastric conduit the patient had to undergo re-do laparoscopy with adhesiolysis of the conduit twice. In between he received a gastric stent and suffered from spontaneous stent perforation afterwards with pleural empyema and treatment by re-do thoracoscopy and suture.

When the patient developed a stenosis again oral alimentation was impossible.

We decided to avoid a riskful removal of the conduit with a difficult reconstruction. We performed a second reconstruction with a minimally invasive gastrojejunal Roux-en-y and a thoracoscopically handsewn mediastinal side to end anastomosis to the gastric conduit.

**Results:**
No early complications occurred, the patient was released from hospital within 2 weeks. Oral feeding was possible again, after 4 weeks he reported stable weight and a good general condition. The x-ray with oral contrast showed both pathways.

**Conclusion:**
In very selected cases and after excluding other options we have shown for the first time that a modified double-tract reconstruction using a gastrojejunal Roux-en-y is feasible. It can be performed totally minimally invasive with a thoracoscopic anastomosis. Short term results are promising.

### Reconstruction Techniques and Associated Morbidity in Minimally Invasive Gastrectomy for Cancer - Insights From the GastroBenchmark and GASTRODATA databases

ID: 55

**Kategorie: DGAV - Ösophagus-/Magenchirurgie: Medizintechnische Innovationen und Stellenwert der KI**

Marcel Schneider[1], Jeesun Kim[2], Felix Berlth[3], Yutaka Sugita[4], Peter Grimminger[5], Bas Wijnhoven[6], Hidde Overtoom[6], Ines Gockel[7], René Thieme[7], Ewen Griffiths[8], William Butterworth[8], Henrik Nienhüser[9], Beat Müller[10], Nerma Crnovrsanin[9], Felix Nickel[11], Suzanne Gisbertz[12], Mark I. van Berge Henegouwen[12], Philip Pucher[13], Kashuf Khan[13], Asif Chaudry[14], Pranav Patel[14], Manuel Pera[15], Mariagiulia Dal Cero[15], Carlos Garcia[16], Guillermo Martinez Salinas[16], Paulo Kassab[17], Osvaldo Antônio Prado Castro[17], Enrique Norero[18], Paul Wisniowski[19], Luke Randall Putnam[19], Pietro Maria Lombardi[20], Giovanni Ferrari[20], Rita Gudaityte[21], Almantas Maleckas[21], Leanne Prodehl[22], Antonio Castaldi[23], Michel Prudhomme[23], Hyuk-Joon Lee[2], Takeshi Sano[4], Gian Luca Baiocchi[24], Giovanni De Manzoni[25], Simone Giacopuzzi[25], Maria Bencivenga[25], Riccardo Rosati[26], Francesco Puccetti[26], Domenico D'ugo[27], Gastrodata Consortium[25], Souya Nunobe[4], Han-Kwang Yang[2], Christian Alexander Gutschow[1]

[1]*Department of Surgery & Transplantation, Zürich, Schweiz*, [2]*Department of Surgery, Seoul National University Hospital, Seoul, Südkorea*, [3]*Department of Surgery, University Hospital Tuebingen, Tuebingen, Deutschland*, [4]*Department of Gastroenterological Surgery, , Cancer Institute Hospital of the Japanese Foundation for Cancer Research, Tokyo, Japan*, [5]*Department of General-, Visceral- and Transplant Surgery, University Medical Center Mainz, Mainz, Deutschland*, [6]*Department of Surgery, Erasmus University Medical Center, Rotterdam, Niederlande*, [7]*Department of Visceral, Transplant, Thoracic and Vascular Surgery, University Hospital of Leipzig, Leipzig, Deutschland*, [8]*Department of Upper GI Surgery, Queen Elizabeth Hospital, University Hospitals Birmingham NHS Foundation Trust, Birmingham, Großbritannien*, [9]*Department of General, Visceral and Transplant Surgery, University Hospital Heidelberg, Heidelberg, Deutschland*, [10]*Klinik für Viszeralchirurgie, CLARUNIS Basel, Basel, Schweiz*, [11]*Department of General, Visceral, and Thoracic Surgery, University Medical Center Hamburg-Eppendorf, Hamburg, Deutschland*, [12]*Amsterdam UMC, Amsterdam, Niederlande*, [13]*Department of Surgery, Queen Alexandra Hospital, Portsmouth, Großbritannien*, [14]*The Royal Marsden, London, Großbritannien*, [15]*Section of Gastrointestinal Surgery, Hospital Universitario del Mar, Barcelona, Spanien*, [16]*Hospital San Borja Arriarán, Santiago, Chile*, [17]*Gastroesophageal and Bariatric Surgical Division, Santa Casa of São Paulo Medical School and Hospital, São Paulo, Brazil*, [18]*Esophagogastric Surgery Unit, Hospital Dr Sotero del Rio, Santiago, Chile*, [19]*Division of Upper GI and General Surgery, Keck School of Medicine, Los Angeles, Vereinigte Staaten*, [20]*Division of Minimally Invasive Surgical Oncology, Niguarda Cancer Center, Milan, Italien*, [21]*Department of Surgery, Hospital of Lithuanian University of Health Sciences, Kaunas , Lithuania*, [22]*Department of Surgery, Charlotte Maxeke Johannesburg Academic Hospital, Johannesburg, South Africa*, [23]*Service de Chirurgie Digestive et Cancérologie Digestive, Hôpital Universitaire Carémeau, Nimes, Frankreich*, [24]*Department of Surgery, University Hospital of Brescia, Brescia, Italien*, [25]*Department of Surgery, University Hospital of Verona, Verona, Italien*, [26]*IRCCS San Raffaele Scientific Institute, Milan, Italien*, [27]*FONDAZIONE POLICLINICO UNIVERSITARIO GEMELLI-IRCCS, Rom, Italien*

**Background:**
Various anastomotic and reconstruction techniques are used for minimally invasive total (miTG) and distal gastrectomy (miDG). Their effects on postoperative morbidity have not been extensively studied.

**Materials and methods:**
MiTG and miDG patients were selected from 9356 oncological gastrectomies performed in 2017–2021 in 44 centers. Endpoints included anastomotic leakage (AL) rate and post-operative morbidity tested by multivariable analysis.

**Results:**
Three major anastomotic techniques [circular stapled (CS); linear stapled (LS); and hand sewn (HS)], and 3 major bowel reconstruction types [Roux (RX); Billroth I (BI); Billroth II (BII)] were identified in miTG (n = 878) and miDG (n = 3334). Post-operative complications, including AL (5.2% vs 1.1%), overall (28.7% vs 16.3%), and major morbidity (15.7% vs 8.2%), as well as 90-day mortality (1.6% vs 0.5%) were higher after miTG compared with miDG. After miTG, the AL rate was higher after CS (4.3%) and HS (7.9%) compared with LS (3.4%). Similarly, major complications (LS: 9.7%, CS: 16.2%, and HS:

12.7%) were lowest after LS. Multivariate analysis confirmed anastomotic technique as a predictive factor for AL, overall, and major complications. In miDG, AL rate (BI: 1.4%, BII 0.8%, and RX 1.2%), overall (BI: 14.5%, BII: 15.0%, and RX: 18.7%), and major morbidity (BI: 7.9%, BII: 9.1%, and RX: 7.2%), and mortality (BI: 0%, BII: 0.1%, and RY: 1.1%%) were not affected by bowel reconstruction.

**Conclusion:**
In oncologically suitable situations, miDG should be preferred to miTG, as postoperative morbidity is significantly lower. LS should be a preferred anastomotic technique for miTG in Western Centers. Conversely, bowel reconstruction in DG may be chosen according to the surgeon’s preference.

**Esophageal perforations – analysis of etiology, management and outcome – a 12 years experience at a tertiary university medical center**
ID: 615

**Kategorie: DGAV - Ösophagus-/Magenchirurgie: Medizintechnische Innovationen und Stellenwert der KI**

Max Alexander Kölling[1], Nora Friedrich[1], Diana Voll[1], Polina Rösel[1], Thorsten Perl[1], Volker Ellenrieder[2], Maximilian von Heesen[1], Michael Ghadimi[1], Johanna Reinecke[2], Lena-Christin Conradi[1]

[1]*Universitätsmedizin Göttingen, Klinik für Allgemein-, Viszeral- und Kinderchirurgie, Göttingen, Deutschland*, [2]*Universitätsmedizin Göttingen, Klinik für Gastroenterologie, gastrointestinale Onkologie und Endokrinologie, Göttingen, Deutschland*

**Background:**
Esophageal perforation is a rare, but life-threatening condition with high morbidity and mortality, particularly when diagnosis and treatment are delayed. Most commonly esophageal perforation has an iatrogenic origin with significant contribution of transesophageal echocardiography as well as endoscopic interventions and surgical procedures. Despite advances in medical technology, especially over the last decade, the management of esophageal perforations remains challenging due to its varied etiology and complex clinical presentation. To contribute to a better understanding of its management and offer further insight on the choice of treatment we present a retrospective, monocentric analysis of twelve years of experience in diagnostics and treatment of esophageal perforations at German university hospital.

**Materials and methods:**
We conducted a retrospective analysis of 77 cases of esophageal perforation treated at the University Medical Center Göttingen between 2012 and 2024. Patient data were collected from electronic and analogous records, focusing on perforation etiology, diagnostic challenges, treatment strategies, and outcomes. The Pittsburgh Severity Score (PSS) was used to assess perforation severity. Kaplan-Meier survival curves were generated to evaluate survival analyses. Statistical analysis was provided via log-rank and Gehan-Breslow-Wilcoxon test.

**Results:**
In our cohort we observed a slight majority of male patients with 61% (n=47) to 39% (n=30) female patients and the mean age being 66,5 years (18-93). Iatrogenic perforations with 57,1% (n=44) were the leading cause, followed by Boerhaave's syndrome with 24,7% (n=19) and foreign body ingestion with 11,7% (n=9). Tumor associated perforations occurred in 5,2% (n=4) of the cases and a singular case of traumatic perforation was reported. Perforations of the lower esophageal third were most common (75,3%; n=58), followed by perforations of the mid esophagus (13%; n=10) and the upper esophagus (11,7%; n=9). Multidisciplinary management, encompassing both surgical and non-operative approaches, played a pivotal role in influencing outcomes and evolved over the years. Endoscopic therapy dominated the field of primary treatment with 48,1% (n=37) over primary surgery (32,5%; n=25) and conservative treatment (19,5%; n=15). EndoVAC therapy emerged as a key non-surgical treatment with 29,9% (n=23) patients treated primarily or secondary to a previous treatment. It sparked a rapid increase of primary endoscopic management and proved to significantly (p=0,034) improve survival after its implementation in May 2017 compared to the time before. After all surgical management still played a major role with eventually 62,3% (n=48) of patients undergoing surgical management as either primary or secondary treatment, often in combination with endoscopic management (23,4%; n=18). The severity of esophageal perforations was underlined by data collected in the ICU. A total of 60 (77,9%) patients were admitted to an ICU during treatment with 37 of them in need of invasive ventilation. Risk stratification via PSS showed a majority of patients with severe, high-risk perforations scoring 6 or more points (n=34; 44%). Intermediate risk (3-5 pts.) was assessed in 29 cases (38%), whereas low-risk patients formed a minority of 14(18%). Patients with higher PSS scores underwent surgical treatment more often, and elevated PSS was associated with increased mortality.

**Conclusion:**
Our study underscores the importance of early diagnosis and especially multidisciplinary approach in managing esophageal perforations. Our findings were consistent with previous publications while providing a rather broad approach and comparably large cohort regarding this rare condition. The shift towards increased use of non-operative treatments, especially EndoVAC, has led to improved patient outcomes and further development is to be observed in the coming years. Special attention should be given to the application of VAC-stent devices, currently being established in the management of anastomotic insufficiency after esophageal surgery. After all surgical intervention remains essential in severe cases, particularly those involving cardiopulmonary complications and instability or large esophageal defects. Future studies should focus on prospective and multicentric approaches possibly using standardized databases and automated analysis, further simplifying data collection and hopefully allowing the deduction of treatment protocols backed with more and even better evidence to further optimize outcomes of this critical condition.

### Adherence to S3 Guideline for MRI Sequencing in Soft Tissue Sarcoma Diagnosis: An Evaluation of Imaging Practices and Compliance

ID: 125

**Kategorie: DGAV - Perioperative Medizin: Evidenzbasiertes Handeln**

Madelaine Hettler[1], Josephine Kirschstein[1], Isabelle Ayx[2], Melissa Harbrücker[1], Franka Menge[1], Matthias Frölich[2], Dominik Nörenberg[2], Jens Jakob[1]

[1]*Universitätsmedizin Mannheim, Chirurgische Klinik, Sarkomzentrum, Mannheim, Deutschland*,
[2]*Universitätsmedizin Mannheim, Klinik für Radiologie und Nuklearmedizin, Mannheim, Deutschland*

**Background:**

A correct diagnosis is essential in planning treatment and assessing the prognosis when a soft tissue sarcoma (STS) is suspected. Imaging is crucial for tumor characterization and surgical planning. Additionally, the functional imaging (e.g. diffusion weight imaging, ADC) may contribute to the correct assessment of grading. The German S3 guideline “Adult Soft Tissue Sarcoma” recommends contrast-enhanced magnetic resonance imaging (MRI) when a STS is suspected. At least five defined sequences and functional diffusion imaging with Apparent Diffusion Coefficient (ADC) sequence are recommended. The study aims to evaluate the consistency between the MRI sequences recommended in the S3 guidelines and those performed at the time of initial sarcoma diagnosis.

**Materials and methods:**

This study is based on a cohort of sarcoma patients treated at the Sarcoma Centre, University Medical Centre Mannheim (UMM). All MRIs were analyzed with regard to the sequences performed. The MRI sequences performed were compared with those recommended in the German S3 guideline. The imaging data was analyzed by an MD student (JK), a surgical resident (MH) and reviewed by a senior radiologist (IA).

**Results:**

We evaluated imaging studies in a total of 64 patients with soft tissue sarcoma of the extremities and trunk. Of these MRIs, 62 were performed at external radiology centers, while 2 were done at UMM. In 43/64 (67 %) of the cases, the referral form for radiology explicitly indicated the presence of a suspected mass. Pre-treatment contrast-enhanced MRI was available for 39/43 (91 %) with suspected tumor and for 9/21 (43 %) with incidental diagnosis. Diffusion-weighted imaging, a potentially predictive MRI sequence, was available for 12/64 (19 %) patients. Among these 10/43 (23 %) patients had a suspected tumor and 2/21 (10 %) patients had an incidental diagnosis. In more than 80 % of all cases, not all MRI sequences recommended in the guideline text were performed.

**Conclusion:**

The choice of the MRI protocol is primarily based on the specific clinical question. The analysis reveals that a significant proportion of the analyzed MRI examinations did not include the guideline-recommended sequences. Notably, diffusion imaging was frequently omitted. Possible explanations include a lack of familiarity with the guidelines, outpatient billing practices, the radiologist’s preference for alternative sequences they may consider more suitable or a different initial suspected diagnosis as an indication for the MRI. In the situation of an incidental diagnosis, an adjustment of the MRI protocol during the examination, in accordance with the guidelines, would be necessary. Prospective evaluation studies should be considered, particularly to assess tumor margins for resection planning and to improve functional imaging grading.

**Outcome after prehabilitation prior to pancreatoduodenectomy: A retrospektive, single-center study**
ID: 248

**Kategorie: DGAV - Perioperative Medizin: Evidenzbasiertes Handeln**

Philipp Scherber[1], Jana Bauer[1], Sebastian Holländer[1], Gereon Gäbelein[1], Peter Jacob[1], Matthias Glanemann[1]

[1]*Universitätsklinikum des Saarlandes, Klinik für Allgemeine Chirurgie, Viszeral-, Gefäß und Kinderchirurgie, 66424, Homburg, Deutschland*

**Background:**

An optimized preparation in order to improve patients´ health prior to surgery, known as prehabilitation, is becoming an increasingly integral part of the multimodal treatment of oncological patients. However, the current body of research is highly heterogeneous due to inconsistent patient populations and variations in the type and duration of interventions. Data on prehabilitation is particularly scarce in pancreatic cancer, where neoadjuvant treatment approaches are less established, and a definitive surgical intervention should follow the diagnosis as promptly as possible.

The aim of the present study was, on the one hand, to determine the acceptance and dropout rate of the intervention, and on the other hand, to examine the effect of prehabilitation on the peri- and postoperative outcomes after pancreatoduodenectomy in comparison to randomly selected patients who had undergone surgery by the same surgeons and had been treated according to the same peri- and postoperative standards in the previous year.

**Materials and methods:**

Prehabilitation was introduced for patients scheduled for pancreatic head resection at the Department of General Surgery, Visceral-, Vascular-, and Pediatric Surgery at the Saarland University Medical Center on January 1, 2023. After providing oral and written information during the initial consultation in the hepato-pancreato-biliary outpatient clinic, patients who agreed and had undergone a nutritional assessment were given a logbook for information and documentation purposes. This logbook contained strength-, endurance-, and breathing exercises to be performed, as well as a nutrition plan. During the intervention, patients were contacted by phone. The initially planned surgery date was not to be postponed due to the program; a prehabilitation period of less than 8 days was considered an exclusion criterion.

**Results:**

Between January 2023 and June 2024, a total of 102 pancreatoduodenectomies were performed at the authors´ institution. After excluding 44 patients (43.1%) due to lack of interest, transfer for surgery from an external hospital, or a short interval between initial consultation and surgery, prehabilitation was initiated in 58 patients (56.9%). Out of these, 30 patients (27.5%) who had completed prehabilitation and subsequently had undergone pancreatic head resection, were included in the study (“P”). They were compared to 30 randomly selected control patients (“C”) who underwent surgery in the previous year. The median duration of prehabilitation were 15 days.

The two groups did not differ in terms of demographic parameters or the type and severity of pre-existing conditions (ASA class). Pylorus-preserving pancreaticoduodenectomy was the predominant surgical procedure in both groups (P: 28 [93.3%]; C: 27 [93.3%]), and the pancreatojejunostomy was the dominant type of anastomosis (P: 26 [86.7%]; C: 23 [76.7%]). There were no differences between the groups regarding duration of surgery, blood loss, or transfusion rates. Postoperatively, the overall complication rate was significantly lower in patients who underwent prehabilitation: 50% vs. 76.7%, $p = 0.032$. Differences between the groups for the particular surgical and non-surgical complications did not reach the 5% significance level. However, a trend towards a lower rate of severe complications according to Clavien-Dindo Class 2 - 4 was observed in the group of patients with prehabilitation (P: 36,6% ; C: 66,7%).

Postoperatively, the median ICU stay for both groups was one day. Patients in the prehabilitation group were discharged from the hospital 1.5 days earlier on average (13.5 vs. 15 days, not significant). Intrahospital mortality was 3.3% (one patient in each group). During the 90-day follow-up, the readmission rate was 10.3% in both groups (three patients each).

**Conclusion:**

After pancreatoduodenectomy, patients may benefit from an optimized preparation prior to surgery. The present study provides indications of a lower postoperative complication rate and faster recovery, leading to a shorter hospital stay. However, studies involving larger populations and high-quality research are needed to confirm this trend.

**The role of acquired Factor XIII deficiency in the perioperative management of patients undergoing (acute) abdominal surgery and its effect on postoperative complications**
ID: 290

**Kategorie: DGAV - Perioperative Medizin: Evidenzbasiertes Handeln**

Jorieke Nijhuis[1 2], Guido Peterschulte[1], Claus Ferdinand Eisenberger[1]

[1]*Kliniken der Stadt Köln gGmbH, Köln, Deutschland*, [2]*Universitair Medisch Centrum Groningen, Groningen, Niederlande*

**Background:**
Fibrin stabilizing factor (Factor XIII) is recognized for its function in the coagulation cascade. Factor XIII also significantly contributes to wound healing, tissue repair and immune defense as well. A deficiency of Factor XIII could be acquired through, for example, major surgery, massive bleeding, or sepsis.
Patients undergoing (acute) abdominal surgery are at risk for developing acquired Factor XIII deficiency, therefore potentially increasing the probability of postoperative complications.
The objective of this study was to evaluate the prevalence of acquired Factor XIII deficiency in patients after (acute) abdominal surgery and the incidence of postoperative complications. Ultimately to assess perioperative risk factors to improve preventive strategies in the perioperative management.

**Materials and methods:**
Data of all patients who received Factor XIII substitution after (acute) abdominal surgery between February 2021 and February 2024 at Kliniken Köln was collected retrospectively. Patient, perioperative and complication characteristics, as well as Factor XIII substitution data were acquired. Complications were analyzed and categorized per the Clavien-Dindo classification.

**Results:**
A total of ninety-three patients who received Factor XIII substitution after (acute) abdominal surgery were identified, with a mean age of 71.1 years (±13.9 SD) and male predominance (57.3%). Reasons for surgery consisted of i.e., organ perforation, ileus, and malignancy.
In all patients Factor XIII level at time of substitution was below the threshold of 70.0%.
Factor XIII levels at time of substitution were on average 41.7% (±11.9 SD). In 57.9% of all patients a substitution of 2500IE Factor XIII was administered. In eighty-two patients the Factor XIII level was initially <50%, in fifty-eight patients <40%, in twenty-eight patients <30%.
Postoperative complications per the Clavien-Dindo classification were divided into minor (grades I and II) and major complications (grades III or higher). In the preliminary results, the incidence of major complications was 67.3%. The incidence of wound healing disorder was 39.8%, anastomotic insufficiency occurred in 19.4%.
In 64.7% of patients with major complications and 25.0% of patients with minor complications, more than 1250IE Factor XIII substitution was administered.

**Conclusion:**
Acquired Factor XIII deficiency frequently occurs in patients after (acute) abdominal surgery.
Compared to the minor complications group, the major complications group required more substitution of Factor XIII to reach sufficient levels.
Further determination of risk factors and their predictive value is warranted to optimize the perioperative management in patients with (acute) abdominal surgery.

### ERAS for gastric resections – a pilot project

ID: 625

**Kategorie: DGAV - Perioperative Medizin: Evidenzbasiertes Handeln**

Maria-Margarete Mende[1], Judith Christina Sporn[1], Steffi Scholz[1], Franziska Peters[1], Jörg-Peter Ritz[1]

[1]*Klinik für Allgemein- und Viszeralchirurgie, Helios Kliniken Schwerin, Schwerin, Deutschland*

**Background:**

Enhanced Recovery After Surgery (ERAS) protocols have shown to improve outcomes and hospital length of stay for patients undergoing surgery. The ERAS program includes a variety of aspects including patient education, nutritional optimization and standardization of preoperative, perioperative and postoperative care. These efforts aim to lead to faster recovery, fewer complications and a shorter length of stay. The ERAS program has been well established for colorectal procedures in Germany. However, there is only limited experience with the use of an ERAS protocol in upper GI surgery. Here, we present our experience with our recently established ERAS protocol following gastrectomies.

**Materials and methods:**

This is an analysis of prospectively collected data of all patients who underwent gastric resections on the ERAS protocol at Helios Kliniken Schwerin from 10/2023 to 08/2024. The ERAS protocol for gastric resections was based on our established ERAS protocols for colorectal and pancreatic surgery. Bariatric cases (sleeve gastrectomy or gastric bypass procedures) as well as esophagectomies were excluded from the analysis. Data were extracted from our ERAS database.

**Results:**

17 patients were included in the analysis. 9 of 17 patients (53.0%) underwent total gastrectomies, 6 of 17 patients (35.3 %) underwent subtotal gastrectomies and 2 of 17 patients (11.8 %) gastric wedge resections. 11 of 17 cases (64.7%) were performed minimally invasively (robotically or laparoscopically), 6 of 17 cases (35.3%) were open cases. All patients received preoperative education and were screened according to the ERAS protocol. 3 of 17 patients (17.6%) were at risk of malnutrition, 2 of 17 (11.8%) received preoperative parenteral nutrition, 1 patient (5.9%) received high protein oral nutritional supplements. Preoperative carbohydrate loading was administered in 14 of 17 patients (82.3 %). An intraabdominal drain was left in only 3 of 17 patients (17.6 %) at the end of surgery. The intraoperatively placed nasogastric tube was removed before extubation in all patients. TIVA technique and prophylaxis of PONV were used in all cases. The mean amount of intravenous fluids on the day of operation was 3450ml (ranging from 1100 ml to 10050 ml). IV fluids were stopped on postoperative day 1 in all patients. Patients were mobilized out of bed for 78 minutes on postoperative day 1, for 138 minutes on postoperative day 2, and for 168 minutes on postoperative day 3. Median hospital stay was 11 days (ranging from 4 days to 11 days). All patients followed up at the requesesd time interval, 1 patient (5.9%) had to be readmitted to the hospital within 30 days for reasons not related to the surgery. The adherence of all patients to the ERAS protocol was 71.4%.

**Conclusion:**

Our pilot project showed that the establishment of an ERAS protocol for gastrectomy patients was feasible, but associated with specific challenges for the care team. Early postoperative oral food intake was not tolerated by all patients and impossible when patients had to be treated with endoluminal vacuum therapy in the case of anastomotic leaks. Mobilization in our ERAS gastrectomy group was delayed compared to the colorectal group which might be due to our institutional policy of admitting most gastrectomy patients to the ICU after surgery and to the limitations of physical therapy in the ICU setting. Peridural analgesia is well established as an efficient perioperative pain management tool at our institution with reluctance to give it up. However, the use of TAP blocks has gradually increased over the months. In summary, our data show the potential of ERAS protocols for the perioperative management of gastrectomy patients which will certainly influence and reshape our treatment strategies in the future.

**Data**

**Preoperative data**

| | |
|---|---|
| Nutrional screening | 94 % |
| Risk of malnutrition | 17.6 % |
| Parenteral nutrition | 11.8 % |
| Enteral supplements | 5.9 % |
| Iron deficiency anemia | 5.9 % |
| Carboloading | 82.3 % |
| Patient training preoperative | 100 % |

**Perioperative Data**

| | |
|---|---|
| Nasogastric tube removed before extubation | 100 % |
| Enteral feeding catheter placed | 11.8 % |
| Drain | 17.6 % |
| IV fluids | 3450 ml |

**Postoperativa Data**

| | |
|---|---|
| End of IV fluids and IV medication | Postoperative day (POD) 1 |
| Length of mobilization POD 1 | 78 minutes |
| Length of mobilization POD 2 | 138 minutes |
| Length of mobilization POD 3 | 168 minutes |
| Length of stay | 11 d (median) |
| Adherence | 71.4 % |

### Tranexamic acid for reduction of perioperative blood loss and transfusion requirements in major abdominal surgery: systematic review and meta-analysis

ID: 633

**Kategorie: DGAV - Perioperative Medizin: Evidenzbasiertes Handeln**

Ulrich Ronellenfitsch[1], Ann-Cathrin Schweitzer[1], Artur Rebelo[1], Jörg Kleeff[1]

[1]*Universitätsmedizin Halle, Universitätsklinik und Poliklinik für Viszerale, Gefäß- und Endokrine Chirurgie, Halle (Saale), Deutschland*

**Background:**

Blood loss and transfusion requirements during major abdominal operations are two key factors in determining postoperative outcomes. The antifibrinolytic tranexamic acid (TXA) has been known to reduce hemorrhage in many surgical situations. However, the evidence regarding abdominal surgery is heterogeneous.

**Materials and methods:**

We conducted a systematic review with meta-analysis to comprehensively assess the effect of tranexamic acid on blood loss and transfusion requirements in major abdominal surgery. An electronic database search of PubMed and Cochrane Library was conducted, in order to identify all studies published between database inception and July 2021. Two independent reviewers screened trials and included all studies in which TXA had been used in major abdominal surgery (esophagectomy, gastrectomy, pancreatectomy, liver and colorectal resections). There were no limitations regarding study design, co-morbidities of patients or surgical access. Abstracts and full texts had to be available in English or German. The primary outcomes assessed were perioperative blood loss and blood transfusion requirements. Secondary outcomes were mortality, thromboembolic and other adverse events, operating time, hospital and ICU length of stay. Data were analyzed using the RevMan 5.4 software applying random-effects models. For each outcome, risk ratio (RR) and mean difference (MD) or standardized mean difference (SMD) with 95% confidence intervals (CIs) were calculated. TXA was compared with placebo or no intervention and with other antifibrinolytic agents, specifically Aprotinin and EACA. The quality of the evidence was assessed using GRADE. The risk of bias was assessed with the dedicated tool of the Cochrane Collaboration..

**Results:**

Nineteen trials involving 3.087 patients (18 to 734 patients) were included. The number of patients requiring transfusions (MD 0.67; 95% CI 0.48 to 0.92; 10 trials, 1295 patients, moderate quality evidence) as well as the required units (MD -1.73; 95% CI -3.05 to -0.41; 6 trials, 1026 patients, moderate quality evidence) and FFP (MD -1.90; 95% CI -3.58 to -0.22; 6 trials, 1026 patients, moderate quality evidence) were significantly lower in the tranexamic acid versus the control group. No significant differences were found in the outcomes perioperative blood loss, platelet and cryoprecipitate transfusion, thromboembolic events, serious adverse events, seizures, mortality, operating time, length of ICU or hospital stay. Sensitivity analyses did not show any significant differences between the trials of high and low risk of bias. For type of surgery no significant differences for any of the primary outcomes existed between subgroups. The comparison of TXA with Aprotinin showed significantly higher cryoprecipitate transfusion requirements in the TXA group (MD 21.00 units; 95% CI 13.93 to 28.07; 1 trial, 39 patients, low quality evidence) and a significantly shorter length of hospital stay (MD -4.50 days; 95% CI -8.12 to -0.88; 1 trial, 39 patients, low quality evidence). Treatment with EACA led to significantly higher RBC (MD -3.07, 95% CI -4.84 to -1.30; 1 trial, 164 patients, high quality evidence), FFP (MD -2.88; 95% CI -4.77 to -0.99; 1 trial, 164 patients, high quality evidence) and cryoprecipitate (MD -4.01; 95% CI -6.68 to -1.34; 1 trial, 164 patients, high quality evidence) transfusion requirements compared to TXA. No other statistically significant differences occurred in comparison with any of the other antifibrinolytic agents.

**Conclusion:**

The results of this meta-analysis based on a systematic review of the literature support the administration of TXA in major abdominal surgery to reduce transfusion requirements. The results do not show an increase of thromboembolic events or other serious adverse events. However, given the moderate quality of evidence, these findings need to be further confirmed in adequately powered, well-designed randomized controlled trials.

### Robotic-assisted distal pancreatectomy is associated with significant reduction of postoperative acute pain

ID: 667

**Kategorie: DGAV - Perioperative Medizin: Evidenzbasiertes Handeln**

Jens P. Hölzen[1] , Amelie F. Kruse[1], Shadi Katou[1], Haluk Morgül[1], Mazen A. Juratli[1], Andreas Pascher[1], Benjamin Strücker[1], Andreas Andreou[1]

[1]*Universitätsklinikum Münster, Klinik für Allgemein-, Viszeral- und. Transplantationschirurgie, Münster, Deutschland*

**Background:**
Minimal invasive techniques have been increasingly performed for the treatment of malignant and benign lesions of the pancreatic tail. In this study, we aimed to evaluate the differences between open and robotic surgical approach for distal pancreatectomy (DP) in terms of postoperative pain and analgesic consumption.

**Materials and methods:**
Clinicopathological data of patients who underwent DP from March 2012 to May 2023 were evaluated. The study compared robotic distal pancreatomies (RPD) with open distal pancreatomies (OPD) with regards to total postoperative morphine consumption, which represents a reliable indirect measurement of pain.

**Results:**
During the study period, 78 DP were performed, including, 24 RDP and 54 OPD. Multivisceral resections were excluded from the analysis. Patients who underwent RPD required significantly less opioid analgesics after surgery (113.60; 0,00- 516,20 mg morphine equivalents; median; minimum-maximum) compared to those in the ODP group (253.75; 15,00- 3519,45 mg; $p<0.001$). When adjusted for patient weight, the morphine equivalent dose also showed a significant difference between the RDP (1.51; 0,00- 6,53 mg/kg) and ODP (3.19; 0,24- 62,85 mg/kg; $p=0.004$) group.

**Conclusion:**
This study demonstrated that patients who underwent RDP hat significantly less pain after surgery and thus required lower amounts of opioid analgesics.

**Between practice and evidence: The pre-, intra- and postoperative treatment of elective laparoscopic cholecystectomy - A Germany-wide survey**
ID: 771

**Kategorie: DGAV - Perioperative Medizin: Evidenzbasiertes Handeln**

Jonathan Storde[1], Dirk R. Bulian[2]

[1]*Universität Witten/Herdecke, Witten, Deutschland*, [2]*Klinik für Viszeral-, Tumor-, Transplantations- und Gefäßchirurgie, Kliniken der Stadt Köln, Zentrum für interdisziplinäre Viszeralmedizin (ZIV), Köln, Deutschland*

**Background:**
Cholecystectomy is one of the most frequently performed operations in Germany. The optimization and standardization of certain methods are therefore highly relevant, particularly in order to improve patient safety and minimize postoperative complications.
The aim of this nationwide survey was to determine the current status of pre-, intra- and postoperative procedures in laparoscopic cholecystectomy and thus to ascertain the penetration of scientific evidence in everyday clinical practice.

**Materials and methods:**
Between June and September 2023, 1027 (visceral) surgery hospitals in Germany were contacted by email via the German Society for General and Visceral Surgery and asked to take part in an online survey. The survey asked for general information from the departments and various points about the standard preoperative, intraoperative and postoperative procedure for laparoscopic cholecystectomy.

**Results:**
A total of 399 data sets were complete and analyzable (response rate 38.6%). 39.3% regularly carry out perioperative antibiotic prophylaxis before an elective laparoscopic cholecystectomy. In 60.2% of cases, open initial access is the standard for the creation of the capnoperitoneum, in 25.1% the use of the Veres cannula. In practice, the ultrasonic hook is only used in 2.3% of cases when dissecting the D. cysticus and A. cystica. Only 4.3% regularly insert a drainage tube during laparoscopic cholecystectomy for uncomplicated symptomatic cholecystolithiasis. 24.1% of respondents perform a subcutaneous infiltration at the end of the operation and 12% before the stab incision, one third of them in addition to an intraperitoneal instillation. 2.3% perform intraperitoneal instillation only. 1.8% have a transversus abdominis plane block applied through the anesthesia, while 58.6% do not administer any local anesthetic at all in addition to systemic analgesia.

**Conclusion:**
t has been shown that most surgical hospitals in Germany follow the evidence-based recommendation not to insert a drain during elective laparoscopic cholecystectomy.

**Excisional biopsy of perforated gastric ulcer: mandatory or potentiallyharmful?**
ID: 886

**Kategorie: DGAV - Perioperative Medizin: Evidenzbasiertes Handeln**

Faruk Koca[1], Christine Koch[2], Falko Schulze[3], Ursula Pession[1], Wolf Otto Bechstein[1], Patrizia Malkomes[1]

[1]*Goethe-Universität Frankfurt, Klinik für Allgemein-, Viszeral-, Transplantations- und Thoraxchirurgie, Frankfurt am Main, Deutschland*, [2]*Goethe-Universität Frankfurt, Gastroenterologie und Hepatologie, Pneumologie und Allergologie, Endokrinologie und Diabetologie sowie Ernährungsmedizin, Frankfurt am Main, Deutschland*, [3]*Goethe-Universität Frankfurt, Dr. Senckenbergische Institut für Pathologie, Frankfurt am Main, Deutschland*

**Background:**
This study aimed to evaluate the morbidity associated with excisional biopsy in patients with spontaneous gastric perforation.

**Materials and methods:**
A retrospective, single-center, observational study was performed. All consecutive patients with spontaneous gastric perforation who underwent surgical therapy were included. This study has been approved by the local ethics committee of the Faculty of Medicine at the Goethe University Frankfurt, Germany (reference number: 2024−1695). Outcomes were assessed concerning the performance of excisional biopsy.

**Results:**
A total of 135 adult patients were enrolled. Of these, 110 (81.5%) patients underwent excisional biopsy, while 17 (12.6%) did not. The remaining eight (5.9%) patients who underwent gastric resection were excluded from the analysis. Patients undergoing excisional biopsy developed significantly higher rates of postoperative complications ($p$=0.007) and experienced more severe complications according to the Clavien-Dindo classification, particularly type III and above ($p$=0.017). However, no significant differences were observed regarding in-hospital mortality, reoperation, suture dehiscence, or length of hospital stay.

**Conclusion:**
Excisional biopsy for gastric perforation has been shown to be associated with increased morbidity. Surgical closure followed by early endoscopic biopsy may be a superior approach for gastric perforation management to rule out malignancy.

**Impact of positive peritoneal Candida culture on outcome of patients with perforated peptic ulcer**
ID: 901

**Kategorie: DGAV - Perioperative Medizin: Evidenzbasiertes Handeln**

Faruk Koca[1], Ursula Pession[1], Armin Wiegering[1], Niels Matthes[1]

[1]*Goethe-Universität Frankfurt, Klinik für Allgemein-, Viszeral-, Transplantations- und Thoraxchirurgie, Frankfurt am Main, Deutschland*

**Background:**
This study aimed to evaluate the outcome of microbiology of peritoneal swab, especially positive Candida culture in patients with perforated peptic ulcer.

**Materials and methods:**
A retrospective, single-center, observational study was performed. It was approved by the local ethics committee of the Faculty of Medicine at the Goethe University Frankfurt, Germany (reference number: 2024-1695). All adult patients with perforated peptic ulcer, who underwent surgical therapy at the University Hospital Frankfurt from January 2002 to February 2024, were included. Outcomes were analyzed in relation to microbiology of peritoneal swab.

**Results:**
A total of 187 adult patients were included. 155 (82.9%) patients had an intraoperative peritoneal swab culture. 32 (17.1%) patients without a culture were excluded from the analysis. Patients with a positive Candida culture had significantly higher in-hospital mortality ($p<0.001$), postoperative complications ($p<0.001$), especially type III and higher Clavien-Dindo complications ($p<0.001$), suture dehiscence rates ($p=0.003$), and length of hospital stay ($p=0.002$), while a positive culture for Streptococcus did not affect any of these outcome parameters.

**Conclusion:**
Antifungal therapy should be considered in critical cases with perforated peptic ulcer because a positive Candida culture has been associated with increased mortality and morbidity.

**AI-Driven Intelligent Document Management for Organizing Outside Medical Records: A Field Study in Pancreatic Surgery Second Opinion Requests**
ID: 916

**Kategorie: DGAV - Perioperative Medizin: Evidenzbasiertes Handeln**

Thomas M. Pausch[1 2], Matthias Ganzinger[2], Pascal Fuchs[1 2], Cornelia Lyu[1 2], Yvonne Seelig[2], Rosa Klotz[1], Martin Loos[1], Martin Dugas[2]

[1]*Klinik für Allgemein-, Viszeral- und Transplantationschirurgie, Universitätsklinikum Heidelberg, Heidelberg, Deutschland*, [2]*Institut für Medizinische Informatik (IMI), Universitätsklinikum Heidelberg, Heidelberg, Deutschland*

**Background:**
Patients seeking a second medical opinion often bring a variety of outside medical records, which can be challenging to organize and interpret efficiently. The process of manually sorting and reading through these records is time-consuming and prone to errors. To address this issue, an AI-driven intelligent document management system was developed, capable of automatically organizing outside medical records brought by pancreatic surgery patients seeking a second opinion at the European Pancreas Centre (EPC) at Heidelberg University Hospital. This study aims to evaluate the effectiveness of the AI-enhanced document management system in improving clinical efficiency and decision-making compared to using raw, unprocessed external medical records.

**Materials and methods:**
A prototype intelligent document management system was conceptualized and developed as part of a medical informatics thesis. The system's AI capabilities include classifying document types, recognizing document creation dates, and using this information to organize records into a navigable, ordered overview for each patient. The system processes images of documents using a combination of image preprocessing, text recognition, text vectorization, and classification methods, ultimately generating a searchable, chronologically organized PDF. The classification process employs Bag-of-Words (BOW) and Logistic Regression, which demonstrated strong performance comparable to advanced language models like medBERT.de. Further enhancement in classification accuracy was achieved through manual text postprocessing. Document creation dates were extracted using AI-based pattern recognition methods, including Regular Expressions. These AI tools provided the foundation for a reliable and automated way to sort and organize patient records. Image preprocessing techniques such as increased brightness, contrast adjustments, and noise reduction were implemented to enhance text recognition quality.
Building on this prototype, a clinical study is currently being conducted to compare clinical workflows involving external records in their original form (Group A) versus digital preprocessed records managed by the AI-driven intelligent document system (Group B). Five surgical physicians from the EPC at Heidelberg University Hospital participated, including assistants, specialists, and senior consultants. They were tasked with assessing external records to identify surgical indications, determine missing information, and make decisions regarding surgical eligibility. Each task was repeated twice for consistency. Metrics include task completion time, task accuracy, and user preferences. Physicians in Group B also rated the utility of system features such as summary cover sheets, chronological sorting, an interactive table of contents, and text search. Preferences for future features, such as highlighting and decision recommendations, were also gathered.

**Results:**
The quantitative analysis of the study is currently ongoing, and detailed results will be presented at the time of the congress. Preliminary findings indicate that the AI-driven intelligent document management system has the potential to significantly reduce the time required to assess external medical records and improve the accuracy of information extraction. Physicians working with digitally preprocessed records (Group B) reported higher satisfaction levels, particularly with features such as chronological sorting and text search. There was also a strong preference for future developments, including content summaries and decision recommendations. The AI-based classification and preprocessing approaches played a key role in ensuring that critical information was readily accessible, which facilitated more efficient clinical decision-making.

**Conclusion:**
The AI-driven intelligent document management system shows promise in enhancing efficiency and quality in clinical workflows for pancreatic surgery second opinions. Automatically classifying, preprocessing, and organizing records saves time and improves decision-making by making key information accessible. AI technologies, like natural language processing and pattern recognition, have been crucial to the system's success. Initial feedback from physicians indicates a positive impact, with strong support for further feature development. Adapting these models to meet medical standards and address ethical and data protection issues will be vital for broader adoption.

**Dual-track oncologic-metabolic treatment in patients with rectal carcinoma and severe obesity – a case series report of the Oncologic-Metabolic Board, Comprehensive Cancer Center Freiburg**
ID: 952

**Kategorie: DGAV - Perioperative Medizin: Evidenzbasiertes Handeln**

Gabriel Seifert[1], Stephan Herrmann[1], Lars Schmitt[1], Cornelius Struck[2], Antonia Pahl[2], Maria Koukou[2], Christine Greil[2], Hannes P. Neeff[1], Stefan Fichtner-Feigl[1]

[1]*Universitätsklinikum Freiburg, Allgemein- und Viszeralchirurgie, Freiburg, Deutschland*,
[2]*Universitätsklinikum Freiburg, Freiburg, Deutschland*

**Background:**
Oncology patients with obesity face particularly complex challenges. In clinical practice, obesity management comes secondary to oncologic care. In fact, weight-loss therapy for several cancers has traditionally been regarded contraindicated. Here, we report on three patients with rectal carcinoma and severe obesity.

**Materials and methods:**
Initial BMI were 65.7, 33.74, 37.8kg/m2. All patients suffered from severe associated comorbidities and qualified for neoadjuvant radio-chemotherapy. Additionally, we discussed these patients in our multi-disciplinary oncologic-metabolic board and recommended multimodal obesity treatment including low-dose GLP-1 receptor agonist Semaglutide in combination with individualized diet and exercise regimens as part of a novel “dual-track” program. At screening and during therapy, we conducted in-depth clinical phenotyping including anthropometric, laboratory and body composition data. Patients were metabolically staged using the Edmonton Obesity Staging System.

**Results:**
All 3 patients achieved clinically relevant weight loss during neoadjuvant therapy (38%, 24%, 10% total weight loss). We observed improvement of physical functionality and dramatic reduction of obesity associated risk factors, including uncontrolled arterial hypertension, severe sleep apnea, and poorly controlled diabetes. We observed an increase in fat-free mass and skeletal muscle. All 3 patients became eligible for and underwent curative oncologic surgery. There were no discernible negative effects regarding tumor progression detected relating to weight loss. Importantly, all three patients experienced a heightened sense of empowerment and engagement over treatment outcomes and exhibited exceptional compliance.

**Conclusion:**
These cases suggest that evaluation of this novel dual-track neoadjuvant therapy may be warranted for selected patients.

### Endoscopic versus percutaneous drainage placement after left-sided pancreatectomy: Multi-state model for defining objectifiable variables on interventional treatment for pancreatic fistula.

ID: 962

**Kategorie: DGAV - Perioperative Medizin: Evidenzbasiertes Handeln**

Matthäus Felsenstein[1], Sophie Dorfer[1], Thomas Malinka[1]

[1]*Chirurgische Klinik, Charité – Universitätsmedizin Berlin, Berlin*

**Background:**
Post-operative pancreatic fistula (POPF) remains a prevalent complication after left-sided pancreatectomy. Despite advances in minimal-invasive surgery, POPF causes unchallenged high morbidity in surgically treated patients and relevantly prolongs in-hospital stay. Interventional treatment is regularly required and relies on interdisciplinary concepts that includes interventional radiology and endoscopy. Evidence on the correct indication and clinical algorithms for available treatment modalities are missing.

**Materials and methods:**
During an observational period of five years (2018-2022), the clinical course from 200 patients after left-sided pancreatectomy at the Department of Surgery, Charité – Unversitätsmedizin Berlin were followed. We identified patients that fulfilled clinical and radiographic criteria for clinically relevant POPF (CR-POPF, n=79, 40.1%). We defined interventional treatment groups which received either percutaneous drainage (PD), endoscopic drainage (ED), and/or re-operation (OP). Patients were grouped in a step-up approach for descriptive analyses and in a stratified, decision-based algorithm for comparative analyses. Comparative analyses considered regression (Kaplan Maier, Logistic regression) and a Cox proportional hazards (Multi state models=MSM) models.

**Results:**
After left-sided pancreatic resection, many patients with CR-POPF needed multiple (n=46; 58.2%) and multimodal (n=39; 49.4%) intervention for achieving final resolution. Based on step-up strategies, majority of individuals (53; 67.1%) were assigned to the ED treatment group. Still, the preferred treatment modality across the variant clinical courses remained conventional percutaneous drainage (PD, n=104, 62.3%). Technical success rates were higher in ED (57.7%) versus PD (32.7%), with resulting in earlier resolution (8.4 days, 95%CI [5.5-11.2d]) when compared to PD (23.4 days, 95%CI [2.8-17.9d]). At any given time point, Hazards of reaching clinical resolution were higher for PD in a MSM analysis (HR 1.385, CI 95% of 0.854, 2.247).

**Conclusion:**
The present study highlights the importance of endoscopy guided, intramural stent placement after left-sided pancreatic resection. We could not discriminate objectifiable, pre- and peri-clinical parameters to be used for optimized treatment allocation such that current clinical decisions must be based on the providers preference and experience.

### Evaluation of Posture in four different robotic surgical systems

ID: 227

**Kategorie: DGAV - Robotergestützte Chirurgie und Innovation: Systemvergleichende Forschung**

Dolores Krauss[1], Stefanie Brunner[1], Jennifer Eckhoff[1], Christian Storms[1], Benjamin Babic[2], Thomas Schulz[2], Jun Kinoshita[3], Thomas Schmidt[1], Wolfgang Schröder[1], Christiane J. Bruns[1], Hans Fuchs[1]

[1]*Klinik und Poliklinik für Allgemein-, Viszeral-, Thorax- und Transplantationschirurgie, Uniklinik Köln, Köln, Deutschland*, [2]*Zentrum für onkologische Speiseröhren- und Magenchirurgie, St. Elisabethen Krankenhaus Frankfurt, Frankfurt, Deutschland*, [3]*Department of Gastrointestinal Surgery, Kanazawa University, Kanazawa, Japan, Kanazawa, Japan*

**Background:**
Although often forgotten and underestimated, especially in a surgical work environment, ergonomics are essential for endurance, efficiency and a long and healthy work life. While many ergonomic challenges faced in open and laparoscopic surgery have been overcome by robotic surgery, new challenges have been created. Nationwide and international guidelines for implementation of an ergonomic workspace are currently lacking. This study aims to identify ergonomic characteristics of a variety of robotic systems to ultimately lay the foundation for ergonomic guidelines and improvements for a healthy workspace.

**Materials and methods:**
Measurements evaluating the surgeon and their interaction with the new technology were applied in either a laboratory or a real-life setting (figure 1). Only surgeons who were sufficiently trained and experienced in the use of the respective system were used for this evaluation. A video camera was used to capture the surgeon at the console during surgery and is positioned in a 90° angle towards the surgeon so that the armrest of the console and the optimal position of the forearms are horizontal in the image. For evaluation of the images, axes are placed through the joints of the surgeon and corresponding angles are calculated based on the position of the individual axes in relation to each other and compared to recommendations for a healthy workspace.

**Results:**
13 surgeries were measured during a real-life setting using the DaVinci (Intuitive Surgical,Sunnyvale,CA,USA), with n=20,250 pictures used for analysis. 2 surgeries in a real-life setting were measured using the Dexter (Distalmotion, Epalinges,Switzerland), with a total of 1994 pictures used for analysis. 17 measurements were taken with the Hugo™RAS System in a simulation setting (Medtronic, Dublin, Ireland), with a total of 1179 pictures used for analysis. A total of 327 pictures from a simulation setting were used for analysis of the Hinotori (MedicaroidCorporation,Kobe, Japan). Results from the evaluation of posture are depicted in table1. Table 2 shows a comparison of the results to the current recommendations for a healthy workplace.

**Conclusion:**
A variety of different robotic systems do not only offer a variety of technical features and advantages but also differ in ergonomic requirements. The neck can be identified as an area of need for improvement for most systems. Future studies, especially in a randomized controlled fashion need to be performed to further analyze ergonomic features of the different systems and guidelines displaying the variety of systems need to be implemented to create a healthy and safe workplace.

Abstracts – DCK 2025 – 142nd Congress of the German Society of Surgery – Munich, March 26–28 • DOI 10.1515/iss-2025-2003
Innov Surg Sci 2025; 10 (Special Suppl 1): s113–s208

*Table 1: Mean, median and range of posture for the variety of systems.*

| DaVinci | | | | | | | |
|---|---|---|---|---|---|---|---|
| | **Knee** | **Hip** | **Shoulder** | **Neck** | **Elbow** | **Back** | **Forearm** |
| **Mean (°)** | 119.4 | 102.3 | 25.1 | 40.7 | 102.2 | 7.5 | 6.7 |
| **Median (°)** | 120.9 | 101.7 | 24.2 | 40.8 | 101.1 | 7.4 | 5.9 |
| **Range (°)** | 141.8 – 73 | 114.9 – 70.8 | 49.3 – 6.6 | 57.3 – 13.2 | 135.7 – 79.7 | 17.6 – 0 | 34.3 - 0 |
| **Dexter** | | | | | | | |
| | **Knee** | **Hip** | **Shoulder** | **Neck** | **Elbow** | **Back** | **Forearm** |
| **Mean (°)** | 117.8 | 102.3 | 45.3 | 33.3 | 119.6 | 1.5 | 14.1 |
| **Median (°)** | N/A | N/A | N/A | N/A | N/A | N/A | N/A |
| **Range (°)** | 110.8 – 124.8 | 98.1 – 106.5 | 43.5 – 47 | 32.2 – 34.4 | 113.1 – 126.1 | 1.1 – 2 | 8.9 – 19.3 |
| **Hugo** | | | | | | | |
| | **Knee** | **Hip** | **Shoulder** | **Neck** | **Elbow** | **Back** | **Forearm** |
| **Mean (°)** | 101.1 | 102.5 | 25.3 | 30.5 | 113.8 | 9.3 | 7.9 |
| **Median (°)** | 92.7 | 98.2 | 27.9 | 31.4 | 117.2 | 9 | 7 |
| **Range (°)** | 74.2 – 142 | 81.8 – 124-2 | 5-1 – 40.4 | 17.1 – 47.1 | 85.6 – 133.9 | 0.4 – 20.6 | 0.6 – 16.8 |
| **Hinotori** | | | | | | | |
| | **Knee** | **Hip** | **Shoulder** | **Neck** | **Elbow** | **Back** | **Forearm** |
| **Mean (°)** | 111.1 | 95 | 30.8 | 11.3 | 99.2 | 5.7 | 15.9 |
| **Median (°)** | N/A | N/A | N/A | N/A | N/A | N/A | N/A |
| **Range (°)** | N/A | N/A | N/A | N/A | N/A | N/A | N/A |

*Table 2: Comparison of the results to the current recommendations for a healthy workplace.*

| | **Knee** | **Hip** | **Shoulder** | **Neck** | **Elbow** | **Back** | **Forearm** |
|---|---|---|---|---|---|---|---|
| **Recommendation (°)** | ≥ 90 | 110-120 | N/A | 10-15 | ≥ 90 | 0-10 | N/A |
| **DaVinci** | | | | | | | |
| **Within recommendation** | Yes | No | N/A | No | Yes | Yes | N/A |
| **Dexter** | | | | | | | |
| **Within recommendation** | Yes | No | N/A | No | Yes | Yes | N/A |
| **Hugo** | | | | | | | |
| **Within recommendation** | Yes | No | N/A | No | Yes | Yes | N/A |
| **Hinotori** | | | | | | | |
| **Within recommendation** | Yes | No | N/A | Yes | Yes | Yes | N/A |

Abstracts – DCK 2025 – 142nd Congress of the German Society of Surgery – Munich, March 26–28 • DOI 10.1515/iss-2025-2003
Innov Surg Sci 2025; 10 (Special Suppl 1): s113–s208

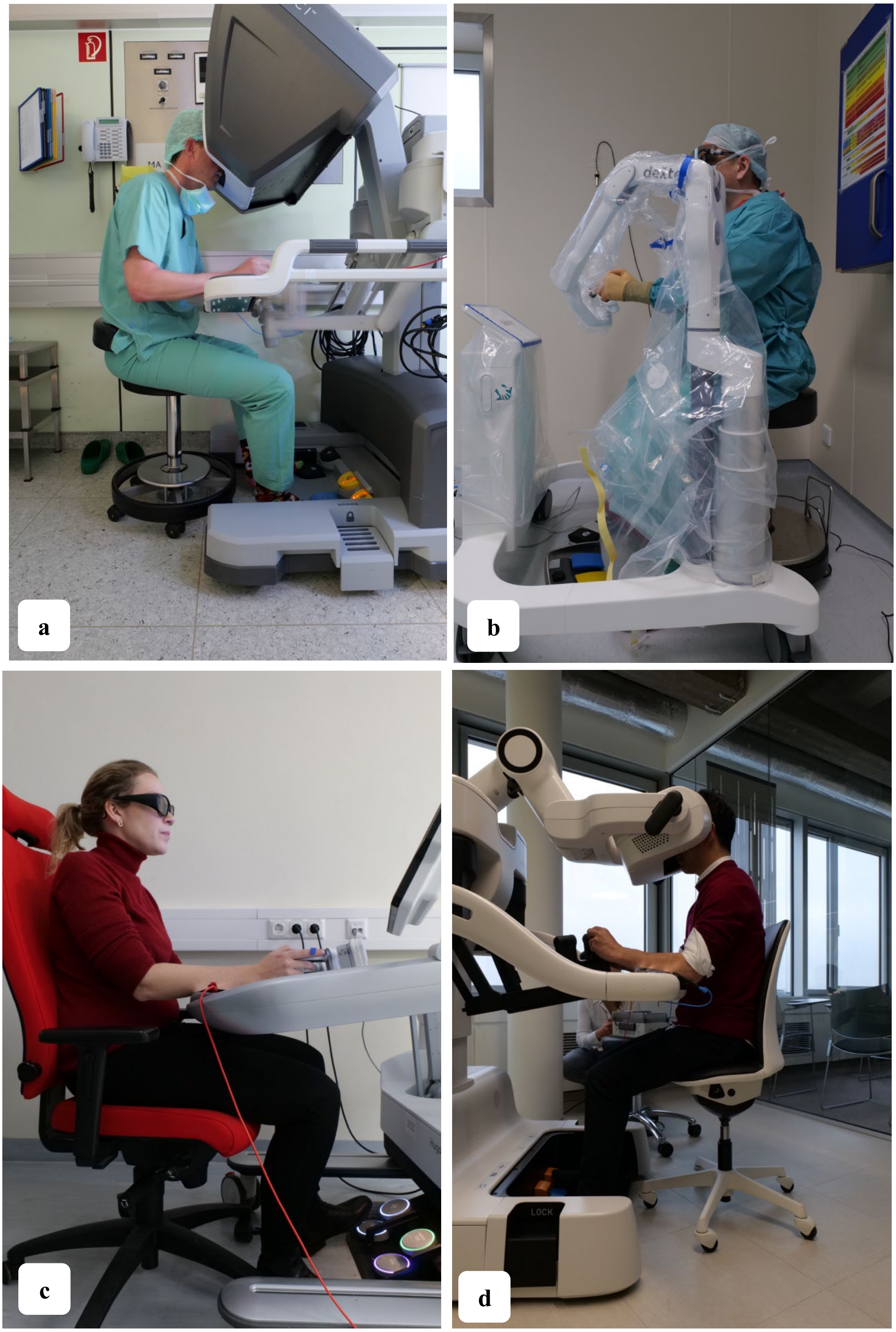

*Figure 1: Measurements evaluating the surgeon and their interaction with the new technology were performed in four different robotic systems – a) DaVinci b) Dexter c) Hugo and d) Hinotori*

### Evaluation of ergonomics and surgeon's stress levels during robotic assisted minimally invasive surgery using a new hybrid robot (Dexter)

ID: 234

**Kategorie: DGAV - Robotergestützte Chirurgie und Innovation: Ergonomie und Stressbelastung**

Dolores Krauss[1], Stefanie Brunner[1], Jennifer Eckhoff[1], Christian Storms[1], Thomas Schulz[2], Christiane J. Bruns[1], Hans Fuchs[1], Benjamin Babic[2]

[1]*Klinik und Poliklinik für Allgemein-, Viszeral-, Thorax- und Transplantationschirurgie, Uniklinik Köln , Köln , Deutschland*, [2]*Zentrum für onkologische Speiseröhren- und Magenchirurgie, St. Elisabethen Krankenhaus Frankfurt , Frankfurt , Deutschland*

**Background:**

Although often forgotten and underestimated, especially in a surgical work environment, ergonomics are essential for endurance, efficiency and a long and healthy work life. While many ergonomic challenges faced in open and laparoscopic surgery have been overcome by robotic surgery, new challenges have been created. Nationwide and international guidelines for implementation of an ergonomic workspace are currently lacking. This study aims to identify ergonomic characteristics of a new hybrid robotic system during a standardized procedure to lay the foundation for a healthy workspace.

**Materials and methods:**

Measurements were performed during a real-life case of a robotic cholecystectomy performed by an experienced robotic surgeon. A video camera was used to capture the surgeon at the console during surgery and is positioned in a 90° angle towards the surgeon so that the armrest of the console and the optimal position of the forearms are horizontal in the image (figure1. For evaluation of the images, axes are placed through the joints of the surgeon and corresponding angles are calculated based on the position of the individual axes in relation to each other and compared to recommendations for a healthy workspace. To evaluate the objective stress level of the surgeon the heart rate variability (HRV) was measured using a chest strap and analyzed using specific scientific software. Furthermore, sound level measurement was performed to evaluate the noise level in the OR. Data was further correlated with the intraoperative course and surgical performance.

**Results:**

N=2 robotic cholecystectomies performed by the same experienced surgeon were measured. Mean sound level was 63.5dB for the first case(range 57.5dB–77.3dB) and a mean of 61.2dB for the second case (range 55.3dB–82dB). Correlation of console versus non console time is showcased in figure2. Mean posture during both procedures in correlation to recommendations for a safe workplace are shown in table1. Mean heartrate, HRV and stress indices are shown in table2. Furthermore, figure3 shows the correlation of heart rate and the intraoperative course.

**Conclusion:**

A variety of different robotic systems do not only offer a variety of technical features and advantages but also differ in ergonomic requirements. The neck can be identified as an area of need for improvement for most systems. Future studies, especially in a randomized controlled fashion need to be performed to further analyze ergonomic features of the different systems and guidelines displaying the variety of systems need to be implemented to create a healthy and safe workplace.

*Table 1: Posture of the surgeon during robotic cholecystectomy compared to recommendations for a safe workplace.*

| Posture | | | | | | | |
|---|---|---|---|---|---|---|---|
| | **Knee** | **Hip** | **Shoulder** | **Neck** | **Elbow** | **Back** | **Forearm** |
| **Mean (°)** | 117.8 | 102.3 | 45.3 | 33.3 | 119.6 | 1.5 | 14.1 |
| **Median (°)** | N/A | N/A | N/A | N/A | N/A | N/A | N/A |
| **Range (°)** | 110.8 – 124.8 | 98.1 – 106.5 | 43.5 – 47 | 32.2 – 34.4 | 113.1 – 126.1 | 1.1 – 2 | 8.9 – 19.3 |
| | | | | | | | |
| | **Knee** | **Hip** | **Shoulder** | **Neck** | **Elbow** | **Back** | **Forearm** |
| **Recommendation (°)** | ≥ 90 | 110-120 | N/A | 10-15 | ≥ 90 | 0-10 | N/A |
| **Within recommendation** | Yes | No | N/A | No | Yes | Yes | N/A |

*Table 2: Measurement of heart rate and stress indices during robotic cholecystectomy.*

| | **Surgery 1** | **Surgery 2** |
|---|---|---|
| **Heart rate** | 103 | 103 |
| **Minimum Heart rate** | 68 | 79 |
| **Maximum Heart rate** | 136 | 124 |
| **PNS* index** | 2 | -2.19 |
| **SNS* index** | 1.89 | 2.92 |
| **Stress index** | 7.5 | 11.9 |

**PNS – parasympathetic nervous system, SNS – sympathetic nervous system*

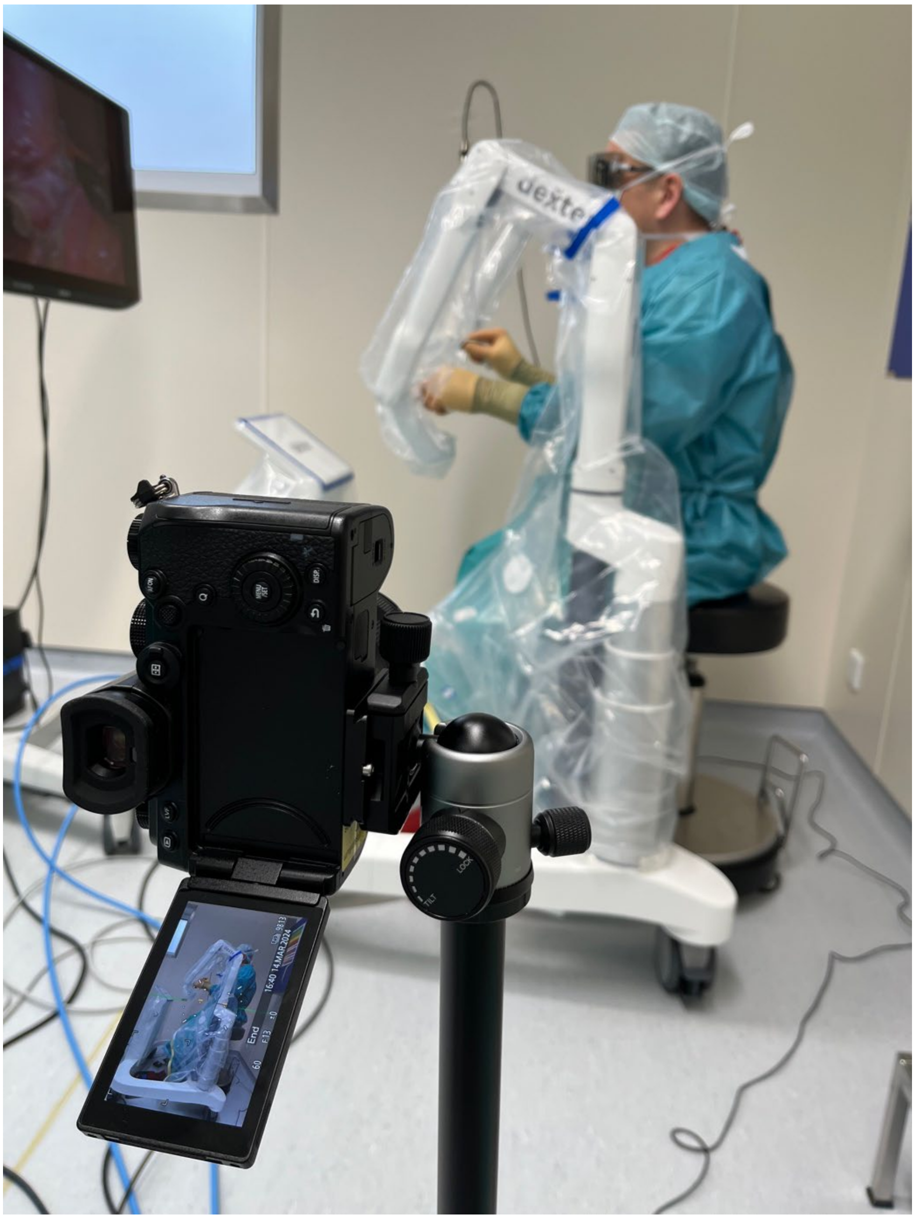

Figure 1: A video camera was used to capture the surgeon at the console during surgery and is positioned in a 90° angle towards the surgeon so that the armrest of the console and the optimal position of the forearms are horizontal in the image

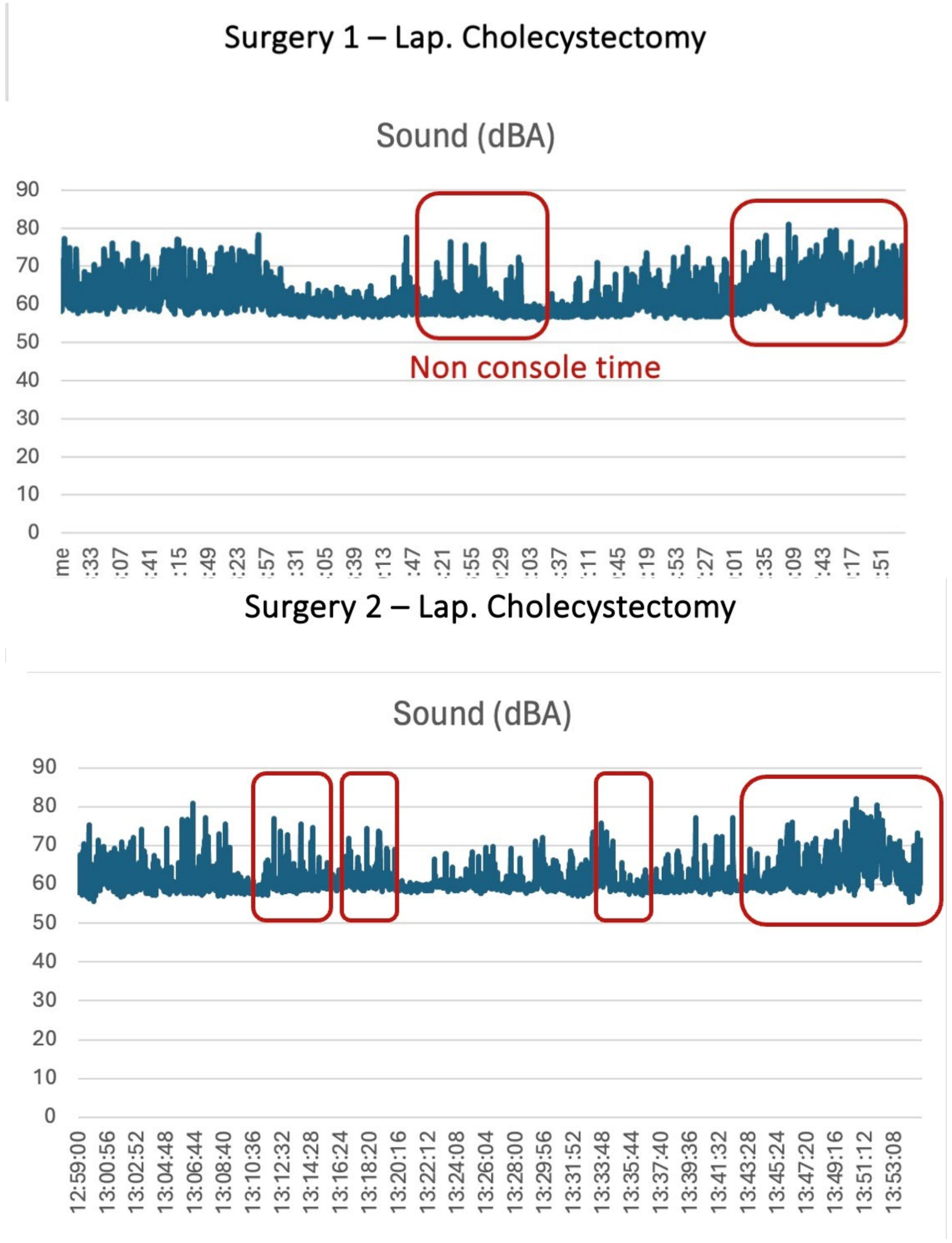

Figure 2: Comparison of sound level during console and non-console time.

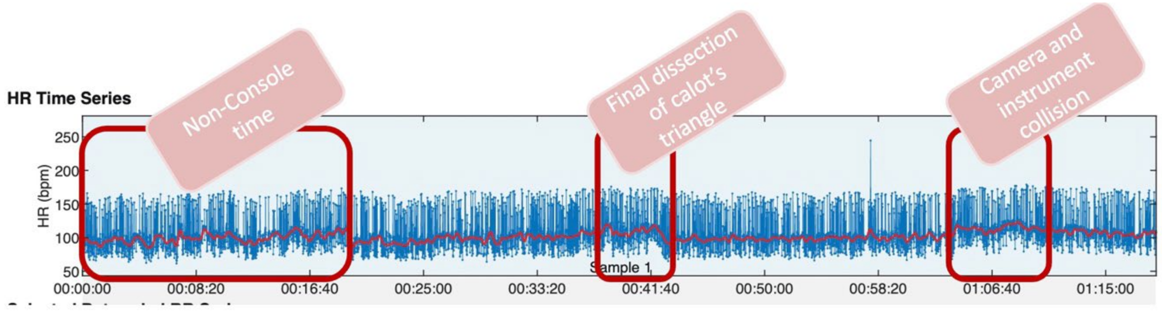

Figure 3: Correlation of heart rate with the intraoperative course. An elevated hear rate was observed during non-console time, final dissection of the calot's triangle and instrument collision.

**Personalized Evaluation and Training of Surgical Ergonomics for Trainees During Robotic Assisted Minimally Invasive Surgery**
ID: 257

**Kategorie: DGAV - Robotergestützte Chirurgie und Innovation: Ergonomie und Stressbelastung**

Dolores Krauss[1], David Sharif[1], Stefanie Brunner[1], Jennifer Eckhoff[1], Lars Schiffmann[1], Benjamin Babic[2], Barbara Seeliger[3], Christiane J. Bruns[1], Hans Fuchs[1]

[1]*Klinik und Poliklinik für Allgemein-, Viszeral-, Thorax- und Transplantationschirurgie, Uniklinik Köln, Köln, Deutschland*, [2]*Zentrum für onkologische Speiseröhren- und Magenchirurgie, St. Elisabethen Krankenhaus Frankfurt, Frankfurt, Deutschland*, [3]*IRCAD, Research Institute Against Digestive Cancer, Strasbourg, France, Strasbourg, Frankreich*

**Background:**
While many ergonomic challenges faced in open and laparoscopic surgery have been overcome by robotic surgery, new challenges have been created. Nationwide and international guidelines for implementation of an ergonomic workspace are currently lacking and there is a need for surgical ergonomics training, especially for trainees in robotic surgery, as a healthy workspace has gained more importance than ever. Aim of the study is to analyze the trainee's posture and thus identify the ergonomic requirements in the robotic operating room from a trainee standpoint. Data is further used to apply an ergonomic training to reduce the workload of surgeons using a robotic device and consecutive evaluation of the training is performed.

**Materials and methods:**
Evaluation of the trainee and their interaction with the new technology was performed during the Robotic Technology Discovery Courses on General Surgery organized by the European Association for Endoscopic Surgery (EAES) at IRCAD, Strasbourg. Data was analyzed on site to give participants individual feedback about their performance and tailored suggestions for improvement. Every participant completed a questionnaire before and after the evaluation. A video camera was used to capture the surgeon at the console during surgery (Figure 1). The camera is positioned in a 90° angle towards the surgeon so that the armrest of the console and the optimal position of the forearms are horizontal in the image (Figure 2). For evaluation of the images, axes are placed through the joints of the surgeon. In particular, the torso, neck, shoulder, hip and knee are considered. Corresponding angles are calculated based on the position of the individual axes in relation to each other and compared to recommendations for a healthy workspace (Figure 3).

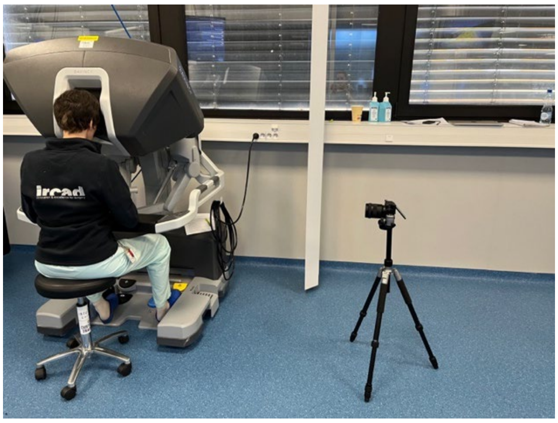

*Figure 1: A video camera was used to capture the surgeon at the console during surgery*

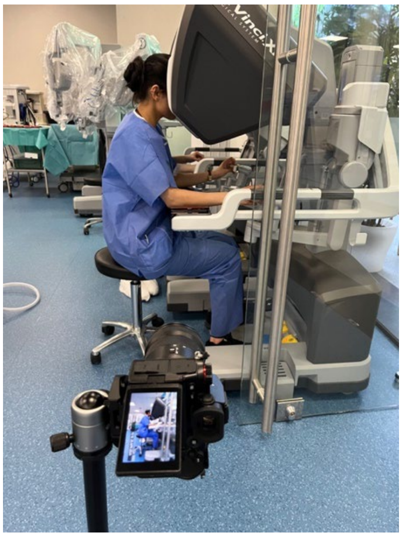

*Figure 2: The camera is positioned in a 90° angle towards the surgeon so that the armrest of the console and the optimal position of the forearms are horizontal in the image*

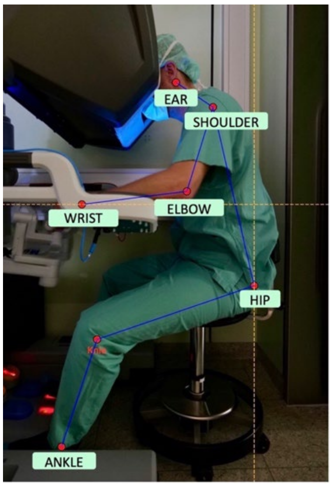

*Figure 3: For evaluation of the images, axes are placed through the joints of the surgeon. In particular, the torso, neck, shoulder, hip and knee are considered. Corresponding angles are calculated based on the position of the individual axes in relation to each other*

**Results:**
A total of 36 participants were measured within two courses in April and July of 2024. Evaluation of the questionnaires revealed that while only 15% of participants (n = 5) had previous experience in the field of ergonomics, 82% (n = 27) expected to learn how to ergonomically use the console during this course. 61% (n = 20) stated to have physical discomfort in a body region where before the exercise no pain was felt, mainly (51%) in the upper body and back. 97% of participants felt that this course helped improve ergonomic skills, 100% stated that they can apply this content to their everyday life. A mean of 159.6 pictures per participant were evaluated in the first course, a mean of 226.8 in the second course. Results of body angles compared to current recommendations for a safe workplace are depicted in table 1.

**Conclusion:**
Evaluation of surgical ergonomics i.e. posture helped identify the ergonomic requirements in a training setting and especially the need for improvement, as recommendations were seldom met. The majority of participants expected to learn about ergonomics during the course real time individual feedback helped improve almost every participant ergonomic skills. Identifying a trainee's individual opportunities for improvement creates the foundation for personalized training and education in surgical ergonomics leading to an improved and healthier workspace for young surgeons and their future careers.

Table 1: Results of body angles compared to current recommendations for a safe workplace.

| N = 36 | Knee | Hip | Shoulder | Neck | Elbow | Back | Forearm |
|---|---|---|---|---|---|---|---|
| **Mean (°)** | 99 | 95.5 | 26.9 | 20.7 | 107.2 | 10.5 | 4.4 |
| **Median (°)** | 104 | 93.4 | 26.7 | 22.4 | 107.6 | 10 | 3.6 |
| **Range (°)** | 54.7 – 134.7 | 81.2 – 110.8 | 1.16 – 43.7 | 3.4 – 34 | 86.8 – 128.1 | 3.2 – 18 | 0 – 21.8 |
| | | | | | | | |
| **Recommendation (°)** | ≥ 90 | 110-120 | N/A | 10-15 | ≥ 90 | 0-10 | N/A |
| **Participants within recommendation** | 23 (63.9%) | 2 (5.6%) | N/A | 4 (11.1%) | 33 (91.7%) | 22 (61.1%) | N/A |

### Clinical implementation of the Dexter Robotic System™ in visceral surgery: A single-centre report on 267 patients

ID: 442

**Kategorie: DGAV - Robotergestützte Chirurgie und Innovation: Ergonomie und Stressbelastung**

Anne-Sophie Hotz[1], Nico Seeger[1], Lukas Gantner[2], Felix Grieder[1], Stefan Breitenstein[1]

[1]*Kantonsspital Winterthur, Winterthur, Schweiz*, [2]*Chirurgisches Zentrum Hirslanden Zürich, Zürich, Schweiz*

**Background:**
Access to surgical robots is increasing in the field of visceral surgery, with several new robotic platforms recently entering the market. The Dexter Robotic System is a multiport, computer-assisted surgical device with two robotic instrument arms, one robotic camera arm, and an open, ergonomic surgeon console. Its modular and simple design aims to provide the robotic dexterity, precision and improved ergonomics, while fully integrating into standard laparoscopic workflows. Notably, Dexter offers surgeons the ability to easily switch between robotic-assisted surgery and laparoscopy when required, as it integrates laparoscopic tower equipment and laparoscopic trocar placements, and allows the surgeon to remain sterile at the surgeon console during surgery. The purpose of this study was to report on the implementation of this innovative robotic system in our visceral surgery department.

**Materials and methods:**
This study reports on the procedures performed with the Dexter System from its introduction at our department in December 2022 to September 2024. All procedures and operative data were prospectively recorded and retrospectively analyzed.

**Results:**
To date, 267 Dexter-assisted elective surgeries (Figure 1) have been performed by 8 surgeons trained to use Dexter according to the manufacturer’s training programme, which included simulator training, dry-lab and wet-lab practice before the first surgery. The most frequently performed procedures were inguinal hernia repairs (n=152) and cholecystectomies (n=78). Furthermore, incisional hernia repairs (n=8), pancreatic resections (n=7), hiatal hernia repairs (n=5) and fundoplication (n=1), as well as colon resections (n=9) were performed at our center. We also explored the feasibility in bariatric surgery (n=2), appendectomies (n=2), rectopexies (n=2), and one hepatic cyst resection. No device-related adverse events occurred. No conversion to open surgery was required. Operative times revealed a fast-learning experience, stabilizing after 10-15 cases, in particular for the cholecystectomies. Even younger surgeons without previous robotic experience were able to get started quickly with a short learning curve.

**Conclusion:**
The study demonstrated that the implementation of the Dexter Robotic System in our visceral surgery department was successful, with an expanding range of procedures, including bariatric, upper gastrointestinal, hepatobiliary, and colorectal surgeries. All Dexter-trained surgeons have been able to perform safe surgeries without conversion to laparotomy, benefiting from Dexter’s ability to swiftly switch to laparoscopy, if necessary. This flexibility is particularly valuable during the learning curve. Our cohort represents the largest dataset reporting the experience with Dexter in a visceral surgery department. Further studies are needed to identify the most effective applications of the Dexter robotic system and to objectively assess its advantages over conventional surgical techniques.

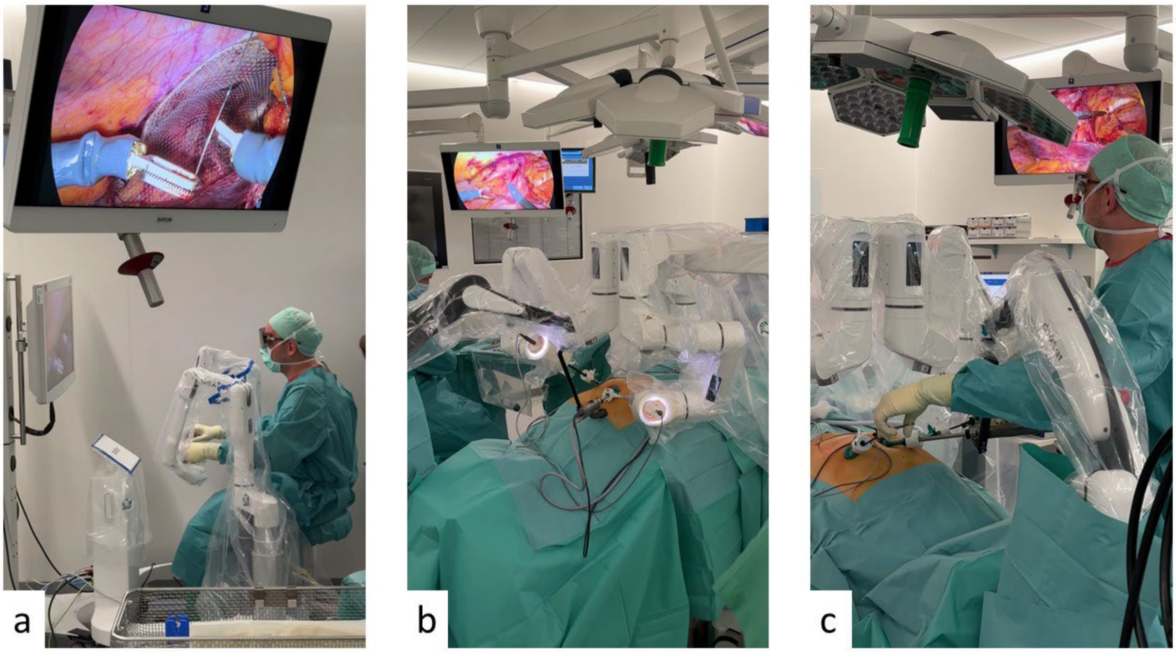

a) the sterile surgeon at the surgeon console; b) Dexter in robotic mode during inguinal hernia repair; c) Dexter with its arm retracted in laparoscopic mode allowing access to the trocars.

### First series of on demand robot surgery using the Dexter Robotic System™ from low to medium complexity cases

ID: 702

**Kategorie: DGAV - Robotergestützte Chirurgie und Innovation: Systemvergleichende Forschung**

Lorenzo MacOne[1], Dolores Krauss[1], Christiane Bruns[1], Thomas Schulz[2], Hans Fuchs[1], Benjamin Babic[2]

[1]*Uniklinik Köln, Köln, Deutschland*, [2]*St. Elisabethen Krankenhaus Frankfurt, Frankfurt, Deutschland*

**Background:**

Although robotic surgery is advancing rapidly, laparoscopic surgery maintains a pivotal role in daily practice. Since 2021 the on-demand Dexter Robotic System™ (Distalmotion, Epalinges, Switzerland), with its surgeon's console, two patient carts with instrument arms, and one endoscope arm offers an alternative compromise between these two techniques. Trocars can be used for both robotic and laparoscopic instruments. This allows for step-specific usage of robotic surgery depending on complexity of the surgical tasks. We present our first data using the Dexter Robotic System™ in a series of low and medium complexity cases.

**Materials and methods:**

Between July 2023 and March 2024, we recruited 19 patients scheduled for minor and middle complexity surgery, in particular inguinal hernia surgery and cholecystectomy. The procedures were performed by two specialized and trained robotic surgeons. The analyzed variables were mean operating time, mean robotic operating time, mean docking-time and draping-time, conversion rate, length of stay, 30-days morbidity and mortality.

**Results:**

From 19 patients operated with Dexter, 4 underwent an inguinal hernia repair and 15 cholecystectomies. For inguinal hernia surgery the mean operative time was 81.25±21.23 minutes, of which the robotic time was 39±10.21 minutes. Mean length of stay was 1,25±0.95 days. For cholecystectomy the mean operative time was 60.6±11.13 minutes, of which the robotic time was 34.2±10.87 minutes. Mean length of stay was 3±3.34 days. The mean docking time was 5.63±3.07 minutes and draping time was 10.58±2.65 minutes. 30-day morbidity was < 1% and only a bile leakage was reported. No 30-day mortality was registered.

**Conclusion:**

This is the first report of Dexter surgery in a non-academic German hospital setting. Feasibility and ease of use were proven. Our results suggest more usage of this surgical robot in non-academic settings with low to intermediate complexity cases or outpatient clinics in Germany.

**The potential of minimal invasive surgery to avoid postoperative neurological complications - a retrospective clinical data analysis with implications to Cancer Neuroscience**
ID: 776

**Kategorie: DGAV - Robotergestützte Chirurgie und Innovation: Systemvergleichende Forschung**

Levi Sochor[1], Ronny Otto[2], Ulf Kahlert[1], Roland S. Croner[1]

[1]*University Clinic for General-, Visceral-, Vascular- and Trans-Plantation Surgery, Medical Faculty University Hospital Magdeburg, Otto-von Guericke University, Magdeburg, Germany, Magdeburg, Deutschland*, [2]*AN-Institute for Quality Assurance in Operative Medicine, Otto-von-Guericke University Magdeburg, Magdeburg, Deutschland*

**Background:**
Neurons represent a potent cellular competent of the tumor microenvironment influencing tumor cells´ intrinsic behavior and their response to external stimuli such as systemic therapies. Little is known how variations in tissue damage level as a consequence of curative-intended surgical therapies play a role in dysregulating physical or systemic neuron to tumor cell interactions. In an attempt to indirectly gather evidences in this research question, we conducted a retrospective clinical data analysis focusing on manifestation of neurological symptoms after surgical interventions. Three prominent neurological diseases in the postoperative course are postoperative deliria, critical illness polyneuropathy and critical illness myopathy. Our aim was to assess different surgical and clinical parameters documented throughout the hospitalization in terms of their particular importance for neurological damage. A special focus was placed on the question of whether minimal invasive operation (laparoscopic and robotic surgery) offers the patient an advantage as compared to laparotomy with regards to the risk of developing postoperative neurological syndromes.

**Materials and methods:**
We did a retrospective data-analysis of 200 patients in total hospitalized throughout the years 2013 to 2023 in the University Medical Center Magdeburg. The majority of the patients are tumor patients. 100 of these patients suffered from the three postoperative neurological syndromes mentioned, the other 100 patients served as a control group with none of the postoperative neurological diseases we examined here. Additionally, the control group patients were selected by not surpassing a duration of hospitalization of 14 days maximum. Such a limit was not applied on the neurological patients group. Our reason for that decision was the particular interest in the question how the duration of hospitalization affects the probability of neurological damage.

**Results:**
The 100 neurological patients consisted of 61 cases with postoperative delirium, 8 cases with critical illness polyneuropathy only, 1 case with critical myopathy only, 19 cases suffering from both critical illness polyneuropathy and critical illness myopathy and 11 cases simultaneously affected by postoperative delirium, critical illness polyneuropathy and critical illness myopathy. The 100 neurological and 100 control group patients comprised both each 70 cases of liver surgery and 30 cases of pancreas surgery to ensure comparability.

Our data analysis showed a significant correlation among the use of open surgical techniques and the elevated occurrence of neurological damage. Moreover, a positive connection could be determined between a long duration of hospitalization and the manifestation of critical illness polyneuropathy and myopathy. The same positive interrelation could be found regarding the duration of ventilation. Furthermore, we noticed that patients with a high complication rate in general tended to develop neurological damage more often than these with few or no postoperative problems. The neurological patients group also significantly suffered more from long and complicated surgical interventions which caused a lot of tissue damage. Especially the occurrence of severe abdominal bleedings and the need of several relaparotomies due to insufficient anastomoses and postoperative infections highlights a strong correlation with the manifestation of postoperative delirium.

**Conclusion:**
There are significant correlation between long, complicated and repeated laparotomies, severe tissue damage, hemodynamic relevant blood losses, a high complication rate, long hospitalization and increased risk of developing postoperative neurological damage. Patients clearly benefit from minimal invasive laparoscopic surgery techniques and a short hospitalization regarding the neurological outcome. Our correlative, retrospective data urgently calls for subsequent wet lab based investigations i.e. on resection material to delineate the altered impact of minimal invasive surgery on neuronal tumor microenvironment as compared to open surgery. Our work indirectly associated to emerging field of Cancer Neuroscience.

Abstracts – DCK 2025 – 142nd Congress of the German Society of Surgery – Munich, March 26–28 • DOI 10.1515/iss-2025-2003
Innov Surg Sci 2025; 10 (Special Suppl 1): s113–s208

### Exploring stress entities for surgery, gynecology and urology with the robotic senhance surgical system versus laparoscopy

ID: 778

**Kategorie: DGAV - Robotergestützte Chirurgie und Innovation: Ergonomie und Stressbelastung**

Vivianda Menke[1], Olaf Hansen[1]

[1]*Evangelisches Krankenhaus Wesel, Department of Surgery, Wesel, Deutschland*

**Background:**
Robotic surgery is revolutionizing traditional surgical procedures, offering precise and minimally invasive techniques. Physical stress is a chronic issue in traditional surgery, drastically impacting health and work-life balance. Therefore, it is time to focus on the surgeon’s stress during surgery, especially in comparison with laparoscopic surgery.

**Materials and methods:**
The present report aims to systematically examine the stress impact on surgeons by comparing the robotic Senhance system to laparoscopic surgery. Based on a multi-sited survey, we seek to conclude results that display representable stress for distinct surgical procedures and different disciplines (e.g., general surgery, urology, and gynecology). By employing the "SURG-TLX" survey, we aim to measure and evaluate the overall stress experience in its various entities. The "SURG-TLX" survey is based on the well-established subjective workload assessment NASA Task load index (“NASA TLX”) and was modified and validated for surgery by Mark R. Wilson. The parametric t-test was used for statistical analysis, and results were displayed as mean and standard deviation. Statistical relevance was defined as p-value ≤ 0.05. The stress dimension scores were calculated per method (robotic vs. laparoscopic surgery, laparoscopic vs. (robotic) bedside, and laparoscopic vs. robotic (cockpit)) and compared between the methods.

**Results:**
Based on the total 350 surgeries, our results show that most stress in robotic surgery (robotic cockpit and bedside surgeon) was experienced regarding Task Complexity (mean= 143±108.25) and Mental Demands (mean = 83.64±83.13). With a focus on laparoscopic surgery (laparoscopic main and assistant surgeon), we found the highest stress ratings for Physical Demands (mean = 140±126.78) and Task Complexity (mean = 126.59±103.81). In contrast, Mental Demands in laparoscopy scored the lowest (mean = 46.97±56.44).
Comparing both methods, our results presented no significant difference in the “Overall Stress” level between robotic (robotic cockpit and bedside surgeon) and laparoscopic (laparoscopic main and assistant surgeon) surgery (p.value = 0.427). However, two of the six stress dimensions indicated a significant difference. Mental Demands were significantly higher in robotic laparoscopic surgery compared to laparoscopic surgery (p.value < 0.001), while Physical Demands were significantly reduced in robotic surgery (p.value < 0.001). Distraction showed a trend of lower robotic ratings than laparoscopic surgery (p.value = 0.090). Stress levels for Temporal Demands, Task Complexity, and Situational Stress were insignificant between groups (p.value ≥ 0.273). A further step in analysis was to compare the stress category results by methods per distinct procedures. This resulted in an additional comparison of three procedures: Cholecystectomy, Radical Prostatectomy, and Total Hysterectomy. Our results revealed Radical Prostatectomy to be the overall most stressful procedure in both robotic and laparoscopic surgery, with a significant difference between the two methods (Robotic: mean = 44.07±19.37, Laparoscopic: mean = 63.79±14.28, p.value ≤ 0.001). Regarding the stress categories, Physical Demands (Robotic: mean=122.7±127.6, Laparoscopic: mean = 228.46±146.26, p.value = 0.006) and Distraction (Robotic: mean = 87.35±102.05, Laparoscopic: mean = 207.69±103.03, p.value ≤ 0.001) during Radical Prostatectomy presented significantly reduced scores in robotic surgery. Cholecystectomy showed significantly lower Task Complexity for robotic surgery (Robotic: mean = 55.48±60.73, Laparoscopic: mean = 120.33±107.55, p.value = 0.041).
Additionally, we explored experienced stress between laparoscopic, (robotic) bedside, and (robotic) cockpit surgeons. Addressing the “Overall Stress” score, cockpit surgeons experienced significantly less stress than laparoscopic surgeons (p.value = 0.018). Mental Demands indicated significantly higher scores for Cockpit surgeons (p.value= 0.015), while Physical Demands were reduced (p.value < 0.001). Further, the trend of reduced Distraction for robotic surgery (p.value = 0.090) reached significance when comparing only cockpit and laparoscopic surgeons (Cockpit: mean = 54.18±60.16, Laparoscopic: 81.38±89.12, p.value = 0.0270). Bedside and laparoscopic surgeons presented an insignificant “Overall

Stress" difference (p.value = 0.065). Within the six stress dimensions, solely Mental Demands reached significance between methods, presenting higher responsibility for robotic bedside surgeons when the senior surgeon is at the cockpit, compared to bedside assistance during laparoscopic surgery (Bedside: mean = 104.03±77.90, Laparoscopic: mean = 46.97±56.44, p.value < 0.001). These numbers display results from cockpit (N = 190) and laparoscopic (N = 63) surgeons, representing the most responsible surgeons during surgery.

**Conclusion:**
The present investigation focused on the crucial issue of stress for surgeons. We found reduced "Overall Stress" for cockpit compared to laparoscopic surgeons. Additionally, we indicate significantly more Mental less Physical Demands, and less Distraction for robotic surgery. A variance through procedures was recognized, and stress levels vary depending on the procedure and surgical specialty. In conclusion, an advantage of robotic surgery compared to laparoscopic surgery could reduce surgeon's stress. This might represent a health benefit, a better, longer work-life balance, and maybe enhanced patient outcome.

### Safety and efficacy of robotic extended totally extraperitoneal repair (r-eTEP) compared to laparoscopic extended totally extraperitoneal (eTEP) repair for ventral hernia: a retrospective propensity score matched comparison

ID: 119

**Kategorie: DGAV - Robotik in der einfachen und komplexen Hernienchirurgie**

Asem Al-Salemi[1], Nader El-Sourani[2], Maximilian Bockhorn[3], Fadl Alfarawan[3]

[1]*Carl von Ossietzky Universität Oldenburg, Fakultät IV - Human- und Gesellschaftswissenschaften, Oldenburg, Deutschland*, [2]*Klinik für Allgemein-, Viszeral und Transplantationschirurgie, Universitätsklinikum Münster , Münster, Deutschland*, [3]*Universitätsklinik für Allgemein- und Viszeralchirurgie, Klinikum Oldenburg, Oldenburg, Deutschland*

**Background:**
The extended totally extraperitoneal technique (eTEP) is a novel approach for ventral hernia repair. This technique has been recently advanced using robotics (r-eTEP). The aim of this study is to perform a comprehensive analysis of the initial results of r-eTEP and to evaluate the safety and efficacy of this technique compared to the eTEP technique.

**Materials and methods:**
This is a monocentric retrospective study of patients with ventral hernias who underwent surgery via eTEP or r-eTEP in our department between 2019 and 2023. Propensity score matching was applied to compare the groups. Preoperative patient and hernia characteristics, intraoperative findings, and postoperative outcomes were subsequently analysed.

**Results:**
Patient demographics were comparable between the groups. The r-eTEP group had a significantly greater proportion of M3 hernias ($p = 0.006$), M4 hernias ($p = 0.020$), incisional hernias ($p = 0.002$), and hernias with rectus diastasis ($p < 0.001$). The r-eTEP group had a significantly larger hernia defect ($p = 0.003$) and larger mesh size ($p=0.015$). The r-eTEP group had a shorter hospital stay ($p < 0.001$) and shorter operative time, though not statistically significant ($p=0.211$). Intraoperative and postoperative complications, as well as postoperative pain, were comparable between the groups.

**Conclusion:**
The findings of the present study show that the r-eTEP technique provides at least similar, if not better results than the eTEP technique. R-eTEP results in a significantly shorter hospital stay and should be favoured over eTEP in more complex cases. The perioperative results, including intraoperative and postoperative complications were comparable for both techniques.

**Preoperative elevation of serum lipase does not increase the risk of postoperative pancreatic fistula in patients with pancreatic ductal adenocarcinoma undergoing pancreatoduodenectomy**
ID: 94

**Kategorie: DGAV - Sichere Viszeralchirurgie für alle**

Ughur Aghamaliyev[1], Gwendolin Seidel[1], Ann-Cathrin Hild[1], Simon Sirtl[2], Athanasios Zamparas[1], Jens Werner[1], Bernhard Renz[1]

*[1]Klinikum der Universität München, Klinik für Allgemein-, Viszeral- und Transplantationschirurgie, München, Deutschland, [2]Klinikum der Universität München, Medizinische Klinik und Poliklinik 2, München, Deutschland*

**Background:**
Preoperative acute pancreatitis (AP) occurs in up to 15% of patients with pancreatic ductal adenocarcinoma (PDAC) at initial presentation. Studies have shown that PDAC patients presenting with AP experience more short-term postoperative complications, particularly postoperative pancreatic fistula (POPF). One of the three main criteria of the revised Atlanta classification for AP is an elevation of serum lipase or amylase levels to three times the normal limit. Since PDAC can elevate lipase levels by causing obstruction or destruction of normal pancreatic parenchyma, differentiating between AP and PDAC-induced abdominal pain can be challenging. It is also not clinically practical to perform imaging, such as CT scans, in every PDAC patient with elevated enzyme activity. This raises the question of whether patients with hyperlipasemia (HL) should be considered as having tumor-induced AP, which carries an increased risk for postoperative complications and may require delaying surgery to treat the AP. However, delaying surgery could also lead to tumor progression. While postoperative AP, defined as elevated serum amylase on postoperative day 2, has been the subject of significant research in recent years, preoperative HL remains relatively understudied. Therefore, this study aims to analyze the impact of preoperative lipase elevation three times the normal limit on postoperative outcomes in PDAC patients undergoing pancreatoduodenectomy (PD).

**Materials and methods:**
Consecutive patients who underwent PD for PDAC at our hospital between January 2014 and December 2023 were retrospectively identified. To ensure a homogeneous study cohort, patients with intraductal papillary mucinous neoplasm-associated carcinoma (IPMN-Ca), preoperatively known chronic pancreatitis, and those with synchronous distant metastases prior to surgery were excluded. Clinical data, including patient demographics, tumor markers, and postoperative complications, were collected for analysis. Preoperative HL was defined as a threefold elevation of preoperative serum lipase levels.

**Results:**
This study comprised 225 patients with available preoperative lipase measurements, who underwent PD for PDAC at our institution between January 2014 and December 2023. Based on preoperative serum lipase levels, patients were categorized into two groups: those with serum lipase elevated up to three times the normal limit (non-HL group, n=171, 76%) and those with preoperative serum lipase elevated over three times the normal limit (HL-group, n=54, 24%). Patients with HL were significantly younger than those without HL (72 vs. 68 years, p=0.04), with an equal gender distribution between groups (50% male, p=0.7). Factors that could potentially lead to acinar damage, such as smoking, alcohol abuse, or preoperative biliary drainage, were not significantly different between the groups. Interestingly, patients with HL had significantly lower rates of preoperative diabetes mellitus (9.6% vs. 31%, p=0.008). Notably, the application of neoadjuvant therapy (NAT) was significantly lower in patients with HL (2% vs. 20%, p=0.001). Other parameters, such as the diameter of the main pancreatic duct, tumor size, and tumor pathological characteristics, were comparable between both groups. Rates of POPF B and C were not significantly different (16% vs. 18%, p=0.7), nor were rates of postoperative hemorrhage (PPH) or delayed gastric emptying (DGE). While complications classified as Clavien-Dindo grade 3b or higher were more frequent in the non-HL group, this difference did not reach statistical significance (29% vs. 17%, p=0.07). Also, 30-day mortality was frequently observed in the non-HL group (overall: 4.4%; 6% vs. 0%, p=0.07).

**Conclusion:**
To the best of our knowledge, this is the first study demonstrating that patients with preoperative HL exhibit unique clinical characteristics and outcomes compared to those without such elevations. Patients with HL do not have increased risk for POPF B/C and may proceed directly to surgery without unnecessary delays, while patients without HL should be closely monitored due to their higher risk factors and increased 30-day mortality.

### Postoperative outcome of inguinal hernia repair performed by surgical residents compared to attendings: a retrospective, single-center analysis

ID: 421

**Kategorie: DGAV - Sichere Viszeralchirurgie für alle**

Helga Oehler[1], Kim Zerr[1], Hanan El Youzouri[1], Ursula Pession[1], Wolf Bechstein[1], Teresa Schreckenbach[1]

[1]*Universitätsklinikum Frankfurt, Zentrum der Chirurgie, Allgemein-, Viszeral- und Transplantationschirurgie, Frankfurt, Deutschland*

**Background:**
Inguinal hernia repair is one of the most common surgical procedures performed worldwide. As part of their training, surgical residents often perform these procedures under supervision, raising questions about the safety and efficacy of their involvement compared to specialists. This study compares the postoperative outcome and complications of inguinal hernia repairs conducted by residents under supervision with those performed by experienced specialists, focusing postoperative complication rates according to the Clavien-Dindo classification and on operation duration.

**Materials and methods:**
This retrospective cohort study was performed at a single primary care center on 388 patients undergoing inguinal hernia repair. The data was collected from 2014 until 2020. The primary outcome was morbidity, according to the Clavien-Dindo classification (CDC), with special attention paid to the surgeon's level of training. The secondary endpoint was operating time and length of hospital stay.

**Results:**
The techniques used for the hernia repair were: Open repair in 'Lichtenstein' technique (n=159) and transabdominal preperitoneal mesh technique (TAPP) (n=228). The hernia repair was mostly performed as an open procedure by residents (n=72; 45%), whereas attendings mostly operated laparoscopically (n=190; 83%). Surgical trainees had significantly longer operation times when performing a laparoscopic procedure, than attending surgeons (mean: 94min vs. mean: 70min; p<0,001). However, there was no difference in operation time when an open procedure was performed (p=0,988). Univariate analyses revealed no influence of the level of training on overall postoperative morbidity (p=0,839). Moreover, patients operated by attendings were more likely to experience numbness (p=0,067) and chronic pain (p=0,077).

**Conclusion:**
In conclusion, while the involvement of surgical residents results in a longer duration for laparoscopic inguinal hernia repair, there is no evidence to suggest a higher risk of postoperative complications. Both resident-performed and specialist-performed surgeries exhibited low complication rates, consistent with the standards of hernia repair outcomes. These findings support the role of resident participation in hernia repairs as safe and effective, with supervision ensuring optimal patient outcomes.

Abstracts – DCK 2025 – 142nd Congress of the German Society of Surgery – Munich, March 26–28 • DOI 10.1515/iss-2025-2003
Innov Surg Sci 2025; 10 (Special Suppl 1): s113–s208

**Does the angle of trocar insertion affect the fascial defect caused? A porcine model**
ID: 586

**Kategorie: DGAV - Sichere Viszeralchirurgie für alle**

Christoph Paasch[1], Jens Meyer[2], Richard Hunger[1], Niklas Krollmann[1], Sophie Heisler[1], René Mantke[1]

[1]*Universitätsklinikum Brandenburg an der Havel, Brandenburg/ Havel, Deutschland*, [2]*Ameos Hospital Schönebeck, Schönebeck, Deutschland*

**Background:**
With an incidence of 0-5.2%, trocar site hernias frequently occur following laparoscopy. It is unclear to what extent the angle of trocar insertion affects the size of the fascial defect caused. Hence, we performed a porcine model.

**Materials and methods:**
In October 2022, a total of five female pigs were euthanized. In alternating order, three bladeless and two bladed conical 12-mm trocars were inserted at an angle of 45° on each side for 60 min twice each pig. For this purpose, an epoxy resin handmade cuboid with a central channel that runs at an angle of 45° was used. Subsequently, photo imaging and defect size measurement took place. The results were compared with those of our previously conducted and published porcine model, in which the trocars were inserted at an angle of 90°. Effects of trocar type (bladed vs. bladeless) and angle on defect size were analyzed using a mixed model regression analysis.

**Results:**
The bladeless trocars caused statistically significant smaller defects at the fascia than the bladed (23.4 (SD = 16.9) $mm^2$ vs. 41.3 (SD = 14.8) $mm^2$, $p < 0.001$). The bladeless VersaOne trocar caused the smallest defect of 16.0 (SD = 6.1) $mm^2$. The bladed VersaOne trocar caused the largest defect of 47.7 (SD = 10.5) $mm^2$. The defect size of the trocars used at a 45° angle averaged 30.5 (SD = 18.3) $mm^2$. The defect size of trocars used at a 90° angle was significantly larger, averaging 58.3 (SD = 20.2) $mm^2$ ($p = 0.007$).

**Conclusion:**
When conical 12-mm trocars are inserted at a 45° angle, especially bladeless ones, they appear to cause small fascial defects compared with insertion at a 90° angle. This might lead also to a lower rate of trocar hernias. Bladeless trocars might cause smaller fascial defects than bladed trocars.

**Successful Surgical Excision of a Large Gastrointestinal Stromal Tumor (GIST) with Metastasis in a Young Adult: A Case Report**
ID: 614

**Kategorie: DGAV - Sichere Viszeralchirurgie für alle**

Baco Stanko[1]

[1]*JZU OB "Dr Mladen Stojanović" Prijedor, Prijedor*

**Background:**
Gastrointestinal Stromal Tumors (GISTs) are rare neoplasms affecting the gastro-intestinal (GI) tract, primarily in middle-aged and elderly individuals.
We present a case report of a young adult who presented with abdominal discomfort and a sensation of pressure while bending forward. Imaging and tissue acquisition revealed a basketball-size, large, well-defined, irregular spherical tumor with two metastases in the greater omentum. Despite the tumors substantial size and proximity to adjacent structures and organs, we successfully excised the tumor entirely (R0) and interruptedly sutured the duodenum after partial D4 resection. The postoperative period was unremarkable, and the patient commenced imatinib therapy, maintaining recurrence-free status for eight months. This case highlights the importance of prompt diagnosis and management of GISTs and demonstrates that complete R0 resection is frequently achievable, even in cases that appear inoperable, with careful surgical planning and execution.

**Materials and methods:**
We present the case of a 24-year-old young man who presented to our surgical ambulance with an indeterminate sensation of abdominal discomfort. The patient's main symptom was an unpleasant feeling of pressure when leaning or bending forward. After months, the symptoms intensified in the week prior the evaluation. No additional complaints had been expressed. The patient was in perfect health, without changes in bowel habits, did not take any daily medications, and had never undergone surgery. There was no familial history of malignant disease. The physical examination revealed a basketball-sized, painless abdominal tumour. During the laparotomy, we observed a well-defined, clearly circumscribed, irregular spherical tumour with many diverticular expansions of different sizes, many newly formed pathological blood vessels, a partially necrotic mass, and a partially hemorrhagic mass 20 x 20 cm in diameter. The tumour has adhered with dense adhasions in adjacent structures and organs. In the larger omentum, we found two metastases, each measuring approximately 8 x 5 cm. We were able to completely remove the tumour from its surrounding structures, only adhering to them and not invading. We performed an oncological (R0) resection along with a partial duodenum's D4 resection, which is the tumour's origin, and used interrupted sutures to stitch the duodenum back together. The postoperative course proceeded without complications, leading to the patient's discharge on the 5th postoperative day. The oncology council reviewed the case and recommended adjuvant treatment with tyrosine kinase-inhibiting agent imatinib mesylate (Gleevec).

**Results:**
GISTs, or gastrointestinal stromal tumors, are a common cancer found in the stomach or small intestine. They are white, well-defined, and firm in consistency, with three main types: spindle cell type, epitheloid type, and mixed-type. Pathological diagnosis is based on morphology and immunohistochemistry, with 80% of GISTs having changes in the c-KIT or PDGF receptor. Cystic changes are more common in malignant, high-grade GISTs.

Surgical management of duodenal GISTs is challenging due to the complexity of the duodenopancreatic area. Surgical alternatives include local resection and pancreaticoduodenectomy, with equivalent disease-free survival rates for both procedures. However, GISTs frequently recur, with a 40% recurrence rate after two years. The management of recurrent illness can be challenging, with repeat curative resection potentially providing a minor survival benefit over palliative chemotherapy.

The survival rate of patients with GIST depends on factors such as the risk category, treatment administered, and likelihood of recurrence following treatment. Individuals with localized GISTs have a 5-year life expectancy of 93%, while those with locally progressed GISTs have a 5-year survival rate of 80%. Non-gastric GISTs have a lower disease-free survival rate compared to gastric GISTs, but overall survival has no significant differences between the two groups.

Neoadjuvant therapy is recommended for advanced, metastatic, and recurrent tumors, with a reduced tumor burden enhancing resection and diminishing surgical morbidity. Overall survival is significantly longer in the surgery plus imatinib group.

**Conclusion:**
GIST is a rare form of tumour; therefore, clinical evidence is insufficient to address numerous clinical and background questions. Fluorodeoxyglucose positron emission computed tomography (FDG PET-CT) scanning is a highly sensitive method for detecting GIST. Treatment modalities for symptomatic GIST encompass pharmacological or surgical intervention, contingent upon the tumour's dimensions, resectability, potential for malignancy, and the degree of metastatic involvement (if present).
The successful surgical excision of the tumour and the metastasis is still the cornerstone of treatment for GIST. We believe this case report is valuable as it demonstrates that complete R0 resection is frequently achievable, even in cases that appear inoperable.

**The expanding spectrum of biliary pathogens**
ID: 719

**Kategorie: DGAV - Sichere Viszeralchirurgie für alle**

Hugo Bonatti[1], Samuel Gavzy[1], Aaron George[2]

[1]*University of Maryland School of Medicine, Baltimore, Vereinigte Staaten*, [2]*Meritus Health, Hagerstown, Vereinigte Staaten*

**Background:**
Most surgeons take bile samples during cholecystectomy only in selected cases. The spectrum of pathogens identified from bile is expanding due to changing demographics, development of antibiotic resistance and improved microbiology technology such as MALDI-TOF allowing for rapid identification of unusual microorganisms.

**Materials and methods:**
The microbiology database of a rural hospital was queried for isolated pathogens from 2018 to 2022. Of the 15451 investigated positive specimens, 147 cultures (135 patients) were from bile.

**Results:**
51.9% of patients were male and median age was 69.3 (27.6 to 99.6) years; 52% were obese (median BMI 30.3kg/m2). Incidence of DM2 was 38%, 55% had hypertension, 50% hyperlipidemia and 45% were smokers. 76% of specimens grew one and 24% multiple organisms. In 24% in addition to pathogens normal flora was reported out, in 4% no aerobic, in 70% no anaerobic growth was observed. Microscopy showed WBC (69%), Gram+ cocci (32%), Gram+ rods (13%), Gram negative rods (39%); no organisms (37%). Staphylococci were isolated from 12 patients with Staphylococcus aureus accounting for 10 (3 MRSA). 20 specimens grew streptococci (50% Group anginosus) and 11 enterococci (2 VRE). In 95 cases Gram negative rods were isolated with E.coli (36) and Klebsiella spp (29) being the most common. Anaerobes were cultured in 20 cases and Candida spp in 10. Most notable, multiple pathogens were isolated that have never been described causing cholecystitis: Bifidobacterium spp, Neisseria subflavia, Peptoniphilus asacharolyticus, Gemella morbillorum, Tissierella praeacuta, Veilonella spp and Candida famata or only very few cases are published (Raoultella planticola (n=3), Hafnia alvei (n=1) or Pantoea agglomerans).sadipscing

**Conclusion:**
More men than women had bile cultured reflecting more complicated cases. The spectrum of organisms causing biliary infections was much more diverse than previously reported and more research regarding these emerging pathogens is warranted.

**Preoperative prediction of postoperative pancreatic fistula after Pancreaticoduodenectomy: Determination and validation of a cut-off value for the Roberts Score.**
ID: 756

**Kategorie: DGAV - Sichere Viszeralchirurgie für alle**

Johannes Kaiser[1], Dietrich Ruess[1], Esther Anna Biesel[1], Franziska Bräuherr[1], Stefan Fichtner-Feigl[1], Sophia Chikhladze[1], Uwe A. Wittel[1]

[1]*Universitätsklinikum Freiburg, Klinik für Allgemein- und Viszeralchirurgie, Freiburg, Deutschland*

**Background:**
Pancreatic cancer has one of the worst prognoses. Surgery is the only curative therapeutic approach. Postoperative complications, especially postoperative pancreatic fistula (POPF), can be life-threatening. Prediction could be a key for improving the perioperative treatment course. Roberts Score is a robust preoperative, multicentre validated instrument based on the preoperative parameters BMI and pancreatic duct width. The aim of this study is the determination and validation of a cut-off value for the Roberts Score for purely preoperative prediction of POPF.

**Materials and methods:**
Retrospectively 582 patients who underwent open and laparoscopic pancreaticoduodenectomy performed between 2002 and 2022 were analyzed. Two cohorts were randomized in an 80:20 ratio. Cut-off value was determined in the exploration cohort (n = 466) using the Youden-Index and its predictive ability for clinical relevant POPF (CR-POPF) were tested in the confirmation cohort (n = 116) using univariant and multivariable regression analysis. POPF was diagnosed according to the latest definition of POPF by the international study group of pancreatic fistula (ISGPF). Included perioperative and oncologic data were collected in a prospectively maintained database at our center.

**Results:**
Both groups were comparable, especially BMI, duct width and the rate of CR-POPF showed no significant differences. The median BMI of all cohorts was 24.7 kg/m2, the mean preoperative duct width as determined by computed tomography was 3.2 mm in exploration and 3.0 mm in confirmation cohort ($p = 0.843$). Overall 42% developed POPF. In the exploratory cohort, there were 187 POPF, of which 110 were clinically relevant (23.6%), compared to 21 CR POPF out of a total of 46 POPF in the confirmation cohort (21.5%; $p = 0.099$). Receiver operating characteristic (ROC) curve of Roberts Score for the exploration cohort showed an area under the curve (AUC) of 0.768 (CI = 0.721-0.815, $p < 0.001$) (Figure 1). A comparable result was shown in the confirmation group with an AUC = 0.732 (CI = 0.631-0.833, $p < 0.001$). The Roberts Score cut-off, as determined by the Youden Index in the exploration cohort, was 0.268 with a sensitivity of 83.5% and a specificity of 41.2%. Positive predictive value (ppv) was 38% and the negative predictive value (npv) was 92% at a significant level of testing ($p < 0.001$). This was confirmed in the confirmation cohort with a cut-off of 0.268 ($p = 0.020$; sensitivity 92%, specificity 48.8%, Youden index 0.433; ppv = 39.4%, npv = 80%). In both the exploration cohort (6d vs. 8d, p=<0.001) and the confirmation cohort with a score > 0.268 (7d vs. 10.d, $p < 0.001$), the time to drain removal was significantly longer. The same was shown for the duration of intensive care (exploration cohort, 4d vs. 5d, $p = 0.036$, confirmation cohort, 5d vs. 6d, $p = 0.049$). The significantly longer hospital stay with a score > 0.268 in the exploration cohort could not be confirmed in the confirmation cohort.
The subsequent multiple logistic regression identified a cut-off value of Roberts Score > 0.268 ($p = 0.038$) as an independent preoperative predictor of CR-POPF.

**Conclusion:**
A cut-off value of 0.268 was identified and successfully validated for the Roberts Score. All variables included in the Roberts Score were independent predictors of POPF after pancreaticoduodenectomy in our study, as was the preoperative cut-off value itself. These findings may improve the clinical use of the score to identify patients for preoperative somatostatin treatment. In addition, the results may be used to stratify patient cohorts for inclusion in studies investigating the development of POPF.

**Is patient age an independent risk factor for postoperative complications in pancreatic surgery?**
ID: 790

**Kategorie: DGAV - Sichere Viszeralchirurgie für alle**

Fabian Nimczewski[1], Manuela Krügermeyer-Kalthoff[1], Jens Höppner[1]

[1]*Klinikum Lippe Detmold, Universitätsklinikum OWL der Universität Bielefeld – Campus Lippe, Universitätsklinik für Allgemein- und Viszeralchirurgie, Detmold, Deutschland*

**Background:**
Pancreatic cysts are an increasingly common entity that leads to surgery. In everyday clinical practice, the question regularly arises to what age pancreatic surgery is reasonable for our patients and whether patient age is an independent risk factor for postoperative complications.

**Materials and methods:**
We present a retrospective data analysis of 27 patients who underwent surgery in the period between 2019 and 2024 due to a benign cystic formation of the pancreas in our center for pancreatic surgery. 11 patients were between 50 and 70 years old at the time of surgery (group 1) and 16 patients were over 70 years old at the time of surgery (group 2). The parameters examined were the anastomotic leak rate, the pancreatic fistula rate, the length of inpatient stay, the length of intensive care stay and the mortality rate.

**Results:**
In group 1, n=6 pylorus-preserving pancreatic head resections, n=3 left pancreatic resections and n=2 enucleations were performed. In group 2, n=8 pancreatic head resections, n=7 left pancreatic resections and n=1 pancreatic segment resections were performed.The anastomotic leakage rate is 9.1% (n=1) in group 1 and 12.5% (n=2) in group 2, with all events being pancreatogastrostomy insufficiency. The pancreatic fistula rate is 9.1% (n=1) in group 1 and 6.3% (n=1) in group 2. The average length of stay in the intensive care unit is 12.7 days in group 1 and 5.2 days in group 2. The average length of inpatient stay is 39.6 days in group 1 and 19.4 days in group 2. The mortality rate is 9.1% (n=1) in group 1 and 12.5% (n=2) in group 2.

**Conclusion:**
Based on the data from our patient collective, it was shown that in the group of over 70-year-olds, there was no significant difference in the rate of anastomotic leakage and pancreatic fistulas when pancreatic resection was performed due to a benign cystic neoplasia. The length of stay in the intensive care unit and the length of inpatient stay were shorter in the group of over 70-year-olds. The mortality rate was higher in the group of over 70-year-olds at 12.5%, but again without statistical significance. After the establishment of minimal invasive techniques and the use of parenchyma-sparing resection procedures, there was a significant reduction in postoperative complications, particularly in older patients.

### Surgery in isolated ileocecal Crohn’s disease – it is time to ask the patients!

ID: 474

**Kategorie: DGAV - Technische Herausforderungen in der Chirurgie von Kolon und Rektum**

Carolina Mann[1], Sara Gronbach[1], Julia Hardt[2], Steffen Seyfried[2], Philipp-Alexander Neumann[3], Hauke Lang[1], Peter Kienle[4], Karoline Horisberger[1]

[1]*Klinik für Allgemein-, Viszeral- und Transplantationschirurgie, Universitätsmedizin Mainz, Mainz, Deutschland*, [2]*University Hospital Mannheim, University of Heidelberg,, Mannheim, Deutschland*, [3]*Department of Surgery, Klinikum rechts der Isar, Technische Universität München, München, Deutschland*, [4]*Theresienkrankenhaus und St. Hedwig-Klinik GmbH, Mannheim, Deutschland*

**Background:**
There is sufficient evidence to suggest that ileocecal resection (ICR) is an alternative to medical therapy for isolated ileocecal CD. However, the optimal timing for surgical intervention remains a topic of debate. Patients are typically managed with medical therapy following a primary diagnosis, most often without surgical options being discussed with them at an early stage. To gain insight into the patient experience, we surveyed individuals who had undergone ileocecal resection. We asked them whether, in retrospect, they would have chosen a different time for resection.

**Materials and methods:**
A survey was administered to patients who had undergone ICR for isolated CD between February 2015 and January 2024. The survey included questions regarding their retrospective time preference for surgery, as well as information and influences on timing at the time.

**Results:**
A total of 79 out of 125 patients (63.2%) have responded to the questionnaire thus far. The median duration of medical treatment for Crohn's disease prior to surgery was six years (range 0.5–20). 47% of all patients (n=37) would have preferred an earlier operation. 48% (n=38) were satisfied with the timing after medical treatment. Two patients had primary surgery, both were satisfied (2.5% of all patients). 2.5% of all patients (n=2) would have preferred a later surgery. 30% (n=11) of all patients who expressed desire for earlier surgical intervention indicated that their gastroenterologists recommended postponement of surgery until the last feasible opportunity, 51% (n=19) stated a lack of awareness of surgical options. 86% of all patients (n=32) belief that earlier surgery would have resulted in earlier symptomatic relief.

**Conclusion:**
The patient argument for pain relief through earlier surgery should be taken seriously by doctors. According to patients, far too few are informed about the possibility of primary resection.

### ChatGPT AND BARD: PROMISES AND CHALLENGES IN ONCOLOGICAL SURGERY

ID: 675

**Kategorie: DGAV - Technische Innovationen und KI in der Onkologischen Chirurgie**

Iris-Iuliana Adam[1 1], Bogdan Moldovan[2], Liliana Vecerzan[1]

[1]*Facultatea de Medicină,ULBS, Sibiu, Rumänien*, [2]*Spitalul Sf. Constantin, Brașov, Rumänien*

**Background:**
OpenAI and Google have introduced ChatGPT and Bard, two AI-driven chatbots that have attracted attention across multiple domains, including oncological surgery. Although these innovations promise advantages like decreased time for research and writing, it's crucial to exercise caution due to potential limitations and errors. Nevertheless, there exist prospects for employing them in medical education and facilitating interactive communication with patients.

**Materials and methods:**
The conducted study evaluated the effectiveness of ChatGPT and Bard for medical specialists, focusing on these tools' capabilities to provide data in scientific research, teaching assistance, and medical support by delivering various information. After analyzing the responses using an objective evaluation system, the degree of relevance of the provided answers to the different questions posed to the chatbots was determined.

**Results:**
The research evaluated the performance of ChatGPT and Bard in various tasks that medical specialists typically undertake, including drafting medical letters, prescriptions, or reports, generating medical content, and preparing students/doctors for different exams. The analyzed responses exhibited differences in accuracy, relevance, and the ability to meet user needs. ChatGPT generally excelled in providing concise and relevant information, while Bard introduced irrelevant or incorrect elements in certain situations.

**Conclusion:**
ChatGPT and Bard have proven to be efficient in performing certain tasks, but their use should be used with caution and in accordance with current regulations to avoid potential drawbacks and risks of disclosing patients' confidential data. Although there are currently some limitations, integrating these artificial intelligence models with the creativity and critical analysis provided by humans they could improve the learning and research process in the field of oncological surgery.

### The Effect of Prophylactic Endoluminal Vacuum Assisted Therapy (pEVAT) to Prevent Anastomotic Leakage of Ultra-low Rectal Anastomoses in High-Risk Patients with Colorectal Cancer

ID: 841

**Kategorie: DGAV - Technische Herausforderungen in der Chirurgie von Kolon und Rektum**

Nour Alkhanji[1 2], Nadja Lehwald-Tywuschik[2 3], Wolfram Knoefel[2]

[1]*Universitätsklinikum Knappschaftskrankenhaus Bochum, Bochum, Deutschland*, [2]*Universitätsklinikum Düsseldorf, Klinik für Allgemein-, Viszeral- und Kinderchirurgie, Düsseldorf*, [3]*AKH Viersen, Viersen, Deutschland*

**Background:**

Colorectal Cancer is the second leading cause of cancer-related deaths. Resection offers the only chance of cure. In cases of ultra-low anterior rectal resections in patients with additional risk factors, an anastomotic leak is the main cause of patients' postoperative morbidity and mortality. In our study we investigated the effect and outcome of prophylactic endoluminal vacuum-assisted therapy (pEVAT) to protect these difficult or "impossible" anastomoses.

**Materials and methods:**

Our study collective comprised 33 patients divided into two groups. All patients in both groups underwent an ultra-low rectal resection for colorectal cancer with an anastomosis measuring 5 cm or less from the anal verge and a diverting stoma. All patients had at least one of the known risk factors for anastomotic leak. The study group included 13 patients who, in addition to the procedure mentioned above, received a pEVAT with an open-pored polyurethane sponge (e.g., Endo-Sponge®), which was carried on for a medium duration of 9 days (range 4-16 days). The control group included 20 patients. Patients' characteristics and risk factors, as well as postoperative complications, were retrospectively collected from medical records and analyzed.

**Results:**

An anastomotic leakage occurred in one (7.7%) of the 13 patients in our study group and 6 (30%) of the 20 patients in our control group. The only patient in the study group with an anastomotic leak was treated with a therapeutic application of EVAT and healing of the anastomosis could be achieved. Applying pEVAT resulted in fewer anastomotic stenoses (7.7% vs. 35%) and a higher rate of stoma reversal (76.9% vs. 55%) in the study group compared to their counterparts in the control group.

**Conclusion:**

This work spotlights the use of prophylactic, endoluminal, vacuum-assisted therapy (pEVAT) as a promising method to prevent anastomotic leak of ultra-low anastomoses in patients with multiple risk factors. However, further prospective studies are needed to reveal the significance of this new method.

**Automated Discharge Letter Generation Using Structured Clinical Data and Large Language Models: A Field Study in Pancreatic Cancer Surgery**
ID: 885

**Kategorie: DGAV - Technische Innovationen und KI in der Onkologischen Chirurgie**

Thomas M. Pausch[1 2], Matthias Ganzinger[2], Ulf Hinz[1], Pascal Fuchs[1 2], Cornelia Lyu[1 2], Nicola Kunz[2], Martin Dugas[2], Rosa Klotz[1]

[1]*Klinik für Allgemein-, Viszeral- und Transplantationschirurgie , Universitätsklinikum Heidelberg, Heidelberg, Deutschland*, [2]*Institut für Medizinische Informatik (IMI), Universitätsklinikum Heidelberg, Heidelberg, Deutschland*

**Background:**
Doctor's letters play a pivotal role in patient care by summarizing essential patient information and facilitating communication between healthcare providers. However, manual letter creation is often time-consuming and prone to errors. Advances in natural language generation using Large Language Models (LLMs) present new opportunities for automating this process. This study aims to compare the manual creation of discharge letters with an automated approach using available structured clinical data and LLMs in the context of pancreatic surgery, evaluating both efficiency and quality.

**Materials and methods:**
A proof of concept for automated doctor's letter generation using LLMs was developed as part of a medical informatics thesis at Heidelberg University Hospital. Building on these findings, a field study was conducted to evaluate the benefits of automated letter generation compared to manual methods. The process involved data exploration, de-identification, LLM selection, prompt engineering, and data preprocessing. Three LLMs were selected for evaluation: OpenBioLLM, SauerkrautLM, and LLaMA3, all of which are capable of generating German medical texts. Special attention was given to prompt engineering to ensure correct medical terminology and the generation of content specific to discharge letters for pancreatic cancer surgery patients.
Five volunteer surgical ward physicians at the European Pancreas Centre (EPC) at Heidelberg University Hospital were tasked with manually writing discharge letters each for patients after pancreatic cancer surgery, while also documenting their time investment. Similarly, automated letters generated by the selected LLMs were reviewed for readability and errors, with the time required for corrections measured. Physicians subsequently completed questionnaires regarding their preference between manual and automated methods. Senior surgical consultants performed a content and risk assessment on the generated letters, evaluating linguistic quality and potential risks of errors. Stakeholder feedback was also gathered from general practitioners and patients to assess the acceptability and comprehensibility of automated letters.

**Results:**
The quantitative analysis of the clinical field study is currently ongoing, and detailed results including quantitative analysis will be presented at the time of the congress. Preliminary findings from the proof of concept demonstrated that automated doctor's letter generation from available structured clinical data using LLMs is feasible, though manual review remains essential. Early indications suggest that the time required for LLM-generated letters is substantially lower compared to manual creation, while maintaining a comparable level of content quality. Physicians expressed mixed preferences, with a trend towards automated generation due to time savings. Stakeholder feedback, including that from general practitioners and patients, indicated a positive reception toward automated letters, with high acceptability and comprehension scores.

**Conclusion:**
Automated doctor's letter generation using LLMs has significant potential to enhance the efficiency of clinical documentation in pancreatic canecr surgery. The reduction in time required for letter creation, combined with comparable quality, suggests that LLMs could help streamline workflows, enabling healthcare professionals to focus more on direct patient care. Additionally, the secondary use of structured clinical data demonstrated the potential for more efficient integration into existing data workflows, highlighting the benefit single-source documentation for clinical use, research, and quality assurance. Continuous model development along with improvements in data quality, are essential to further enhance the accuracy and usability of LLM-generated documents. Adaptation of these models to meet medical standards and address ethical and data protection challenges will be crucial to realize their full potential.

### Undetected Appendicoliths: Implications for Severity and Management of Acute Appendicitis
ID: 919

**Kategorie: DGAV - Technische Herausforderungen in der Chirurgie von Kolon und Rektum**

Maximilian Dölling[1], Mihailo Andric[1], Mirhasan Rahimli[1], Jonas Pachmann[1], Sara Al-Madhi[1], Martin Herrmann[1], Aristotelis Perrakis[1], Roland S. Croner[1]

[1]*University Clinic Magdeburg, Department of General-, Visceral-, Vascular- and Transplant Surgery, Magdeburg*

**Background:**
In cases of uncomplicated acute appendicitis (UAA), the absence of faecoliths in appendicular lumen is often a deciding factor for non-operative treatment (NOT), despite the considerable failure rates linked to this approach. The prevalence of faecoliths has previously been reported to range between 15 % and 38 %, based on retrospective analyses using CT scans, ultrasound, intraoperative palpation, and pathology reports. These methods may, however, miss a number of concrements. This study hypothesizes that the true prevalence of faecoliths is underestimated, potentially contributing to the high treatment failure rate in NOT for appendicitis.

**Materials and methods:**
In a prospective cohort of 56 adult patients diagnosed with acute appendicitis, we introduced intraoperative extracorporeal incision of the vermiform appendix, complementing standard diagnostic tools, to assess the actual presence of appendicular faecoliths.

**Results:**
Our findings demonstrated a 50% increase in the detection of faecoliths through intraoperative examination (n = 36) compared to preoperative imaging (n = 24). Overall, faecoliths were identified in 70 % of patients with acute appendicitis (n = 40).

**Conclusion:**
These results indicate that conventional diagnostic techniques may significantly underestimate the presence of faecoliths, which could explain the elevated rates of treatment failure in non-operative approaches to uncomplicated acute appendicitis.

### Report on Inflammatory Markers in Acute Appendicitis: An Analysis Based on EAES 2015 Guidelines

ID: 933

**Kategorie: DGAV - Technische Herausforderungen in der Chirurgie von Kolon und Rektum**

Maximilian Dölling[1], Mihailo Andric[1], Sara Al-Madhi[1], Mirhasan Rahimli[1], Jessica Stockheim[1], Cora Wex[1], Martin Herrmann[1], Ulf Kahlert[1], Aristotelis Perrakis[1], Roland S. Croner[1]

[1]*University Clinic Magdeburg, Department of General-, Visceral-, Vascular- and Transplant Surgery, Magdeburg*

**Background:**
Acute appendicitis (AA) remains one of the most common surgical emergencies worldwide. Differentiating between uncomplicated acute appendicitis (UAA) and complicated acute appendicitis (CAA) is crucial for determining the optimal management strategy, as non-operative treatment (NOT) may be suitable for UAA, while surgical intervention is typically required for CAA. This study aimed to evaluate the diagnostic utility of two key inflammatory markers—C-reactive protein (CRP) and leukocyte count—in distinguishing UAA from CAA within the framework of the European Association of Endoscopic Surgery (EAES) 2015 classification guidelines.
.

**Materials and methods:**
This retrospective study was conducted at a tertiary care center in Magdeburg, Germany, including 285 adult patients diagnosed with acute appendicitis between January 2019 and December 2021. Inclusion criteria were patients over 18 years of age who underwent appendectomy and had complete laboratory data available. Exclusion criteria were patients who did not undergo surgery or had missing inflammatory marker data. Patient demographics, intraoperative findings, CRP levels, leukocyte counts, and postoperative outcomes were collected. According to the EAES classification, patients were categorized as having UAA or CAA based on intraoperative findings, with CAA including phlegmonous, gangrenous, perforated appendicitis, or abscess formation. Statistical analysis was performed using STATA and SPSS, with significance set at $p < 0.05$. ROC curve analysis was used to assess the diagnostic value of CRP and leukocyte count, with the optimal cutoff for CRP determined via the Youden index.

**Results:**
Of the 285 patients, 193 (67.7%) were classified as having CAA, and 92 (32.3%) had UAA. Inflammatory markers were significantly higher in patients with CAA compared to those with UAA. The median CRP levels were markedly elevated in CAA (60 mg/l; IQR: 20–125) compared to UAA (10.5 mg/l; IQR: 4–32). Leukocyte count was also higher in CAA (14 Gpt/l; IQR: 11–17) than in UAA (13 Gpt/l; IQR: 10–16). ROC curve analysis revealed that CRP had a moderate diagnostic value for detecting CAA with an AUC of 0.80 and an optimal cutoff of 44 mg/l, yielding a sensitivity of 60 % and specificity of 88 %. Leukocyte count, in contrast, demonstrated limited diagnostic utility, with an AUC of 0.6. Patients with CRP levels ≥ 44 mg/l were more likely to develop postoperative complications (OR: 3.0) and had longer hospital stays (OR: 3.5). The median duration of hospitalization was prolonged in CAA (4 days; IQR: 3.0–6.0) compared to UAA (3 days; IQR: 2.5–3.5), with the longest stays seen in patients with perforated appendicitis (7.0 days; IQR: 5–8.5).

**Conclusion:**
CRP is a valuable marker for distinguishing between UAA and CAA in patients with acute appendicitis, with levels ≥ 44 mg/l indicating a higher risk of complications and longer hospitalization. Leukocyte count showed limited diagnostic utility in this context. Incorporating CRP within the EAES classification may aid in the early identification of CAA, guiding appropriate surgical decision-making and improving patient outcomes. Further research is warranted to refine diagnostic thresholds and explore additional markers for risk stratification in acute appendicitis

**Effect of neoadjuvant therapy on endoluminal-vacuum-therapy (EVAC) for anastomotic leakage after esophagectomy**
ID: 45

**Kategorie: DGAV - Therapieindividualisierung in der Behandlung des Ösophagus-/Magenkarzinoms bei vulnerablen Gruppen**

Catharina Fahrenkrog[1], Sorin Miftode[2], Maximilian Bockhorn[2], Nader El-Sourani[3]

[1]*Universitätsmedizin Oldenburg, Oldenburg, Deutschland*, [2]*Klinikum Oldenburg AöR, Universitätsklinik für Allgemein- und Viszeralchirurgie, Oldenburg, Deutschland*, [3]*Universitätsklinikum Münster, Klinik für Allgemein-, Viszeral- und Transplantationschirurgie, Münster, Deutschland*

**Background:**
Oesophageal cancer is the eighth leading cause of cancer and places sixth in worldwide cancer deaths, with incidence rates still rising. Oesophageal cancer is often diagnosed in an advanced stage, with an esophagectomy being the only curative treatment option being left. Even though operation technique and patient care have improved, it poses a great risk for patients, as this operation entails severe morbidity and mortality. Anastomotic leakage is one of the dreaded complications and contributes to a high re-operation rate. Because of its high success rates EVAC has been increasingly used in treatment of anastomotic leakage since its introduction. However, its effectiveness is dependent upon different factors affecting the anastomotic structures. This leaves the question if a neoadjuvant therapy impacts the success rate of EVAC, as most patients receive trimodal therapy because of their preoperative cancer staging. Our aim was to identify any adverse effects of a neoadjuvant therapy on EVAC compared to no prior treatment to improve patient care and treatment algorithms.

**Materials and methods:**
A retrospective cohort study was performed at the Klinikum Oldenburg and included patients with anastomotic leakage after esophagectomy from 2011 until 2024. All patients underwent EVAC therapy and were analysed regarding the success rate as the primary outcome. The length of EVAC, number of sponges needed, the need of converting to another treatment option and the 30-day-mortality were set as secondary outcomes. Patients were compared whether having received a neoadjuvant therapy (either chemotherapy (CT) or radiochemotherapy (RCT)) or none of the above.

**Results:**
Out of 29 patients, 19 have received a neoadjuvant therapy (CT/RCT) and 10 have received no neoadjuvant therapy (NT). There was no significant difference in the success rate of EVAC (CT/RCT n = 15 (78,9%) vs. NT n = 9 (90%), p = 0,454), length of hospital stay (CT/RCT vs. NT: 67,05 vs. 59,5; p = 0,681), the time from oesophageal resection until AI diagnosis (CT/RCT vs. NT: 10,95 vs. 12,6; p = 0,589), the duration of EVAC therapy (CT/RCT vs. NT: 24,11 vs. 23,8; p = 0,964) and the number of sponges needed until defect closure (CT/RCT vs. NT: 6,26 vs. 6,6; p = 0,835) between the two groups. Furthermore, there was no influence on the 30-day-mortality (CT/RCT n = 1 (5,3%) vs. NT n = 1 (10%), p = 0,632), as well as the operative revision rate (CT/RCT n = 1 (5,3%) vs. NT n = 0 (0%), p = 0,46) and the need of conversion to stent therapy (CT/RCT n = 5 (26,3%) vs NT n = 1 (10%), p = 0,303).

**Conclusion:**
In this study there was no significant influence of neoadjuvant therapy on EVAC for anastomotic leakages after esophagectomy, leading to no changes in the management of anastomotic leakage for now. As this cohort was comparatively small, further research must be performed to validate these results. Nonetheless EVAC proved to be an effective and secure treatment option, even though patients required longer hospital stays. In future research patients' safety should always play an important role. Even well-established procedures need continuous reassessments with the aim of constant improvement and the maintenance of high standards.

**Trends in Treatment Strategies for Esophageal Adenocarcinoma in Octogenerians: A Population-based Analysis of 12.224 Patients from the German Clinical Cancer Registry Group**
ID: 575

**Kategorie: DGAV - Therapieindividualisierung in der Behandlung des Ösophagus-/Magenkarzinoms bei vulnerablen Gruppen**

Markus Kist[1], Ulrich Wellner[1], Tobias Keck[1], Michael Thomaschewski[1], Lennart Freiherr-von-Fritsch-Seerhausen[1], Jannis Duhn[1], Stanislav Litkevych[1], Richard Hummel[2], Thaer Abdalla[1]

[1]*UKSH Campus Lübeck, Lübeck, Deutschland*, [2]*Greifswald, Klinik für Chirurgie, Greifswald, Deutschland*

**Background:**
The optimal treatment approach for octogenarians with esophageal adenocarcinoma remains challenging. Due to the presence of comorbidities and increased frailty in this patient group, the decision to pursue tumor resection, with or without multimodal therapy, carries a significant risk of mortality and remains a subject of debate. This study aims to evaluate the suitability of conventional multimodal therapies for elderly patients and to analyze the current state of care across three different age groups of individuals with esophageal adenocarcinoma (EAC) in Germany.

**Materials and methods:**
Data was obtained from 24 clinical cancer registries in Germany delivered by the German Cancer Registry Group of the Association of German Tumor Centers from 2000-2023. We differentiated patients in three age groups (<65 years, 65-79 years, >80 years) and compared them according to the perioperative and long-term survivals based on different modality treatments.

**Results:**
In total 12.224 patients were included and differentiated in three groups (<65 years: n=5643; 65-79 years: n=5260; >80 years: n=1321). From 2000 to 2022, the proportion of patients >80 years old increased from 3% to 14%. UICC I-III distribution was similar for all age groups p= 0.908. Furthermore, >80 years patients were increasingly provided with Chemotherapy (CTx) (<65 years: 54%; 65-79 years: 56%; >80 years: 63%) and surgery only (<65 years: 7%; 65-79 years 10%; >80 years: 17%) compared to younger patients. Overall survival (OS) decreased with higher age group (<65 years: mean 39.6 months; 65-79 years: mean 29.3 months; >80: mean 18 months, p<0.001). In Patients >80 years, the mean OS was longer for surgery only (29 months) compared to CTx (14 months) and definitive radio- chemotherapy (18 months (p<0.005). Multimodal therapy (perioperative Chemotherapy + surgery or radio-chemotherapy + surgery) were underutilized in patients >80 representing only 1% and 7% of all treatment modalities in this age group. However, there was no significanct benefit in survival among multimodal treatment strategies compared to resection only in >80-year-old patients (perioperative Chemotherapy + surgery: p=0.83; radio-chemotherapy + surgery: p=0.56). The 90-days-mortality for esophagus resection was 26% in patients >80 years compared to 5% and 10% for age groups <65 years, 65-79 years. The multivariate analysis shows that the age group >80 is a negative independent prognostic factor for overall survival independent of sex, treatment modality, cN status and resection margin compared to <65 years (HR: 0.45; 95% CI: 0.39-0.53; p<0.001) and 65-79 years (HR: 0.59; 95% CI: 0.51-0.69; p<0.001).

**Conclusion:**
Octogenarians tend to have poorer survival outcomes at similar tumor stages compared to younger age groups. When curative treatment is considered for this age group, tumor resection proves to be more effective than chemotherapy alone or definitive radio-chemotherapy. Additionally, there is no survival benefit for octogenarians undergoing perioperative chemotherapy or neoadjuvant chemoradiation followed by surgery, compared to those who only receive surgery only.

### FLOT versus CROSS of adenocarcinoma of the esophagus and gastroesophageal junction: External Validation of the ESOPEC Trial. A propensity scored matched population-based analysis in Germany

ID: 600

**Kategorie: DGAV - Therapieindividualisierung in der Behandlung des Ösophagus-/Magenkarzinoms bei vulnerablen Gruppen**

Thaer Abdalla[1], Jannis Duhn[1], Stanislav Litkevych[1], Ulrich Wellner[1], Tobias Keck[1], Michael Schweigert[1], Lennart Freiherr-von-Fritsch-Seerhausen[1], Monika Klinkhammer-Schalke[2], Sylke Zeißig[2] [3], Michael Thomaschewski[1], Richard Hummel[2]

[1]*Universitätsklinikum Schleswig-Holstein, Campus Lübeck, Klinik für Chirurgie, Lübeck, Deutschland*, [2]*Network for Care, Quality and Research in Oncology (ADT), German Cancer Registry Group of the Society of German Tumor Centers, Berlin, Deutschland*, [3]*Institute of Clinical Epidemiology and Biometry (ICE-B), University of Würzburg, Würzburg, Deutschland*

**Background:**

Despite the established standard of care for locally advanced esophageal or cardia adenocarcinoma being either perioperative chemotherapy (FLOT) or neoadjuvant chemoradiation (CROSS), the optimal treatment modality remains uncertain. While interim results from the ESOPEC trial favored FLOT, external validation through a multi-institutional analysis is warranted.

**Materials and methods:**

This retrospective cohort study, using data from 24 clinical registries provided by the German Cancer Registry Group, included patients with adenocarcinoma of the esophagus or cardia (cT1cN+ cM0 or cT2-4 cNx cM0) who received neoadjuvant FLOT or CROSS. Propensity score matching was employed to balance baseline characteristics, resulting in a cohort of 820 patients.

**Results:**

Following matching, there were no significant differences between the FLOT and CROSS groups in terms of age, sex, tumor location, or baseline tumor characteristics. After Surgery, CROSS was associated with lower ypT stages ($p<0.001$) and ypN stages ($p=0.001$), and higher pathological complete response rates (20% vs 14%, $p<0.001$). Despite these advantages, FLOT was associated with longer overall survival median 36 months vs 27 months ($p=0.004$) compared to CROSS in the univariable analysis and resulted to be a positive independent prognostic factor in the multivariable Analysis. Moreover, FLOT was associated with lower 90-day postoperative mortality 5% vs 9% and lower rates of distant recurrence in the follow up 30% vs 21% when compared to CROSS. In patients with cN0, there was no survival advantage for patients who received FLOT regime compared to CROSS.

**Conclusion:**

The overall survival of FLOT is superior to CROSS despite the improved pathologic response after CROSS. Especially in the presence of positive nodal disease cN+ at the baseline staging. In case of cN0, both regimes result to have similar survival rates.

**Upfront surgery in esophageal cancer: accuracy of preoperative clinical staging, a retrospective analysis.**
ID: 601

**Kategorie: DGAV - Therapieindividualisierung in der Behandlung des Ösophagus-/Magenkarzinoms bei vulnerablen Gruppen**

Eva Wallner[1], Björn Rudisch[1], Filitsa Farmaki[2], Irina Avramovska[2], Melissa Kemeter[2], Luca Giulini[2], Markus Diener[2], Christina Erhardt-Kreutzer[1], Michael Weitzendorfer[1], Klaus Emmanuel[1], Oliver Koch[1]

[1]*Department of Visceral and Thoracic Surgery, Paracelsus Medical University, Salzburg, Österreich*,
[2]*Department of Surgery, Paracelsus Medical University , Nuremberg, Deutschland*

**Background:**
Treatment of locally limited esophageal cancer is still a frequently discussed topic. Especially, the inaccuracy of clinical staging in lymph node-negative T2-carcinomas has to be considered further in the treatment pathway. Aim of this study was to evaluate the accuracy of clinical staging in patients treated with esophagectomy without neoadjuvant treatment.

**Materials and methods:**
We conducted a retrospective multicenter analysis at the departments of surgery from the university clinics of Nuremberg and Salzburg. Patients between January 2016 and December 2023, who underwent an esophagectomy because of an esophageal adenocarcinoma or squamous cell carcinoma without neoadjuvant treatment were included. Advanced tumor stage, T staging greater than T2, was defined as exclusion criteria.

**Results:**
There were fifty-three patients after upfront esophagectomy included in the analysis.
In 40 (75%) patients a gastroesophageal junction adenocarcinoma was diagnosed, and 13 (25%) patients were treated because of a squamous cell carcinoma of the esophagus.
Clinical T staging was T1 or T2 and all lymph node stages were included.
The endosonographic preoperative staging of invasion depth was accurate in 32 (60%) patients in comparison to the histopathological staging. In 7 (13%) cases the T-values were upstaged one level and in 5 (9%) cases one level downstaged after resection.
The endosonographic preoperative staging of nodal spread was accurate in 32 (60%) patients. Furthermore, in 7 (13%) cases node status was upstaged and in 5 (9%) patients downstaged.
Nine (17%) patients did not undergo a sufficient endosonographic staging, mainly due to prior endoscopic submucosal dissection or stenosis. In these patients, clinical lymph node staging was accurate in 4 patients.

**Conclusion:**
Preoperative staging using endoscopic ultrasound and computed tomography is not accurate enough in a high number of patients, therefore we are seeking to identify other predictive factors underlying advanced stage esophageal cancer for individualized treatment.

**The impact of major surgical complications on FLOT complete responders**
ID: 626

**Kategorie: DGAV - Therapieindividualisierung in der Behandlung des Ösophagus- /Magenkarzinoms bei vulnerablen Gruppen**

Naita Wirsik[1], Paula Möller[1], Lars Schiffmann[1], Nikolai Schleussner[1], Hans Anton Schlößer[1], Hans Fuchs[1], Christiane J. Bruns[1], Thomas Schmidt[1]

[1]*University Hospital Cologne, General, Visceral, Thoracic und Transplant Surgery, Köln, Deutschland*

**Background:**
Since the presentation of the ESOPEC study results at the American Society of Clinical Oncology (ASCO) conference the landscape of the treatment of advanced esophagogastric adenocarcinoma will focus on a multimodal approach with the perioperative chemotherapy according to the FLOT protocol. The influence of major postoperative complications in patients with a full histopathological regression as an optimal oncological outcome is unknown.

**Materials and methods:**
A retrospective analysis of a prospective database from a high-volume center was performed. Primary endpoint was overall survival (OS) of patients with full histopathological regression after perioperative chemotherapy according to the FLOT protocol stratified by anastomotic leakage. Secondary outcomes were other postoperative complications including pylorus spasm or pneumonia.

**Results:**
In total, 65 patients, 9.5% of all FLOT treated patients, showed a complete histopathological regression after FLOT treatment. 15.4% (n=10) of these patients experienced an anastomotic leakage during the postoperative course and 84.6% (n=55) did not. There were no relevant differences regarding gender, age or ASA classification as well as clinical T and clinical N staging between both cohorts (p>0.05). The median survival was not reached in both groups, while the mean survival was 85.5 months (CI 74.3-96.7 months) to 30.4 months (CI 18.3-42.6 months, p=0.007) in favor of patients without an anastomotic leakage. To address the effect of postoperative mortality on OS, the survival analyses was re-performed after exclusion of the 90-day mortality, where an anastomotic leakage was still associated with a worse survival (p=0.04). Comparing survival rates, patients after complete response without anastomotic leakage were observed to have 3-year survival rate of 94% vs 63%, showing the potential immense impact of anastomotic leakages on survival for this specific subgroup. Other frequent postoperative complications including pneumonia, pylorus spasm or wound infections did not show a similar association (p>0.05).

**Conclusion:**
Despite optimal oncological response to FLOT chemotherapy, anastomotic leakage remains a surgical complication with a strong effect on perioperative outcomes as well as potentially long-term patient survival for this specific subgroup. This underlines the importance of further centralization to improve surgical outcomes in general and the investigation of the possible effect of anastomotic leakage on oncological responses of complete responders.

P= 0.007
Survival probability (%)
100
80
60
40
20
0
Anastomotic leckage
None
Yes
None- censored
Yes- censored
0
12
24
36
48
60
72
84
Time (months)

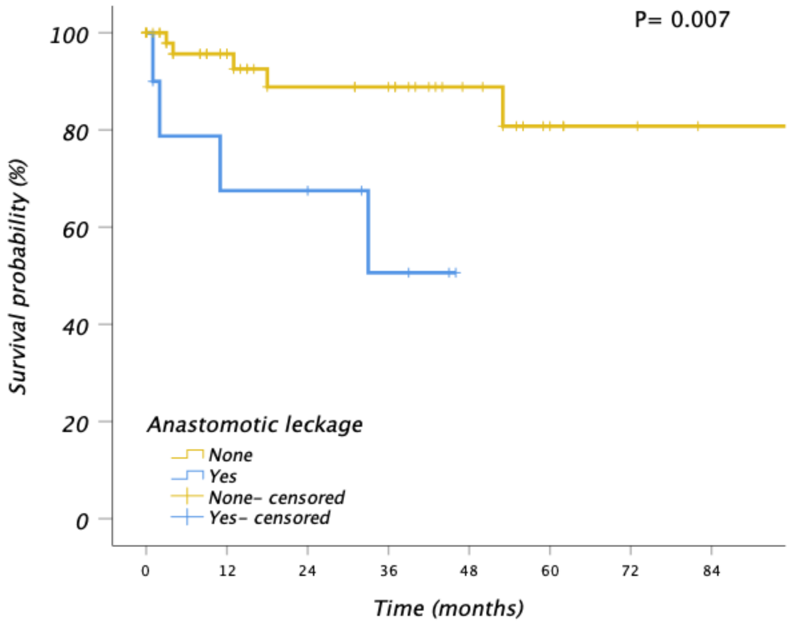

**History of abdominal surgery prior Ivor-Lewis esophagectomy and its impact on complications.**
ID: 689

**Kategorie: DGAV - Therapieindividualisierung in der Behandlung des Ösophagus-/Magenkarzinoms bei vulnerablen Gruppen**

Nikolai Schleussner[1], Julian Lemties[1], Naita Wirsik[1], Kemal Kurt[1], Jin-On Jung[1], Karl Knipper[1], Hans Anton Schlößer[1], Thomas Schmidt[1], Hans Fuchs[1], Wolfgang Schröder[1], Christiane J. Bruns[1], Lars Schiffmann[1]

[1]*Uniklinik Köln, Klinik für Allgemein-, Viszeral-, Thorax- und Transplantationschirurgie, Köln, Deutschland*

**Background:**
Esophagectomy (OE) remains a complex procedure with a significant risk of postoperative complications. One part of the complexity is determined by the abdomino-thoracic nature of the operation. A history of abdominal surgery might even increase this complexity.
This retrospective study explores whether a history of abdominal surgery prior to OE is associated with increased complications after OE.

**Materials and methods:**
997 patients who underwent Ivor-Lewis esophagectomy (IL-OE) for cancer from 2016-2023 at our tertiary center were analyzed from a prospectively maintained database. History of abdominal surgery prior OE where correlated to postoperative complications according to Dindo-Clavien.

**Results:**
History of abdominal surgery was associated with an increased risk of major complications > Clavien-Dindo IIIA (15.1 % vs. 20.6 %, p=0.023). In particular, risk of pulmonary complications as pneumonia (10.7 vs. 14.8, p=0.056) and need for prolonged postoperative ventilation (6.0% vs. 9.2%, p=0.05) were significantly increased in patients with a history of prior abdominal surgery. In patients with history of abdominal surgery the abdominal phase of the Ivor-Lewis procedure was significantly more often performed as an open procedure (7.3 % vs. 13.6 %, p=0.002).

**Conclusion:**
A history of abdominal surgery prior IL-OE is a risk factor for postoperative complications and in particular pulmonary complications. This might be explained by a higher probability of open abdominal surgery in those patients.

Abstracts – DCK 2025 – 142nd Congress of the German Society of Surgery – Munich, March 26–28 • DOI 10.1515/iss-2025-2003
Innov Surg Sci 2025; 10 (Special Suppl 1): s113–s208

### Esophagojejunal Roux-en-y reconstruction – a feasible option in minimally invasive esophagogastrectomy

ID: 977

**Kategorie: DGAV - Therapieindividualisierung in der Behandlung des Ösophagus-/Magenkarzinoms bei vulnerablen Gruppen**

Martin Jazra[1], Nurettin Albayrak[1]

[1]*St. Anna Hospital Herne, General and Abdominal Surgery, Herne, Deutschland*

**Background:**
In total esophagogastrectomy colonic interposition has been established as the preferred method of reconstruction. Jejunal Roux-en-Y, however, can hardly be found in literature.

**Materials and methods:**
In our collective we had to deal with 5 cases of totally minimally-invasive (thoraco-laparoscopic) esophagectomies with no option to use a gastric conduit. In 4 patients tumor involvement of the stomach was extended, in 1 patient gastric perfusion was insufficient.

In all 5 patients we found suitable vascular architecture of the small bowel shown by diaphanoscopy using two light sources and ICG. The jejunal loop was carefully created after identifying and temporary occlusion of at least 3 intestinal branches. The jejuno-jejunostomy was performed laparoscopically either antecolic or retrocolic.

In all cases the azygos vein was ligated and the esophagojejunal anastomosis was created above the azygos level thoracoscopically.

**Results:**
All cases could be completed minimally invasive, no leakage occurred. All patients left our hospital within 10 days. We experienced to cases of incarcerated enterothorax.

**Conclusion:**
In selected patients esophagojejunal Roux-en-y serves as an alternative reconstruction instead of colonic interposition in total esophagogastrectomy. It can be performed minimally invasive.

**Hyperspectral imaging for organ quality assessment of a machine-perfused donor liver**
ID: 266

**Kategorie: DGAV - Transplantation: Neue Entwicklungen**

Mohamed El-Mahrouk[1], Cord Langner[2], Robert Sucher[1], Daniela Kniepeiss[1]

[1]*Medizinische Universität Graz, Allgemein-, Viszeral- und Transplantationschirurgie, Graz, Österreich*,
[2]*Medizinische Universität Graz, Diagnostik- & Forschungsinstitut für Pathologie, Graz, Österreich*

**Background:**
Hyperspectral Imaging (HSI) has been successfully used to assess the quality of transplanted kidneys and livers. However, further research is necessary to evaluate the benefits of HSI technology in machine perfused organs.

**Materials and methods:**
The liver from a 49-year-old female donor underwent normothermic machine perfusion before transplantation. Assessment of organ quality was conducted using laboratory values pathology and HSI.

**Results:**
The donor liver's size (29x17x11cm) and weight of 2180 grams posed challenges for adequate placement within the organ container. During perfusion lactate levels steadily rose, peaking at 4.9 mmol/l after the total perfusion time of 12 hours. Macroscopic alterations on the liver's surface were noted, particularly pronounced in segments 2, 3, and 8. HSI of these areas unveiled significantly reduced oxygenation. Consequently, based on all these observations, the decision was made to discard the organ. Histological examination of the altered regions revealed congestion, necrotic changes, and dissociation of sinusoidal lining cells from liver cell cords. The histological findings correlated well with the HSI.

**Conclusion:**
HSI could be a useful additional tool in assessing donor liver quality before transplantation. Before acceptance of a large organ for normothermic machine perfusion, size, and weight should be evaluated.

### Understanding Patients´ Information Requirements Prior to Abdominal Organ Transplantation

ID: 837

**Kategorie: DGAV - Transplantation: Neue Entwicklungen**

Teresa Linge[1], Lisa Felgendreff[2], Ulrike Bode[1], Wilfried Gwinner[3], Richard Taubert[4], Eva Baumann[2], Moritz Schmelzle[1], Elena Link[5], Philipp Felgendreff[1]

[1]*Department of General, Visceral and Transplant Surgery, Hannover Medical School, Hannover, Deutschland*, [2]*Department of Journalism and Communication Research, Hannover University of Music, Drama, and Media, Hannover, Deutschland*, [3]*Department of Nephrology and Hypertension, Hannover Medical School, Hannover, Deutschland*, [4]*Department of Gastroenterology, Hepatology, Infectious Diseases and Endocrinology, Hannover Medical School, Hannover, Deutschland*, [5]*Department of Communication, Johannes Gutenberg University, Mainz, Deutschland*

**Background:**

Abdominal organ transplantation is a highly complex and non-routine procedure that requires thorough patient education. Due to the complexity of the process, patients have numerous questions about aspects such as waiting times, preparation, the surgery itself, and post-transplant care. Understanding patients' informational needs and preferred sources is crucial for helping them navigate the transplantation process. Satisfying those information needs can help setting realistic expectations, and fostering a sense of control. While informational resources from patient organizations exist in Germany, they often lack specificity for certain organs like liver or kidney transplants. More tailored educational materials are needed to support patients in making informed decisions and feeling adequately prepared. These materials must be distributed via the preferred communication channels so that they reach the patients. However, there is a lack of detailed understanding regarding the required specific support and informational needs of patients in Germany awaiting abdominal organ transplantation, which is crucial for ensuring they feel fully prepared.

**Materials and methods:**

Since April 2024, a cross-sectional survey has been conducted at the Hannover Medical School's transplant outpatient clinic. Patients awaiting liver or kidney transplants completed a self-administered questionnaire. They answered questions about their informational needs (e.g., about the underlying disease) using a 5-point Likert scale (1 *not at all* to 5 *very extensive;* adapted from Link & Memenga, 2023), their use of various informational sources (e.g. “books”; 1 *never* to 5 *very frequently;* adapted from Link et al., 2023) and the quality of their communication with doctors at the transplant center (e.g., “I was given the opportunity to ask the questions I wanted to ask”; 1 *never* to 5 *always;* Lu et al., 2024). Further topics were addressed, but were not part of the current analysis. Responses were analyzed descriptively.

**Results:**

Between April and October, 86 patients (41 awaiting kidney transplants, 45 awaiting liver transplants) participated, with ages ranging from 19 to 73 years (M = 50.13, SD = 13.86). 55.81% were male and 44.19% female. The main sources of information were healthcare providers (M = 3.45, SD = 1.06, 95% CI [3.22, 3.67]), and the educational materials provided to them (M = 2.92, SD = 1.20, 95% CI [2.66, 3.17]). Internet searches were also used (M = 2.88, SD = 1.26, 95% CI [2.60, 3.15]). Patients actively sought information about their underlying disease (M = 3.63, SD = 1.25, 95% CI [3.36, 3.90]), the transplantation process (M = 3.36, SD = 1.49, 95% CI [3.03, 3.68]), potential complications (M = 3.19, SD = 1.40, 95% CI [2.88, 3.49]), and the organ distribution system (M = 2.54, SD = 1.38, 95% CI [2.24, 2.84]). They expressed a strong interest in gaining information on how they can actively contribute to the success of the transplantation (M = 3.50, SD = 1.48, 95% CI [3.18, 3.82]) and in finding ways to maintain their overall well-being despite the associated challenges (M = 3.70, SD = 1.43, 95% CI [3,40, 4.00]). Patients reported positive experiences regarding the communication with the doctors at the outpatient clinic (M = 4.31, SD = 0.88, 95% CI [4.12, 4.50]).

**Conclusion:**
Initial results indicate that while healthcare professionals remain the primary source of information, patients frequently turn to the internet for additional guidance. Moreover, they expressed a strong interest in actively participating in the success of their transplantation. There is a clear demand for reliable educational materials that empower patients to take an active role in their transplant journey and improve self-efficacy. The study's final results will provide more comprehensive insights, allowing for a more tailored approach to addressing patients' needs.

**Bibliography:**
Link, E., Baumann, E., & Aluttis, C. (2023). Ein geschlechterspezifischer Blick auf das gesundheitsbezogene Informationshandeln, seine Treiber und Barrieren. *Bundesgesundheitsblatt - Gesundheitsforschung - Gesundheitsschutz*, *66*(10), 1153–1162. https://doi.org/10.1007/s00103-023-03757-6

Link, E., & Memenga, P. (2023). Digitale, personalisierte Gesundheitsinformationsangebote von Ärzt*innen: Ergebnisse einer Befragung von Patient*innen und Ärzt*innen zu Akzeptanz und Anforderungen. *Bundesgesundheitsblatt - Gesundheitsforschung - Gesundheitsschutz*, *66*(9), 982–989. https://doi.org/10.1007/s00103-023-03750-z

Lu, Q., Link, E., Baumann, E., & Schulz, P. J. (2024). Linking patient-centered communication with cancer information avoidance: The mediating roles of patient trust and literacy. *Patient Education and Counseling*, *123*, 108230. https://doi.org/10.1016/j.pec.2024.108230

### Outcome of the novel description of Arterial position Changes after Major Liver Resections: retrospective study

ID: 864

**Kategorie: DGAV - Transplantation: Neue Entwicklungen**

Sepehr Abbasi Dezfouli[1], Mohammadamin Shahrbaf[1], Philipp Mayer[2], De-Hua Chang[2], Arianeb Mehrabi[1], Katharina Hellbach[2]

[1]*Department of General, Visceral and Transplantation Surgery, Heidelberg University Hospital, Heidelberg, Germany, Heidelberg*, [2]*Department of Diagnostic and Interventional Radiology, Heidelberg University Hospital, Heidelberg, Germany, Heidelberg*

**Background:**

After major liver resections, anatomical shifts due to liver parenchymal hypertrophy and organ displacement can happen. The aim of this study was to evaluate the impact of these anatomical changes on the main abdominal arteries (coeliac trunk and superior mesenteric artery) and on patient outcomes.

**Materials and methods:**

All patients who underwent major liver resections (between January 2010 and July 2021) and who underwent preoperative and postoperative arterial-phase contrast-enhanced abdominal CT imaging were studied. Observed arterial position changes were classified into three groups: no position changes; class I position changes (vessel displacement with or without kinking with a vessel angle greater than 105°); and class II position changes (kinking less than or equal to 105°). The Mann–Whitney test and the Kruskal–Wallis test were used to compare continuous variables and the chi-squared test and Fisher's exact test were used to compare categorical variables. Univariable and multivariable logistic regression analyses were used to identify the risk factors for morbidity and mortality.

**Results:**

A total of 265 patients (149 men and median age of 59 years) were enrolled. Arterial position changes were detected in a total of 145 patients (54.7%) (99 patients (37%) with class I position changes and 46 patients (18%) with class II position changes) and were observed more often after extended resection and right-sided resection ($P < 0.001$). Major complications were seen in 94 patients (35%) and the rate of mortality was 15% (40 patients died). Post-hepatectomy liver failure ($P = 0.030$), major complications ($P < 0.001$), and mortality ($P = 0.004$) occurred more frequently in patients with class II position changes. In multivariable analysis, arterial position change was an independent risk factor for post-hepatectomy liver failure (OR 2.86 (95% CI: 1.06 to 7.72); $P = 0.038$), major complications (OR 2.10 (95% CI 1.12 to 3.93); $P = 0.020$), and mortality (OR 2.39 (95% CI 1.03 to 5.56); $P = 0.042$).

**Conclusion:**

Arterial position changes post-hepatectomy are observed in the majority of patients and are significantly related to postoperative morbidities and mortality.

**Meta-analysis of incident and therapy for anastomosis bile leakage after liver surgery and liver transplantation**
ID: 869

**Kategorie: DGAV - Transplantation: Neue Entwicklungen**

Sepehr Abbasi Dezfouli[1], Elmira Heidenreich[1], Mohammadamin Shahrbaf[1], Arianeb Mehrabi[1]

[1]*Department of General, Visceral and Transplantation Surgery, Heidelberg University Hospital, Heidelberg, Germany, Heidelberg*

**Background:**

Bile leakage is a significant complication following liver surgery and liver transplantation, with potential consequences for patient morbidity and prolonged hospitalization. While various techniques for bile duct anastomosis have been developed, bile leakage remains a frequent issue. Understanding the incidence and optimal management strategies is essential. This meta-analysis aims to evaluate the incidence, risk factors, and therapeutic interventions for bile leakage following liver surgery and transplantation.

**Materials and methods:**

The search was conducted in the PubMed, Scopus, Web of Science, and Embase databases up to end of September 2024. Eligible studies were studies that assessed the incidence and management of anastomosis leakage in hepatic resection and liver transplantation after biliary reconstruction. The primary endpoints were the incidence of biliary leakage and the secondary endpoint assumed postoperative managements. The meta-analysis was performed using STATA software.

**Results:**

In liver transplantation, the pooled incidence of duct-to-duct anastomotic leakage was 5% (95% CI: 4-6%), and bilioenteric anastomosis (BEA) leakage was 6% (95% CI: 5-8%). After liver resection, BEA leakage incidence was 8% (95% CI: 5-11%). Management strategies for duct-to-duct leakage showed reoperation success at 43% (95% CI: 33-54%), while conservative (1%, 95% CI: 0-5%), percutaneous (11%, 95% CI: 4-21%), and endoscopic methods (9%, 95% CI: 2-18%) were less effective. For BEA leakage after transplantation, reoperation had a 69% (95% CI: 41-92%) success rate, while conservative (7%, 95% CI: 0-24%), percutaneous (4%, 95% CI: 0-17%), and endoscopic (0%) approaches were less effective. In liver resection, reoperation (41%, 95% CI: 0-94%) and conservative management (41%, 95% CI: 0-99%) were equally successful, with percutaneous (8%, 95% CI: 0-26%) and endoscopic (0%, 95% CI: 0-2%) methods showing lower effectiveness. Reoperation was consistently the most effective treatment.

**Conclusion:**

Bile leakage remains a significant complication after liver surgery and transplantation. Reoperation consistently proves to be the most effective management strategy, while conservative, percutaneous, and endoscopic approaches show lower success rates across both liver transplantation and resection cases. Further prospective studies are needed to approve our results.

**Impact of Hypothermic Oxygenated Machine Perfusion on Immune Cell Clearance in Liver Transplantation: Enhancing Graft Function and Post-Transplant Outcomes**
ID: 940

**Kategorie: DGAV - Transplantation: Neue Entwicklungen**

Dominik Koch[1 1], Malte Schirren[1], Severin Jacobi[1], Moritz Drefs[1], Markus Guba[1], Dionysios Koliogiannis[1]

[1]*LMU Klinikum München , Klinik für Allgemein-, Viszeral- und Transplantationschirurgie, München, Deutschland*

**Background:**
Hypothermic oxygenated machine perfusion (HOPE) has emerged as a critical innovation in liver transplantation, offering significant protection against ischemia-reperfusion injury (IRI). This study aims to quantify and characterize immune cells flushed out during HOPE to explore its effects on graft function and post-transplant outcomes.

**Materials and methods:**
Fifty liver grafts underwent end-ischemic HOPE. Perfusate samples were collected at three time points: at the start of perfusion, after 10 minutes, and at the end of perfusion. The samples were analyzed to quantify and characterize immune cells, assessing the effectiveness of HOPE in reducing cellular debris and its impact on graft quality.

**Results:**
The primary perfusate contained significant concentrations of immune cells, including segmented neutrophils, lymphocytes, and monocytes. After 10 minutes of perfusion, cell concentration decreased by over 95%, and by the end of perfusion, more than 99% reduction was observed.

**Conclusion:**
HOPE effectively reduces immune cell concentrations in liver grafts, suggesting a mechanism for improved graft function and reduced post-transplant complications. These findings support the continued use and optimization of HOPE in liver transplantation.

**Effect Of Donor Resuscitation On Early Liver Graft Survival In Donation After Brain Death**
ID: 961

**Kategorie: DGAV - Transplantation: Neue Entwicklungen**

Georgios Polychronidis[1], Ercan Gönan[1], Markus Mieth[1], Arianeb Mehrabi[1], Arash Nickkholgh[1,2]

[1]*Ruprecht-Karls University of Heidelberg, Department of General, Abdominal and Transplant Surgery, Heidelberg, Deutschland*, [2]*Klinikum Ludwigshafen, Klinik für Allgemein-, Viszeral- und Thoraxchirurgie, Ludwigshafen am Rhein, Deutschland*

**Background:**
The effect of cardiac arrest and resuscitation on graft survival after Donation after Brain Death (DBD) has not been extensively researched.

**Materials and methods:**
We present preliminary data from 1563 consecutive liver transplants from a single center from December 1998 to December 2020. We conducted both univariable and multivariable analyses to identify predictive factors for graft survival in organs from donors who underwent resuscitation compared to those who did not.

**Results:**
We identified 93 (6%) DBD after resuscitation. Median resuscitation time was 10 (1-90) minutes. 3-month and 1-year graft survival for recipients of postresuscitation grafts was 90% ,83% and 85%, 73.5% for no post-resuscitation donor grafts. (log rank p = 0.18). In the multivariable analysis donor age was identified as a risk factor for graft survival (HR=1.014; 95% CI: 1.009- 1.019).

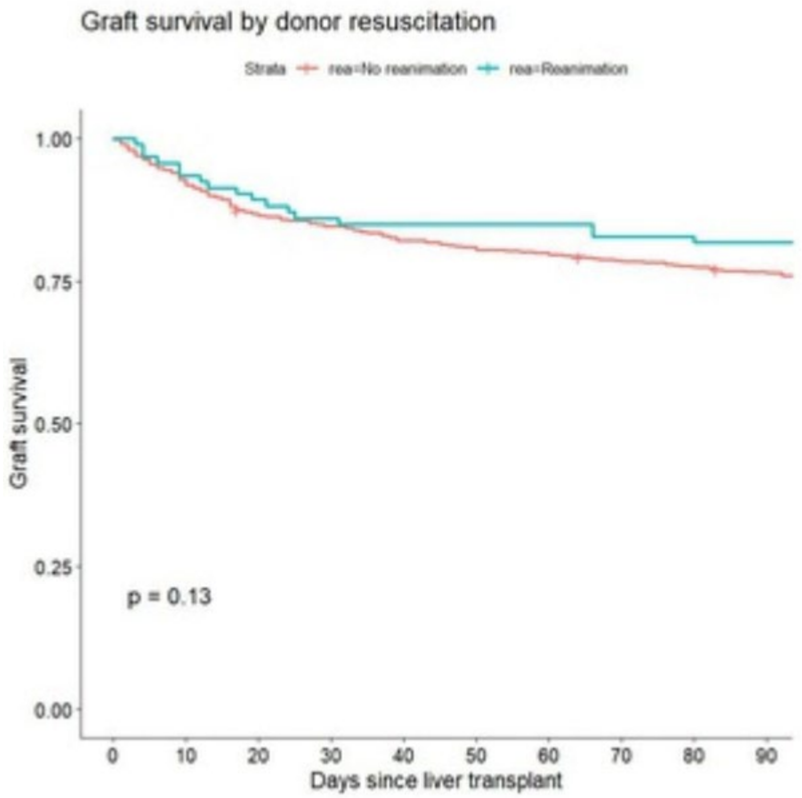

**Conclusion:**
Thoughtful selection of these donors represents a prudent strategy for broadening the donor pool.
